# First Example of Fluorinated Phenanthroline Diamides: Synthesis, Structural Study, and Complexation with Lanthanoids

**DOI:** 10.3390/molecules27154705

**Published:** 2022-07-23

**Authors:** Nane A. Avagyan, Pavel S. Lemport, Konstantin A. Lysenko, Alexey O. Gudovannyy, Vitaly A. Roznyatovsky, Valentine S. Petrov, Mikhail F. Vokuev, Yuri A. Ustynyuk, Valentine G. Nenajdenko

**Affiliations:** Department of Chemistry, M. V. Lomonosov Moscow State University, 119991 Moscow, Russia; nane.avakyan@mail.ru (N.A.A.); lemport.pavel@yandex.ru (P.S.L.); klyssenko@gmail.com (K.A.L.); alexeygudovannyy@gmail.com (A.O.G.); vit.rozn@nmr.chem.msu.su (V.A.R.); vs.petrov25@gmail.com (V.S.P.); vokuevmihail@mail.ru (M.F.V.); yuriustynyuk@gmail.com (Y.A.U.)

**Keywords:** fluorine, phenanthroline, nucleophilic substitution, complex, lanthanoid, structure

## Abstract

An efficient approach to the synthesis of diamides of 4,7-difluoro-1,10-phenanthroline-2,9-dicarboxylic acid was elaborated. Direct nucleophilic substitution with 4,7-dichloro-1,10-phenanthroline precursors opened access to difluoro derivatives in high yield. As a result, four new fluorinated ligands were prepared in up to 88% yield. Their structure was proved by a combination of spectral methods and X-ray data. A set of lanthanoid complexes was prepared to demonstrate the utility of new ligands. The structure of the complexes was studied in solid state (IR-spectroscopy, X-ray diffraction) and in solution (NMR-spectroscopy).

## 1. Introduction

**Figure 1:**
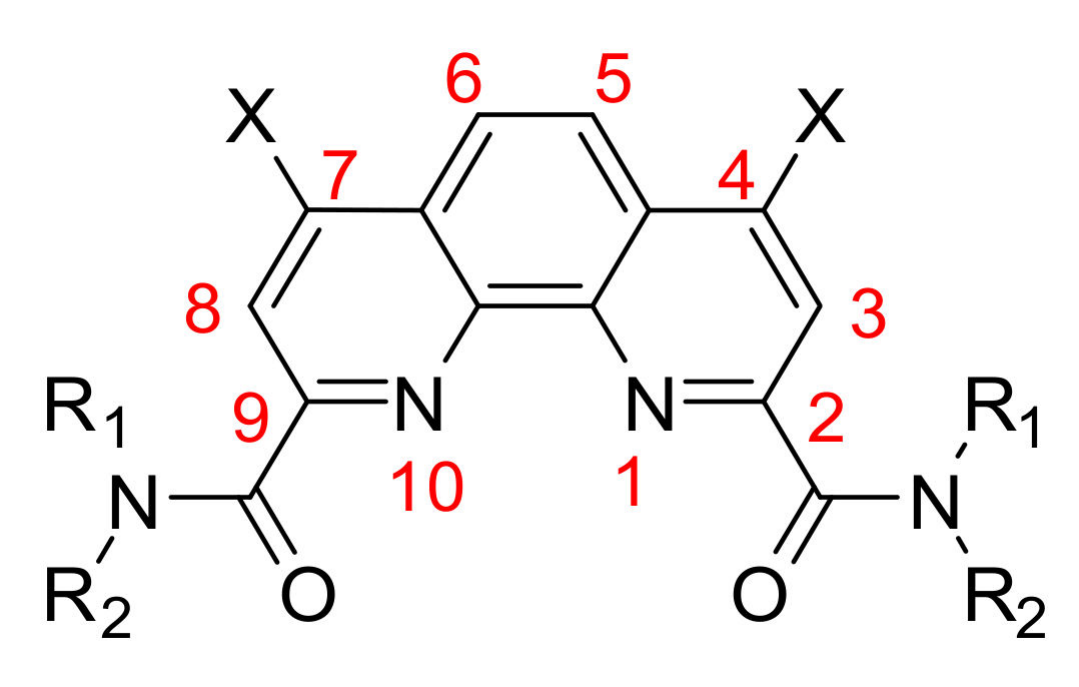
The general structure of diamides of 1,10-phenanthroline-2,9-dicarboxylic acid.

Currently, nuclear power plays very important role in global electricity production. However, the resulting high-level wastes (HLW) are a deterrent to the further development of this industry [1]. Known approaches to recycle the HLW involve the use of nitrogen-containing ligands capable of forming organosoluble complexes with *f*-elements. One of the most popular and widely studied classes of such compounds are heterocyclic amides [2]. 2,9-Diamides of 1,10-phenanthroline dicarboxylic acid are unique polydentate *N*,*O*-ligands. Their structure contains “soft” coordination centers (aromatic nitrogen atoms) and hard coordination centers (carbonyl oxygen atoms) simultaneously (Figure 1) [3,4,5].

The complexing and extraction properties of these ligands can be varied by changes in the structure of substituents in both the amide function and the phenanthroline core. On the other hand, such structural diversity permits fine-tuning of the ligand properties. Recently, serious attention has been paid to such phenanthroline diamides which have various substituents in the aromatic core, such as chlorine atoms [6,7,8,9], alkoxy groups [3,10], and others.

The presence of two chlorine atoms opens the way to further modification of ligands using nucleophilic substitution reactions. This approach may be of use for reducing the Brönsted basicity of the synthesized ligands. However, the presence of chlorine atoms is undesirable in terms of the resistance of the extractants to radiolysis [11,12].

The introduction of fluorine atoms into the target molecule changes such parameters as lipophilicity, solubility, binding to receptors, metabolism, membrane permeability, acid-base characteristics, conformational properties of compounds, resistance to oxidants, and environmental influences. All these unique features are used as a working tool in the creation of new medicines and materials [13,14,15,16,17]. Furthermore, the replacement of chlorine atoms with fluorine atoms can be considered as one of the possible ways to increase the radiation resistance, since the C-F bond strength (115.7 kcal/mol) is significantly higher than the C-Cl bond energy (77.2 kcal/mol). The incorporation of a fluorine atom into a strictly defined position of an organic molecule is one of the key problems in the synthetic chemistry of fluorine-containing compounds. Due to the progress in the field of selective fluorination, trifluoromethylation, and the introduction of small fluorinated fragments into the target structure, many practical tasks have been seriously developed [18,19,20,21,22,23,24,25,26].

Despite the exceptional importance of fluorinated heterocyclic compounds, only a few examples of fluorinated 1,10-phenanthrolines have been described to date. Thus, some 5-fluoro substituted 1,10-phenanthrolines were prepared [27] by Skraup–Debner–Miller reaction using the corresponding fluorinated derivatives of 2-nitroaniline. In [28], the synthesis of copper coordination compounds based on 5-fluoro- and 5,6-difluorophenanthrolines was reported and it was shown that such complexes have a cytotoxic effect on cancer cells, while their toxicity to healthy cells (fibroblasts) is lower. 5,6-difluorophenanthroline has been also prepared [29] by electrolysis of 1,10-phenanthroline using a triethylamine hydrofluoric acid complex (Et_3_N·6HF) as a fluorinating agent. Recently [30], 2-fluoro-1,10-phenanthroline was obtained in 89% yield from the corresponding chlorine-containing precursor by exposure to a 20-fold excess of KF in the presence of 3 equiv. of 18-crown-6 in a DMSO medium at 110 °C for 96 h. Later [31], isomeric 5-fluoro-1,10-phenanthroline was synthesized by a similar method. 3,8-difluoro-5,6-diphenyl-1,10-phenanthroline [32] was prepared in 58% yield by the Rh(III)-catalyzed reaction of 2,2′-bipyridine *N*-oxides with internal alkynes. Palladium complexes of phenanthroline fluorinated at 4-position were obtained and luminescence properties of both ligands and their complexes were studied [33]. Some 4-fluorinated derivatives of 1,10-phenanthroline were prepared using intramolecular cyclization of fluorine-containing building blocks [34]. In spite of some progress in the field of fluorinated phenanthrolines, there are no efficient and general methods for their synthesis. It is important to note that to date there are no examples of 1,10-phenanthroline-2,9-dicarboxylic acid diamides containing fluorine atoms in the aromatic cores.

This paper is devoted to the study of the synthesis of 4,7-difluorinated phenanthrolines starting from the corresponding dichloro derivatives. The nucleophilic substitution of chlorine for fluorine [35] using cesium or potassium fluorides have been demonstrated as an efficient approach to fluorine-containing *N*-heterocycles [36]. The advantages of this method are the economy of the fluorinating agent and the ability to work without strict exclusion of air and moisture.

## 2. Results and Discussions

### 2.1. Synthesis and Structure of Fluorinated Phenanthrolinediamide

**Scheme 1:**
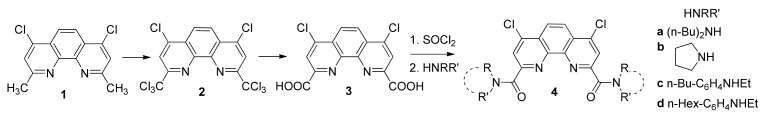
Synthesis of diamides **4a**–**d**.

We used 4,7-dichloro derivatives of phenanthrolinediamides **4** as precursors for the synthesis of fluorinated phenanthrolinediamides. The currently known method for synthesis of **4** is a multistage transformation based on 4,7-dichloro-2,9-dimethylphenanthroline **1**, which, in turn, can be obtained by a well-known method from Meldrum acid and *ortho*-phenylenediamine (Scheme 1) [8].

**Scheme 2:**
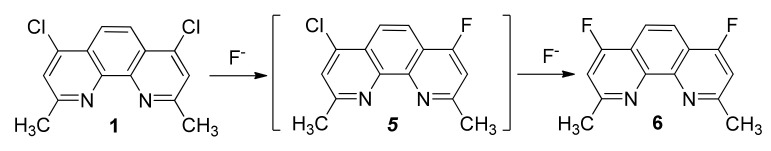
Synthesis of 4,7-difluoro-2,9-dimethylphenanthroline **6** by fluorination of **1**.

We started our research by studying the nucleophilic substitution of chlorine atoms in 4,7-dichloro-2,9-dimethylphenanthroline **1** using potassium and cesium fluorides as nucleophiles (Scheme 2). We carried out numerous model experiments to find the conditions for this transformation using various solvents, fluorinating agents, and reaction times. Periodic monitoring of the reaction was performed by NMR spectroscopy. The data obtained indicate that the reaction proceeds completely within 24 h in the case of large excess of a calcined cesium fluoride in dry DMSO at a temperature of 110 °C.

**Figure 2:**
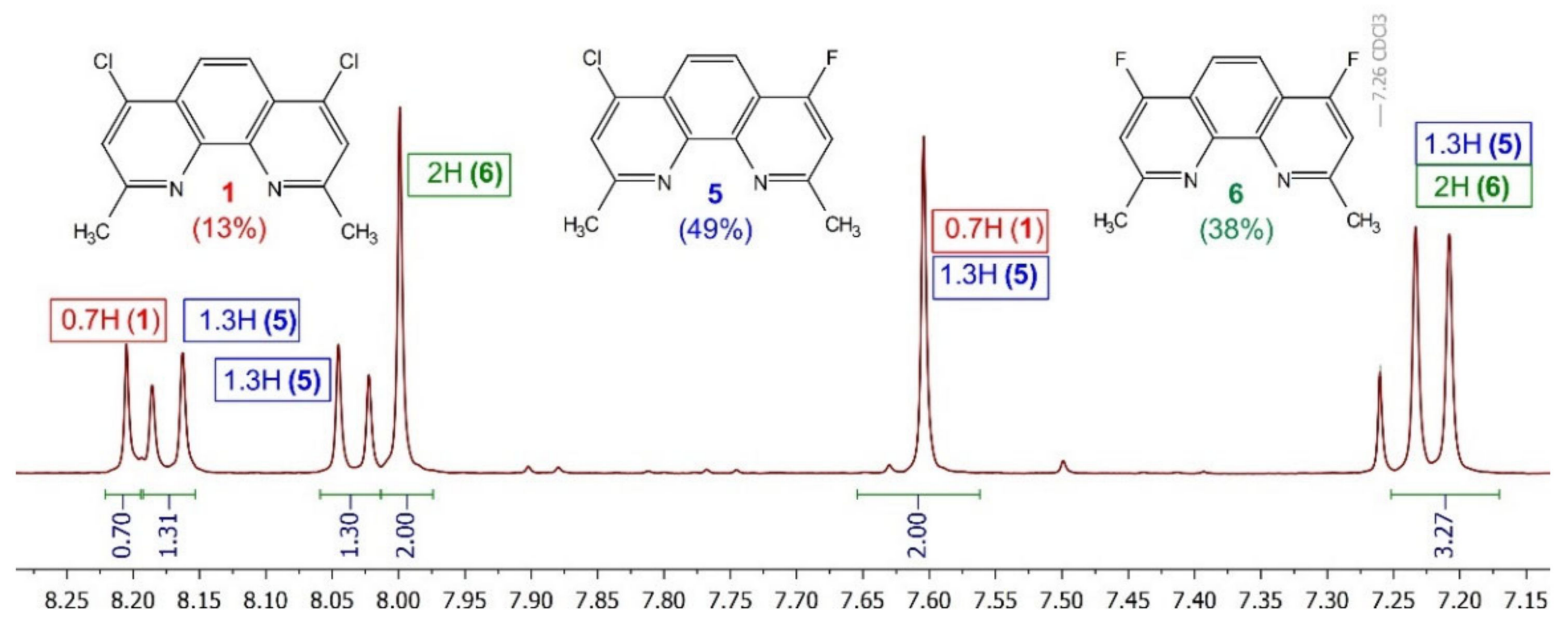
^1^H NMR spectrum (CDCl_3_) of the mixture of **1**, **5**, and **6**.

Signals of initial phenanthroline **1**, intermediate monofluorinated phenanthroline **5**, as well as the desired fluorine-containing phenanthroline **6** can be distinguished due to significant difference in chemical shifts of **1**, **5**, and **6**. Figure 2 shows a typical spectral pattern for a reaction mixture of these products upon isolation from the reaction mixture after 8 h of heating at 110 °C.

In accordance with the selected conditions, fluorinated phenanthroline **6** was obtained in a yield of 33%. The product is a yellowish powder, noticeably soluble in dichloromethane, chloroform, acetone, and other organic solvents. Traces of moisture negatively affect the yield of **6** due to competitive nucleophilic substitution of halogen atoms with water to form hydroxy derivatives. Isolation and purification of compound **6** is complicated due to its tendency to sublimate. In addition, the intermediate product **5** has similar chromatographic mobility and solubility.

**Figure 3:**
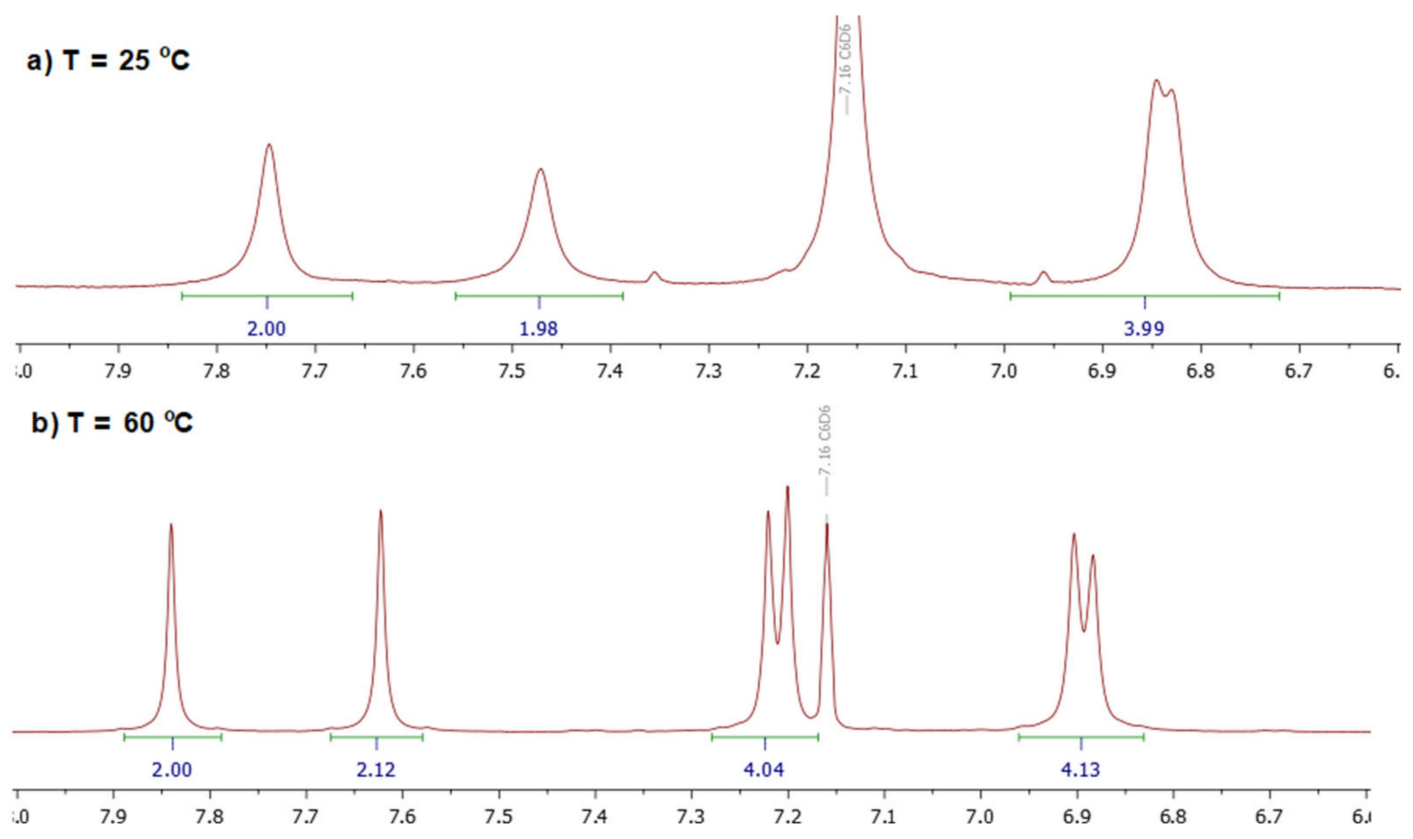
Fragments of ^1^H NMR spectra of substance **4c** in C_6_D_6_ at (**a**) 25 °C and (**b**) 60 °C.

Thus, it became necessary to find another, more effective approach to fluorinated phenanthroline diamides. We decided to study nucleophilic substitution for chlorinated diamides **4a**–**d**. These diamides were prepared using literature methods [8]. We obtained compound **4c** for the first time (see the Experimental part and ESI). Ligand **4c** is a white powder, highly soluble in chloroform, dichloromethane, ethyl acetate, acetone, and benzene. Due to the amide rotation of [37] substituents, the NMR spectra of compound **4c** are predictably complicated. Nevertheless, it is possible to achieve reasonable spectra in C_6_D_6_ (Figure 3).

Next, we started investigation of nucleophilic fluorination of **4a**–**d**. Taking into account the results obtained during the fluorination of compound **1**, we selected CsF as the fluorinating agent and dry DMSO as the solvent. It was found, using ^19^F NMR monitoring of the reaction mixture, that 4 h of heating at 80 °C leads to complete conversion of **4a**–**d** ligands. As a result, we isolated fluorinated ligands **7a**–**d** as white or yellowish powders in 74–88% yields. Some characteristics of these substances are given in Table 1.

**Figure 4:**
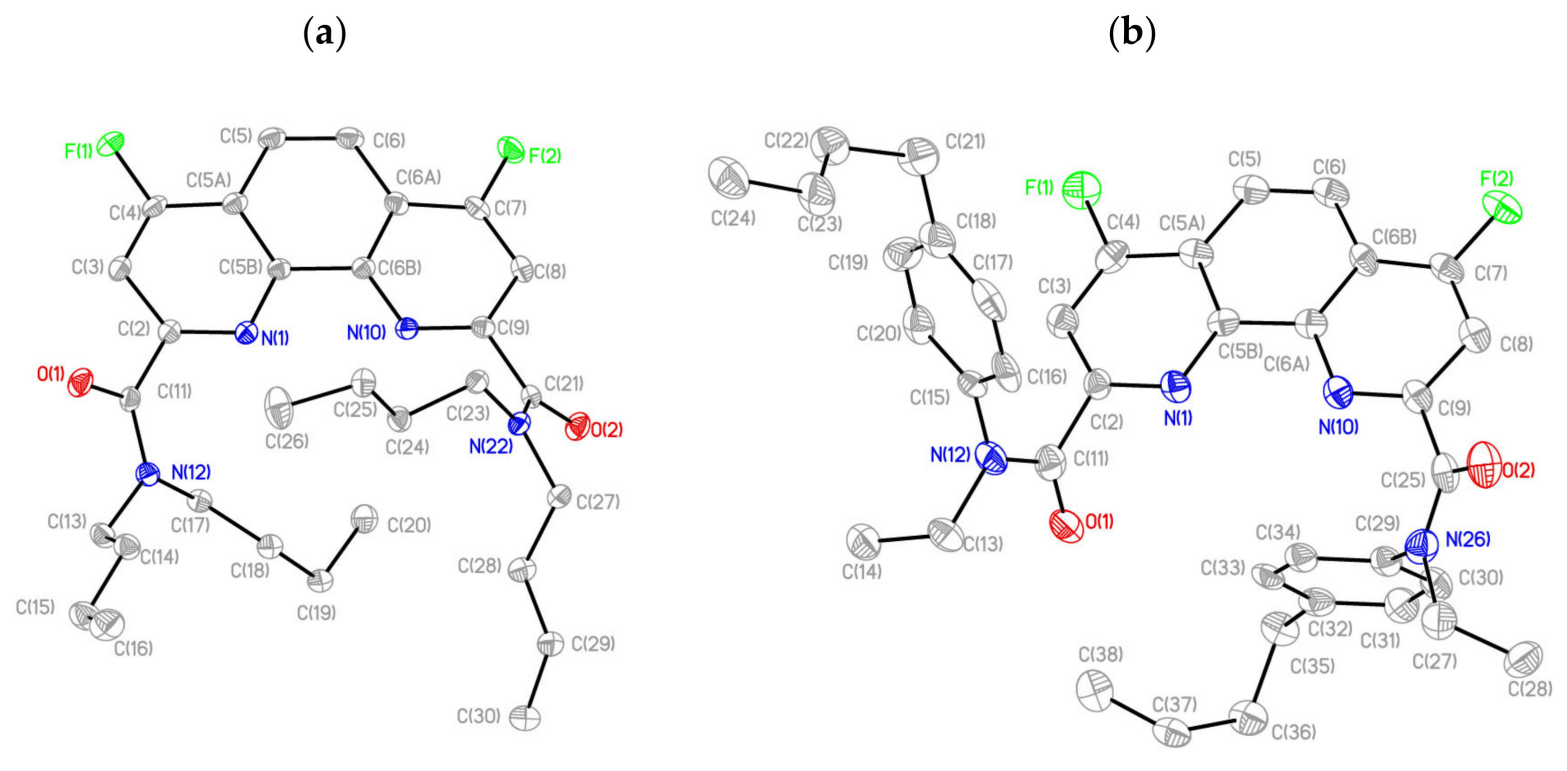
Structure of ligands **7a** (**a**) and **7c** (**b**) in representation of atoms be ellipsoids of atomic displacement parameters. The H-atoms and disordered alkyl chain in **7c** are omitted for clarity.

Detailed analytical data and detailed synthesis techniques are given in the experimental part and ESI. The structure of fluorinated ligands was unambiguously confirmed by the X-ray data on the example of compounds **7a** and **7c**. The bond lengths and angles in **7a** and **7c** are similar to each other and close to the expected values for this class of compounds. The amide groups in both ligands in crystal are almost orthogonal to phenanthroline core indicating the absence of any conjugation between these two moieties (Figure 4). In both molecules, C=O groups of amide fragments are oriented in opposite direction with pseudo torsion angles ca 180°.

**Figure 5:**
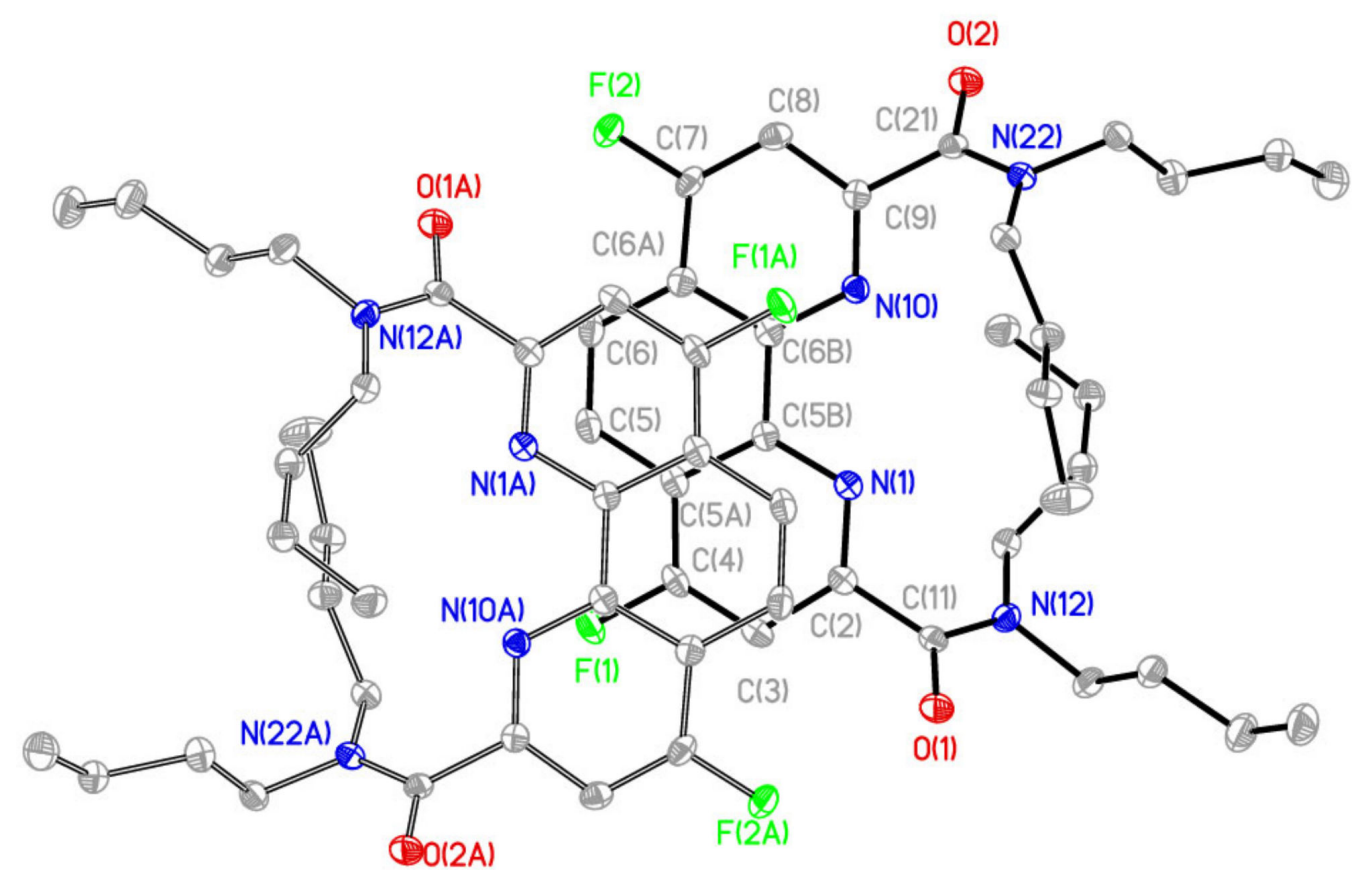
Centrosymmetric dimers in the crystal of **7a**.

In both **7a** and **7c** crystals, molecules are involved in centrosymmetric dimers by stacking interaction with interplane distance equal to 3.4 Å (Figure 5).

The structures of the newly synthesized diamides **4c** and **7a**–**d** were studied in solutions using multinuclear NMR. *n*-Bu substituted diamide **7a** and pyrrolidine derived compound **7b** demonstrates relatively fast rotation along the “Phen”–C(O) bonds at 25 °C on the NMR time scale; therefore, the spectra contain narrow signals. In their turn, diamides **4c**, **7c**, and **7d** contain aromatic substituents in the amide fragments and demonstrates slow rotation around the “Phen”–C(O) bonds. As a result, **7a**–**d** exist in solutions as mixtures of conformers, interconversions of which are strongly temperature dependent. The signals in the NMR spectra of these diamides at 25 °C are strongly broadened, but become narrow as the temperature rises to 60 °C. As an example, ^1^H NMR spectra of **7c** in benzene-d_6_ at 25 °C and 60 °C are shown in Figure 3. This phenomenon has already been studied by us earlier on the example of diamides of a similar structure [7,9]. The full assignment of the signals in the NMR spectra was done using 2D and ^19^F NMR spectroscopy methods [9].

Fluorine-containing phenanthrolinediamides **7a**–**d** are supposed to be investigated in liquid-liquid extraction experiments for the separation of *f*-elements. Within the framework of this work, we obtained a series of complex compounds of ligand **7a** with nitrates of some lanthanoids (La, Nd, Eu and Lu).

### 2.2. Preparation of Complexes with Nitrates of REE

**Figure 6:**
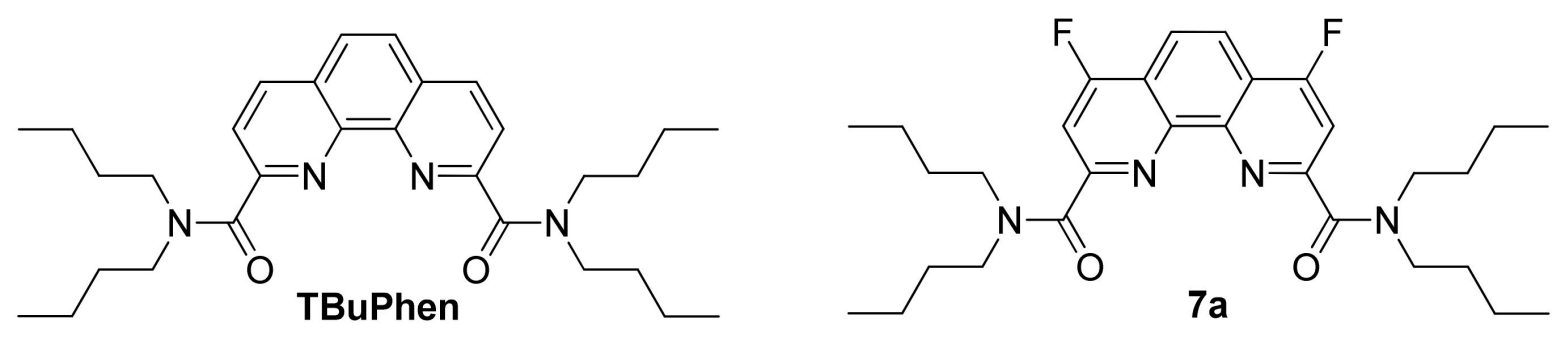
Structural formulas of **TBuPhen** and **7a** ligand.

Previously, we obtained a series of complexes of some lanthanoids with the **TBuPhen** ligand (Figure 6) which has no fluorine atoms in the structure.

**Figure 7:**
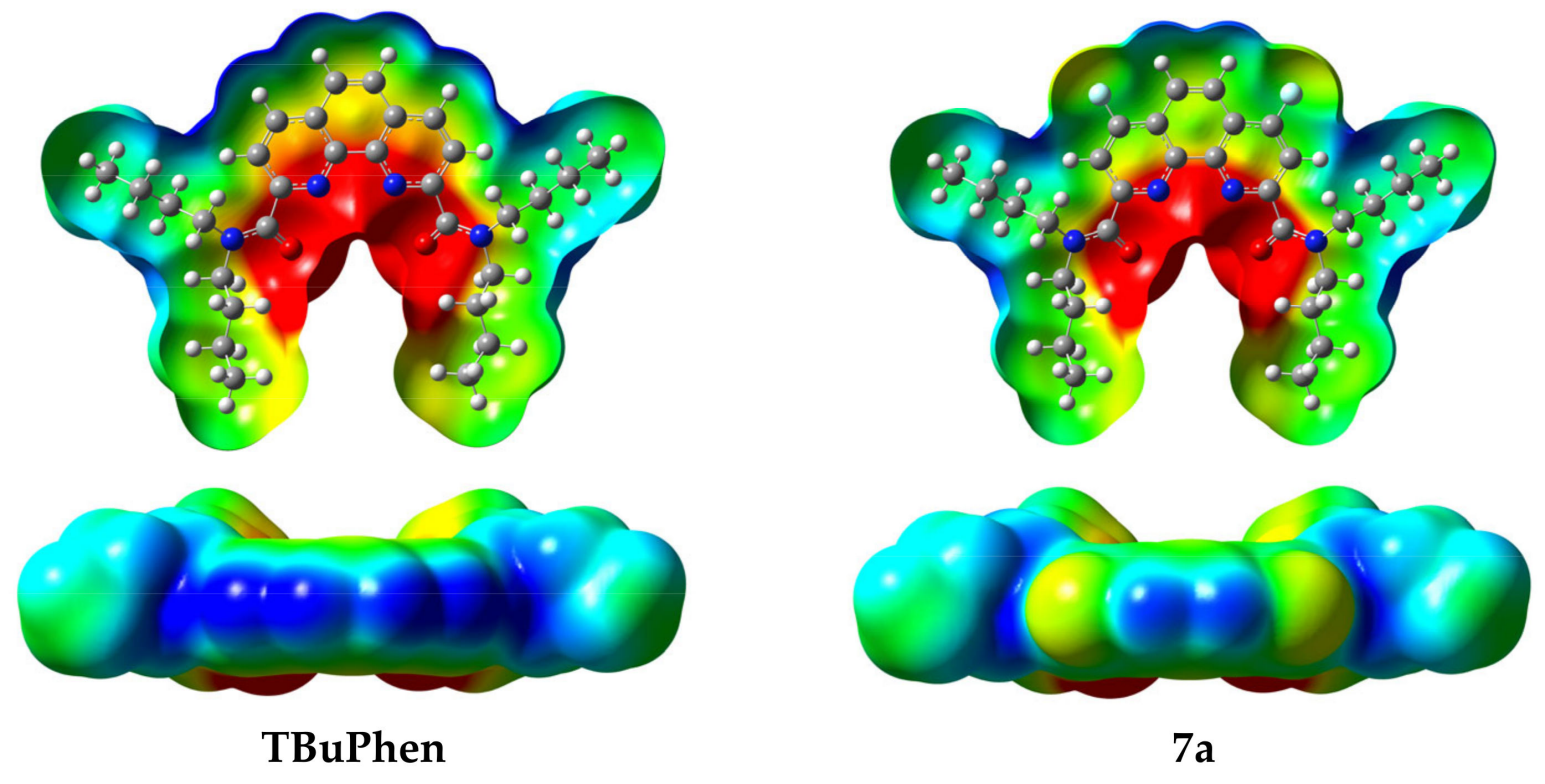
ESP maps of **TBuPhen** and **7a** in two projections.

We decided to compare electronic properties of nonfluorinated ligand **TBuPhen** and **7a**. For this aim, electrostatic potential (ESP) maps at PBE0/def2-TZVP theoretical level for both ligands in optimized geometries were calculated. ESP maps in two projections shown in Figure 7 (a single potential scale from −0.025 to +0.025 conventional units). Natural bond orbitals (NBO) charges of some atoms for optimized geometries **TBuPhen** and **7a** are given in Table 2. All quantum chemical calculations were performed using Gaussian16 [38] software package at PBE0-D3/def2-TZVP level of theory. Hybrid PBE0 [39,40] functional and Weigend def2-TZVP [41] basis set has recommended itself as a highly accurate method for modeling small organic molecules [42,43]. Grimme’s D3 dispersion correction has been used to better estimation of non-covalent interactions [44]. Tight optimization criteria and ultrafine grids were used in each calculation.

ESP maps and calculated charges at the carbonyl oxygens and phenanthroline nitrogens look very similar. However, the corresponding basicity of fluorinated and nonfluorinated derivatives can differ significantly which is very important for the formation of complexes with lanthanoids and the extraction properties of the ligands. For example, pKa of parent pyridine is 5.25. The corresponding value for 4-fluoropyridine is 4.15. pKa for **TBuPhen** and **7a** calculated in a similar manner are 0.54 and −1.22.

The synthesis of complexes of **7a** with nitrates La, Nd, Eu, and Lu was carried out by analogy with the method of obtaining **TBuPhen** complexes [45]. For detailed procedure, see the Experimental part. Their structures were investigated in solid form by IR spectroscopy and X-ray diffraction. The structure of complex compounds **7a•Ln(NO_3_)_3_** was studied by IR spectroscopy and X-ray diffraction in the solid state and by NMR spectroscopy (2D, ^1^H, ^13^C, ^19^F). In the IR spectra of the obtained complexes, there is a shift in the vibrations of the C=O band compared to the value for the ligand by 25–31 cm^−1^ (Table 3). Attention is drawn to the fact that a decrease in the atomic radius of lanthanoid leads to a decrease in the value of Δν(CO).

**Figure 8:**
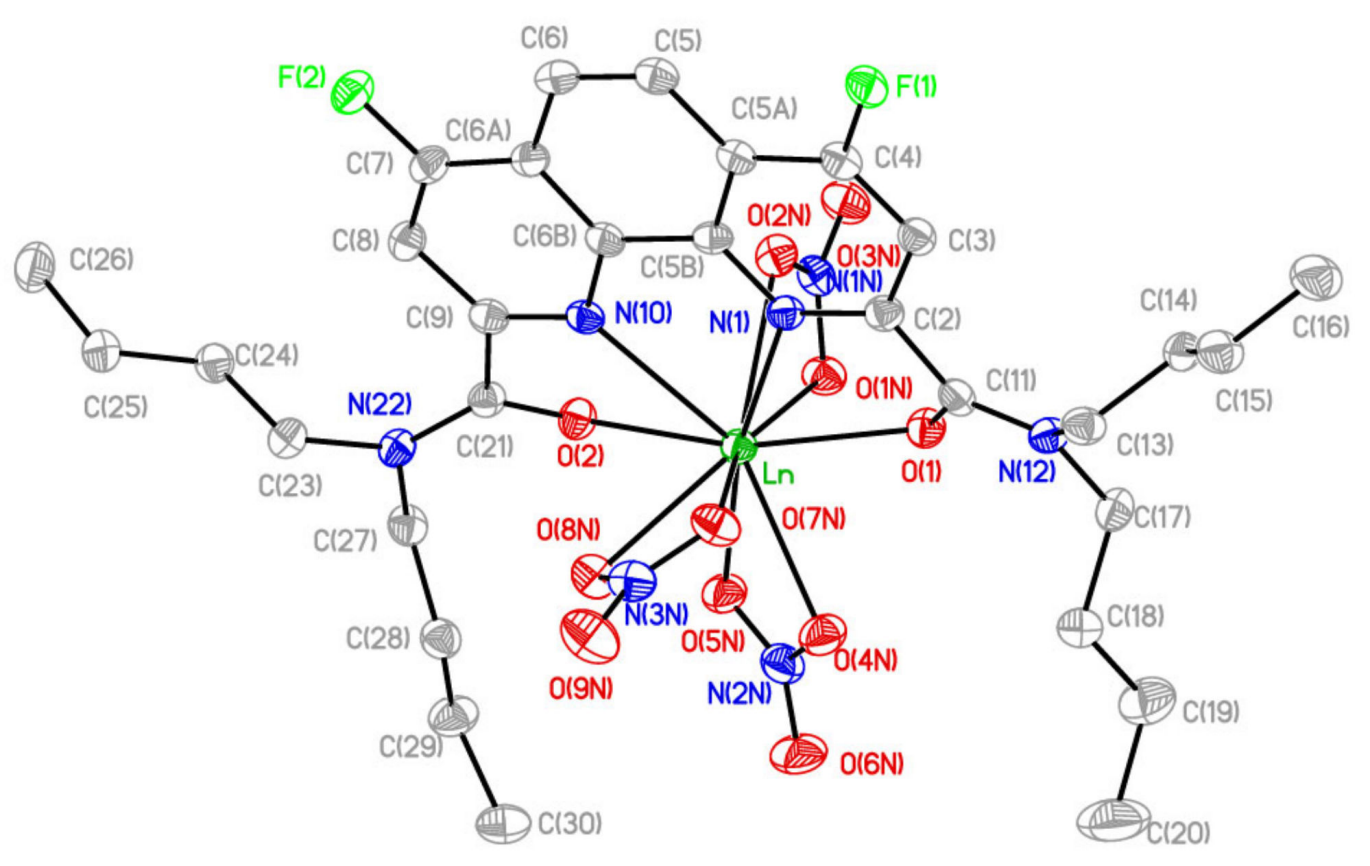
The general view of **7a•Ln(NO_3_)_3_** complexes. The atoms are shown as the ellipsoids of atomic displacement parameters (*p* = 50%) for the **7a•Nd(NO_3_)_3_** complexes.

The structure of the complexes **7a•La(NO_3_)_3_**, **7a•Nd(NO_3_)_3_**, and **7a•Eu(NO_3_)_3_** was investigated by X-ray diffraction. Complexes **7a•La(NO_3_)_3,_ 7a•Nd(NO_3_)_3_**, and **7a•Eu(NO_3_)_3_** were obtained as isostructural solvates with two acetonitrile molecules. Considering that complexes are isostructural, we will discuss hereinafter the neodymium complex **7a•Nd(NO_3_)_3_** as the most accurate one (Figure 8). The variation of Ln-X bonds in the isostructural series coincides well with the expected lanthanoid contraction [45]. The phenanthroline core acts as a tetradentate ligand. Coordination of lanthanoid cation occurs by binding to two amide oxygen atoms and two nitrogen atoms of the heterocyclic core. The coordination number of lanthanoid ions is supplemented to 10 by three bidentate nitrate anions. The coordination polyhedron of lanthanoid can be described as sphenacorona J87 according to SHAPE 2.1 [46,47].

The mutual arrangement of N-(n-Bu)_2_ groups with respect to the phenantroline core is determined by the conformation of the 5-memered LnO(1)C(11)C(2)N(1) and LnO(2)C(21)C(9)N(10) cycles. The latter are both characterized by the envelope conformation with a deviation of corresponding oxygen atom in the same direction. As a consequence, the average N-(n-Bu_2_) planes are shifted to the same direction with respect to the plane of phenanthroline ring. The deviation of Nd(1) from the plane of coordinated sites of the ligand (N(1), N(10), O(1), and O(2)) is equal to 0.41 Å. The conformation of n-Bu groups is close to all-trans.

Considering that the coordination sphere and coordination number in **7a•Ln(NO_3_)_3_** are identical to **TBuPhen•Ln(NO_3_)_3_** [45], it was interesting to estimate the extent to which the introduction of fluorine atoms to phenantroline core affects the Ln-X bonds in the complexes.

As one can see from the selected bond lengths and angles for Nd complexes with ligands **7a** and **TBuPhen** the introduction of fluorine atoms to the ligand leads on the one hand to the elongation of Nd-N bonds, but on the other hand to the similar decrease in the lengths of the Nd-O bonds (Table 4). Thus, it is reasonable to propose that overall interaction energy for particular lanthanoid ion with ligand in the case of **7a** and **TBuPhen** can be almost the same. The introduction of fluorine atoms does not lead to a significant change in the distribution of bond lengths in the phenanthroline core. Comparison of the bond lengths in ligand **7a** and the corresponding complex also shows that the change of lengths of the N-C bonds is almost negligible upon coordination. Finally, we can mention that bond lengths C-C of amide with phenanthroline is insensitive to the conjugation and is governed by the steric repulsion. Indeed, the variation of C(9)–C(21) and C(2)–C(11) in complex and pure ligand is on average 0.005 Å, while in complex amide is coplanar to phenanthroline ring and in **7a** is orthogonal to it.

**Figure 9:**
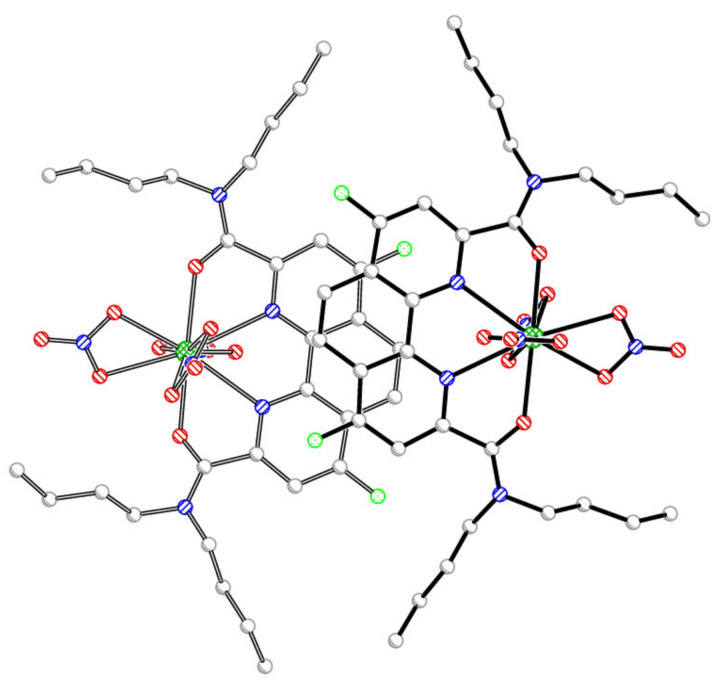
The stacking dimer in the crystal of **7a•Ln(NO_3_)_3_**.

**Figure 10:**
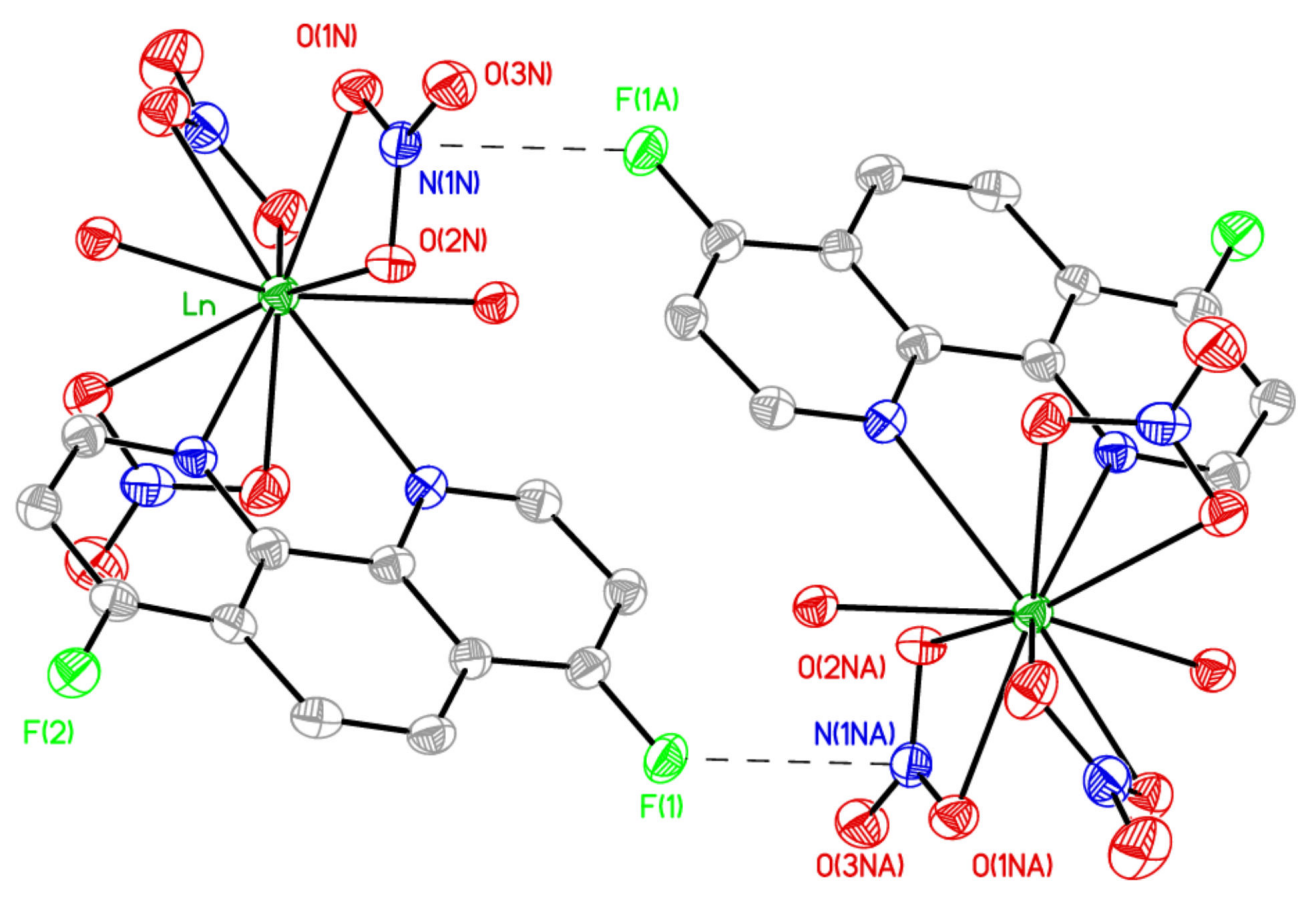
Centrosymmetric dimers assembled via F…NO_3_ interaction. The amide groups are omitted for clarity.

The analysis of crystal packing revealed that first phenanthroline is involved in, rather expected for this class of compounds, stacking interaction with rather high overlap and interplane distance equal to 3.286 Å (Figure 9). In addition to the expected for phenanthroline complexes stacking interaction, one rather unusual dimer can be found in the crystal formed by the C-F…NO_3_ interaction (Figure 10). According to the CSD search, such interatomic interaction with F…O distance shorter than sum of Van-der-Waals radii is observed in 11 crystals without disorder with the shortest F…O contact equal to 2.850 [48], which are all longer than those found in Nd (2.764(4) Å), Eu (2.770(4) Å), and La (2.785(4) Å) complexes.

Taking into account that F…NO_3_^−^ is characterized by specific orientation, i.e., F…N is perpendicular to NO_3_^−^ group and C-F…N angle is 119.3°, we can attribute this interaction to charge transfer from the fluorine to π* orbital. As far as we know, this is the first example of such type of interaction described in literature.

NMR spectroscopy also provides valuable information on the structure of the obtained complexes **7a•Ln(NO_3_)_3_** in CDCl_3_ solution. The application of 2D NMR methods makes it possible to make complete attributions in all registered spectra of the complexes. Fragmental views of ^1^H NMR spectra are shown in Figure 11. As it may be seen from Figure 11, the presence of lanthanoids strongly affects the shifts of phenanthroline protons in a multidirectional manner depending on the lanthanoid. The full spectral data are given in the experimental part and ESI (Figures S36–S59). In the future, we plan to study in detail the extraction properties of the synthesized fluorinated phenanthroline diamides.

## 3. Materials and Methods

### 3.1. Materials

Chemical reagents such as La(NO_3_)_3_·6H_2_O, Nd(NO_3_)_3_·6H_2_O, Eu(NO_3_)_3_·6H_2_O, Lu(NO_3_)_3_·xH_2_O, and other inorganic/organic reagents and solvents were of analytical grade. Water content x in lutetium nitrate was determined as x = 3. Deuterated solvents CDCl_3_ and C_6_D_6_ for NMR spectra registration were purchased from commercial sources and used without further purification. All syntheses were performed in an argon inert atmosphere. Dimethyl sulfoxide and dichloromethane were purified by distillation over calcium hydride prior to use. Acetonitrile, chloroform, and diethyl ether for the synthesis of complexes were purified according to known procedures.

### 3.2. Methods

The Log P values given in Table 1 were predicted using ACD/LogP software (Advanced Chemistry Development, Inc., Toronto, ON, Canada).

NMR spectra were recorded using standard 5 mm sample tubes on an Agilent 400-MR spectrometer (Agilent Technologies, Santa Clara, CA, USA) with operating frequencies of 400.1 MHz (^1^H), 100.6 MHz (^13^C), and 376 MHz (^19^F). The ROESY spectrum was recorded on an Agilent 400-MR spectrometer using a standard ROESYAD pulse sequence with a mixing time of 200 ms and an acquisition time of 150 ms. IR spectra in the solid state were recorded on a Nicolet iS5 FTIR spectrometer (Thermo Fisher Scientific, Waltham, MA, USA) using an internal reflectance attachment with diamond optical element attenuated total reflection (ATR) with a 45° angle of incidence. The resolution is 4 cm^−1^; the number of scans is 32 HRMS ESI mass spectra were recorded on a LCMS-IT-TOF (Shimadzu, Japan) in the negative and positive ion modes. Resolution of the instrument is >10,000 according to the manufacturer. LabSolution software (version 3.80.410, Shimadzu, Kyoto, Japan) was used to acquire and process MS data.

Single crystals of **7a** and **7c** were obtained upon slow isothermal (25 °C) recrystallization of corresponding substances from hexane, single crystals of complexes **7a•La(NO_3_)_3_**, **7a•Nd(NO_3_)_3_** and **7a•Eu(NO_3_)_3_** were obtained upon slow isothermal (25 °C) crystallization of corresponding substances from acetonitrile. The crystallographic data were collected using Quest D8 diffractometer (Bruker, Munich, Germany) equipped with a Photon-III area-detector (shutterless φ- and ω-scan technique), using Mo Ka-radiation. The intensity data were integrated by the SAINT program and corrected for absorption and decay by SADABS. Structures were solved by direct methods using SHELXT and refined against F2 using SHELXL-2018. Analysis of anisotropic displacement parameters and Fourier density synthesis revealed that in **7a•La(NO_3_)** and **7c**, the alkyl groups and NO_3_ and La ion are disordered by two positions. The refinement of two positions of carbon atoms were performed with the EADP and DFIX constraints. The positions of hydrogen atoms were calculated and refined in the riding model. Crystal data, data collection, and structure refinement details are summarized in Table S1.

### 3.3. Synthesis and Analytical Data

*4,7-difluoro-2,9-dimethyl-1,10-phenanthroline* (**6**).


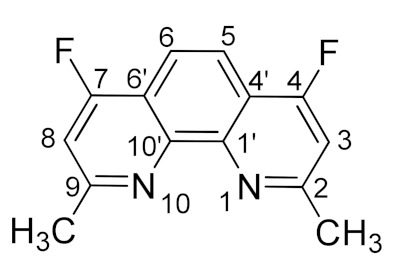


First, 2.77 g (10 mmol) of 2.9-dimethyl-4.7-dichloro-1.10-phenanthroline, 4.56 g (30 mmol) of cesium fluoride, and 100 mL of dry dimethyl sulfoxide were mixed. The reaction mixture was heated to 110 °C and stirred at this temperature for 24 h. The resulting mixture was diluted with 600 mL of dichloromethane and extracted with water (3 × 600 mL). The organic phase was evaporated on a rotary evaporator to a dry residue. Purification was carried out by hot filtration-recrystallization from a mixture of hexane-ethyl acetate in a ratio of 1:5. The resulting solution was evaporated to a dry residue. Yield 33% (805 mg). M.p. 158–161 °C (with decomposition); ^1^H NMR (CDCl_3_) δ 8.00 (s, 2H, H^5,6^) 7.22 (d, ^3^*J*_H-F_ = 10.2 Hz, 2H, H^3,8^) 2.92 (s, 6H, CH_3_); ^13^C NMR (CDCl_3_) δ 165.5 (d, ^1^*J*_C-F_ = 266.2 Hz, C^4,7^), 161.9 (d, ^3^*J*_C-F_ = 8.1 Hz, C^2,9^), 147.2–146.6 (m, C^1′,10′^), 118.2 (d, ^2^J = 13.2 Hz, C^4′,6′^), 118.1–118.0 (m, C^5,6^), 109.0 (d, ^2^*J*_C-F_ = 14.7 Hz, C^3,8^), 26.4 (d, ^4^*J*_C-F_ = 2.9 Hz, CH_3_); ^19^F NMR (CDCl_3_) δ 112.82 (d, ^3^*J*_H-F_ = 10.2 Hz); IR, ν, sm^−1^: 3039, 2954 (C-H), 1137 (C-F), 1616, 1548, 1495 (C=N, C=C arom.); ESI-HRMS (*m*/*z*): calcd for (C_14_H_10_F_2_N_2_) [M + H]^+^ 245.0885, found 245.0882.

*N^2^,N^9^-bis(4-butylphenyl)-4,7-dichloro-N^2^,N^9^-diethyl-1,10-phenanthroline-2,9-dicarboxamide* (**4c**).


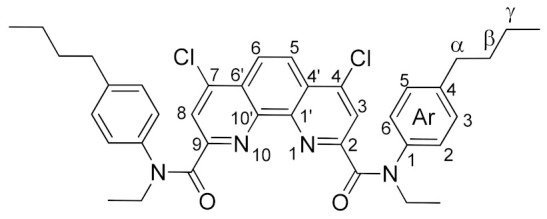


A solution of 4.43 g (25 mmol) of 4-butyl-N-ethylaniline and 3.5 mL of triethylamine (25 mmol) in 10 mL of dichloromethane was added at -10 °C with stirring to a suspension of 3.74 g (10 mmol) 4,7-dichloro-1,10-phenanthroline-2,9-dicarboxylic acid in 25 mL of dichloromethane. Then, the reaction mixture was allowed to heat up to room temperature and stirred overnight. Then, the reaction mixture was allowed to heat up to room temperature and stirred overnight. Next, the reaction mixture was diluted with 50 mL of dichloromethane, washed with water (2 × 50 mL), dried over sodium sulfate and the solvent was distilled. The residue was washed with hexane and recrystallized from a hexane/ethyl acetate mixture. After drying, the product was obtained in the form of a slightly colored powder. Yield 4.39 g (67%). M.p. 118–120 °C. ^1^H NMR (C_6_D_6_, 60 °C) δ 7.84 (s, 2H, Phen-H^3,8^), 7.62 (s, 2H, Phen-H^5,6^), 7.21 (d, *^3^J_HH_* = 7.8 Hz, 4H, Ar-H^3,5^), 6.89 (d, *^3^J_HH_* = 7.8 Hz, 4H, Ar-H^2,6^), 4.04 (q, *^3^J_HH_* = 7.1 Hz, 4H, N-CH_2_), 2.22 (t, *^3^J_HH_* = 7.7 Hz, 4H, α-CH_2_), 1.31 (t, *^3^J_HH_* = 7.1 Hz, 6H, N-CH_2_-CH_3_), 1.28–1.12 (m, 4H, β-CH_2_), 1.09–0.81 (m, 4H, γ-CH_2_), 0.65 (t, *^3^J_HH_* = 7.3 Hz, 6H, γ-CH_2_-CH_3_); ^13^C NMR (C_6_D_6_, 60 °C) δ 167.3 (C=O), 155.7 (Phen-C^1′,10′^), 145.9 (Phen-C ^2,9^), 142.6 (Phen-C^4,7^), 142.0 (Ar-C^4^), 140.6 (Ar-C^1^), 129.1 (Ar-C^2,3,5,6^), 126.6 (Phen-C^4′,6′^), 123.6 (Phen-C^3,8^), 123.3 (Phen-C^5,6^), 45.5 (N-CH_2_), 35.2 (α-CH_2_), 33.3 (β-CH_2_), 22.3 (γ-CH_2_), 13.8 (γ-CH_2_-CH_3_), 13.5 (N-CH_2_-CH_3_); IR (ν, cm^−1^) 3082, 3038, 2952, 2930, 2868 (C-H stretching vibrations), 1660, 1638 (C=O); HRMS (ESI-TOF) (*m*/*z*) [M + H]^+^ calcd for C_38_H_41_Cl_2_N_4_O_2_^+^ 655.2601, found 655.2598.

#### 3.3.1. General Method for Synthesis of 4,7-Difluorosubstituted 1,10-Phenanthroline-2,9-dicarboxamides

Tetramethylammonium chloride (0.23 mmol) and 1,4,7,10,13,16-hexaoxacyclooctadecane (0.25 mmol) in an argon atmosphere were placed in a three-necked flask equipped with a stirrer and a thermometer. Then, dry dimethyl sulfoxide (30 mL) and 4,7-dichloro-1,10-phenanthroline-2,9-dicarboxylic acid (2.0 mmol) were added. The mixture was heated to 80 °C and stirred until the reaction mixture became homogeneous. After that, pre-hardened cesium fluoride (20 mmol) was added and stirred at the same temperature for 4 h. Upon completion of the reaction, the mixture was poured into ice, then extracted in 3 portions of 50 mL of methylene chloride. Dried over sodium sulfate, concentrated, and purified by column chromatography.

*N^2^,N^2^,N^9^,N^9^-tetrabutyl-4,7-difluoro-1,10-phenanthroline-2,9-dicarboxamide***(7a)**.


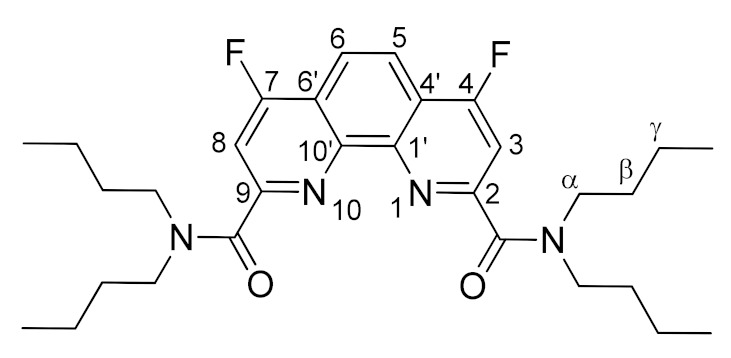


The reaction was carried out in accordance with the general procedure. The product was purified by column chromatography using 0–10% acetone in hexane. The product was obtained in yield of 88% (0.93 g), in the form of a white powder. M.p. 101–103 °C, R_f_ = 0.3 (hexane:acetone 2:1); ^1^H NMR (CDCl_3_) δ 8.17 (s, 2H, Phen-H^5,6^), 7.74 (d, *^3^J_HF_* = 9.8 Hz, 2H, Phen-H^3,8^), 3.70–3.50 (m, 8H, N-CH_2_), 1.82–1.63 (m, 8H, β-CH_2_), 1.44 (h, *^3^J_HH_* = 7.3 Hz, 4H, γ-CH_2_), 1.08 (h, *^3^J_HH_* = 7.3 Hz, 4H, γ-CH_2_), 0.99 (t, *^3^J_HH_* = 7.3 Hz, 6H, CH_3_), 0.67 (t, *^3^J_HH_* = 7.3 Hz, 6H, CH_3_); ^13^C NMR (CDCl_3_) δ 167.4 (d, *^5^J_CF_* = 3.4 Hz, C=O), 165.9 (d, *J_CF_* = 267.7 Hz, Phen-C^4,7^), 156.8 (d, *^3^J_CF_* = 7.4 Hz, Phen-C^2,9^), 146.2 (Phen-C^1′,10′^), 120.2 (d, *^2^J_CF_* = 13.0 Hz, Phen-C^4′,6′^), 119.8 (d, *^3^J_CF_* = 6.2 Hz, Phen-C^5,6^), 109.3 (d, ^2^*J_CF_* = 16.6 Hz, Phen-C ^3,8^), 49.2 (N-CH_2_), 46.8 (N-CH_2_), 31.3 (β-CH_2_), 29.9 (β-CH_2_), 20.5 (γ-CH_2_), 20.0 (γ-CH_2_), 14.1 (CH_3_), 13.70 (CH_3_); ^19^F NMR (CDCl_3_) δ-109.98 (d, *^3^J_FH_* = 9.8 Hz); IR (ν, cm^−1^) 3052, 2956, 2932, 2869 (C-H stretching vibrations), 1634 (C=O); HRMS (ESI-TOF) (*m*/*z*) [M + H]^+^ calcd for C_30_H_41_F_2_N_4_O_2_^+^ 527.3192, found 527.3179.

*N^2^,N^9^-bis(pyrrolidine)-4,7-difluoro-N^2^,N^9^-diethyl-1,10-phenanthroline-2,9-dicarboxamide* (**7b**).


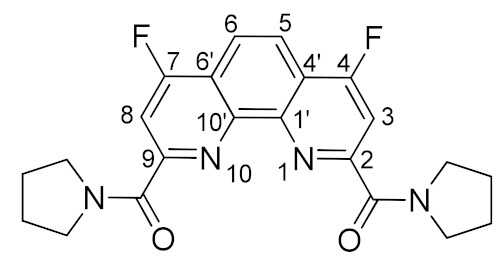


The reaction was carried out in accordance with the general procedure. The product was washed with acetone. The product was obtained in yield of 80% (0.66 g), in the form of a yellowish powder. M.p. 295–267 °C; R_f_ = 0.5 (hexane:acetone 1:1); ^1^H NMR (CDCl_3_) δ 8.21 (s, 2H, Phen-H^5,6^), 8.00 (d, *^3^J_HF_* = 9.9 Hz, 2H, Phen-H^3,8^), 4.19–4.10 (m, 4H, N-CH_2_), 3.81–3.69 (m, 4H, N-CH_2_), 2.03–1.92 (m, 8H, CH_2_-CH_2_); ^13^C NMR (CDCl_3_) δ 165.9 (d, *J_CF_* = 268.0 Hz, Phen-C^4,7^), 164.9 (d, *^5^J_CF_* = 3.4 Hz, C=O), 156.4 (d, *^3^J_CF_* = 7.5 Hz, Phen-C ^2,9^), 146.2 (Phen-C^1′,10′^), 120.6 (d, ^2^*J_CF_* = 13.1 Hz, Phen-C^4′,6′^), 120.1 (d, *^3^J_CF_* = 6.0 Hz, Phen-C^4′,6′^), 109.7 (d, *^2^J_CF_* = 17.0 Hz, Phen-C^3,8^), 49.3 (Pyrr-CH_2_), 47.4 (Pyrr-CH_2_), 26.9 (Pyrr-CH_2_), 24.2 (Pyrr-CH_2_); ^19^F NMR (CDCl_3_) δ-109.32 (d, *^3^J_FH_* = 9.9 Hz); IR (ν, cm^−1^) 3062, 2967, 2950, 2881 (C-H stretching vibrations), 1628, 1618 (C=O); HRMS (ESI-TOF) (*m*/*z*) [M + H]^+^ calcd for C_22_H_21_F_2_N_4_O_2_^+^ 411.1627, found 411.1621.

*N^2^,N^9^-bis(4-butylphenyl)-4,7-difluoro-N^2^,N^9^-diethyl-1,10-phenanthroline-2,9-dicarboxamide* (**7c**).


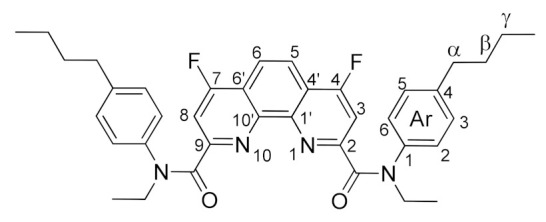


The reaction was carried out in accordance with the general procedure. The product was purified by column chromatography using 0–20% ethyl acetate in hexane. The product was obtained in yield of 74.4% (0.93 g), in the form of a white powder. M.p. 103–105 °C; R_f_ = 0.3 (hexane: ethyl acetate 3:2); ^1^H NMR (C_6_D_6_, 60 °C) δ 7.41 (d, *^3^J_HF_* = 9.8 Hz, 2H, Phen-H^3,8^), 7.37 (s, 2H, Phen-H^5,6^), 7.21 (d, *^3^J_HH_* = 7.9 Hz, 4H, Ar-H^3,5^), 6.90 (d, *^3^J_HH_* = 7.9 Hz, 4H, Ar-H^2,6^), 4.04 (q, *^3^J_HH_* = 7.1 Hz, 4H, N-CH_2_), 2.23 (t, *^3^J_HH_* = 7.7 Hz, 4H, α-CH_2_), 1.30 (t, *^3^J_HH_* = 7.1 Hz, 6H, N-CH_2_-CH_3_), 1.28–1.17 (m, 4H, β-CH_2_), 1.04–0.89 (m, 4H, γ-CH_2_), 0.66 (t, *^3^ J_HH_* = 7.3 Hz, 6H, γ-CH_2_-CH_3_); ^13^C NMR (C_6_D_6_, 60 °C) δ 167.4 (d, *^5^J_CF_* = 3.5 Hz, C=O), 165.5 (d, *J_CF_* = 266.2 Hz, Phen-C^4,7^), 157.5 (d, *^3^J_CF_* = 7.0 Hz, Phen-C^1′,10′^), 146.7 (Phen-C ^2,9^), 142.0 (Ar-C^4^), 140.7 (Ar-C^1^), 129.1 (Ar-C^2,3,5,6^), 119.5 (d, *^2^J_CF_* = 12.9 Hz, Phen-C^4′,6′^), 119.1 (d, *^3^J_CF_* = 6.1 Hz, Phen-C^5,6^), 108.6 (d, ^2^*J_CF_* = 16.4 Hz, Phen-C^3,8^), 45.5 (N-CH_2_), 35.2 (α-CH_2_), 33.3 (β-CH_2_), 22.3 (γ-CH_2_), 13.8 (γ-CH_2_-CH_3_), 13.5 (N-CH_2_-CH_3_); ^19^F NMR (C_6_D_6_, 60 °C) δ-112.75 (d, *^3^J_FH_* = 9.8 Hz); IR (ν, cm^−1^) 3070, 2955, 2929, 2870 (C-H stretching vibrations), 1655, 1643 (C=O); HRMS (ESI-TOF) (*m*/*z*) [M + H]^+^ calcd for C_38_H_41_F_2_N_4_O_2_^+^ 623.3193, found 623.3196.

*N^2^,N^9^-bis(4-hexylpheny)-4,7-difluoro-N^2^,N^9^-diethyl-1,10-phenanthroline-2,9-dicarboxamide* (**7d**).


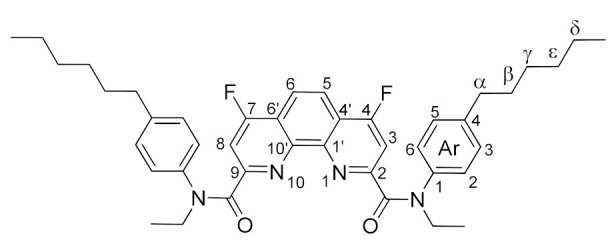


The reaction was carried out in accordance with the general procedure. The product was purified by column chromatography using 0–20% ethyl acetate in hexane. The product was obtained in yield of 78% (1.06 g), in the form of a white powder. M.p. 108–110 °C; R_f_ = 0.28 (hexane: ethyl acetate 3:2); ^1^H NMR (C_6_D_6_, 60 °C) δ 7.41 (d, *^3^J_HF_* = 10.3 Hz, 2H, Phen-H^3,8^), 7.38 (s, 2H, Phen-H^5,6^), 7.22 (d, *^3^J_HH_* = 7.9 Hz, 4H, Ar-H^3,5^), 6.92 (d, *^3^J_HH_* = 7.9 Hz, 4H, Ar-H^2,6^), 4.04 (q, *^3^J_HH_* = 7.1 Hz, 4H, N-CH_2_), 2.25 (t, *^3^J_HH_* = 7.7 Hz, 4H, α-CH_2_), 1.41–1.20 (m, 10H, N-CH_2_-CH_3_, β-CH_2_), 1.17–0.89 (m, 12H, γ-CH_2_, δ-CH_2_, ε -CH_2_), 0.78 (t, *^3^J_HH_* = 6.8 Hz, 6H, γ-CH_2_-CH_3_); ^13^C NMR (C_6_D_6_, 60 °C) δ 167.4 (d, *^5^J_CF_* = 3.5 Hz, C=O), 165.5 (d, *J_CF_* = 266.1 Hz, Phen-C ^4,7^), 157.5 (d, *^3^J_CF_* = 7.0 Hz, Phen-C^2,9^), 146.8 (Phen-C^1′,10′^), 142.1 (Ar-C^4^), 140.7 (Ar-C^1^), 129.1 (Ar-C^2,3,5,6^), 119.5 (d, *^2^J_CF_* = 12.8 Hz, Phen-C^4′,6′^), 119.1 (d, *^3^J_CF_* = 6.1 Hz, Phen-C^5,6^), 108.6 (d, ^2^*J_CF_* = 16.4 Hz, Phen-C^3,8^), 45.6 (N-CH_2_), 35.6 (α-CH_2_), 31.9 (ε -CH_2_), 31.1 (β-CH_2_), 29.0 (γ-CH_2_), 22.8 (δ-CH_2_), 14.0 (γ-CH_2_-CH_3_), 13.5 (N-CH_2_-CH_3_); ^19^F NMR (C_6_D_6_, 60 °C) δ-112.74 (d, *J* = 10.3 Hz); IR (ν, cm^−1^) 3051, 2958, 2930, 2856 (C-H stretching vibrations), 1663, 1640 (C=O); HRMS (ESI-TOF) (*m*/*z*) [M + H]^+^ calcd for C_42_H_49_F_2_N_4_O_2_^+^ 679.3819, found 679.3806.

#### 3.3.2. General Method for Synthesis of Complexes 7a•Ln(NO_3_)_3_

To a solution of *N*^2^,*N*^2^,*N*^9^,*N*^9^-tetrabutyl-4,7-difluoro-1,10-phenanthroline-2,9-dicarboxamide (0.1 mmol) in acetonitrile (1 mL) was added by dropwise a solution of lanthanoid nitrate (0.1 mmol) also in acetonitrile (1 mL). After, the reaction mixture was concentrated in vacuum to 1/10 of the initial volume, and then treated with diethyl ether (2 mL). The resulting complex was filtered and washed with ether, dried in air.

*N*^2^,*N*^2^,*N^9^*,*N^9^-tetrabutyl-4,7-difluoro-1,10-phenanthroline-2,9-dicarboxamide lanthanum trinitrate***7a•La(NO_3_)_3_**. Yield 83.1% (70.6 mg). White powder. m.p. (with decomposition); ^1^H NMR (CDCl_3_) δ 8.33 (s, 2H, Phen-H^5,6^), 7.77 (d, *^3^J_HF_* = 8.8 Hz, 2H, Phen-H^3,8^), 3.74–3.63 (m, 8H, N-CH_2_),1.94–1.71 (m, 8H, β-CH_2_), 1.43 (p, *^3^J_HH_*= 7.7 Hz, 8H, γ-CH_2_),1.04–0.94 (m, 12H, CH_3_); ^13^C NMR (CDCl_3_) δ 167.5 (d, *^5^J_CF_* = 2.5 Hz, C=O), 166.1 (d, *J_CF_* = 271.2 Hz, Phen-C^4,7^), 152.8 (d, *^3^J_CF_* = 8.3 Hz, Phen-C^2,9^), 146.8 (d, *^3^J_CF_* = 5.0 Hz, Phen-C^1′,10′^), 121.6 (d, ^3^*J_CF_* = 2.7 Hz, Phen-C^5,6^), 121.4 (d, ^2^*J_CF_* = 14.4 Hz, Phen-C^4′,6′^), 110.2 (d, ^2^*J_CF_* = 19.3 Hz, Phen-C^3,8^), 50.4 (N-CH_2_), 49.1 (N-CH_2_), 30.8 (β-CH_2_), 28.9 (β-CH_2_), 20.5 (γ-CH_2_), 20.1 (γ-CH_2_), 13.9 (CH_3_), 13.7 (CH_3_); ^19^F NMR (CDCl_3_) δ-100.31 (d, *^3^J_HF_* = 8.8 Hz); IR (ν, cm^−1^) 3074, 2957, 2934, 2873 (C-H stretching vibrations), 1603 (C=O); HRMS (ESI-TOF) (*m*/*z*) [M + H]^+^ calcd for C_30_H_40_F_2_LaN_6_O_8_^+^ 789.1934, found 789.1927.

*N^2^,N^2^,N^9^,N^9^-tetrabutyl-4,7-difluoro-1,10-phenanthroline-2,9-dicarboxamide neodymium trinitrate***7a•Nd(NO_3_)_3_**. Yield 91.0% (78.3 mg). Light blue powder. m.p. 237 °C (with decomposition); ^1^H NMR (CDCl_3_) δ 9.70 (d, *^3^J_HF_* = 8.4 Hz, 2H, Phen-H^3,8^), 9.50 (s, 2H, Phen-H^5,6^), 6.33 (m, 4H, N-CH_2_), 4.64 (m, 4H, N-CH_2_), 3.10 (m, 4H, β-CH_2_), 2.47 (s, 4H, β-CH_2_), 2.31 (s, 4H, γ-CH_2_), 1.59 (m, 4H, γ-CH_2_), 1.41 (t, *^3^J_HH_* = 7.1 Hz, 6H, CH_3_), 1.08 (t, *^3^J_HH_* = 7.1 Hz, 6H, CH_3_); ^13^C NMR (CDCl_3_) δ 175.5 (C=O), 163.1 (d, *J_CF_* = 276.5 Hz, Phen-C^4,7^), 161.3 (d, *^3^J_CF_* = 7.1 Hz, Phen-C^2,9^), 158.4 (Phen-C^1′,10′^), 136.4 (d, ^2^*J_CF_* = 11.8 Hz, Phen-C^4′,6′^), 131.6 (d, ^2^*J_CF_* = 18.8 Hz, Phen-C^3,8^), 123.9 (Phen-C^5,6^), 52.9 (N-CH_2_), 52.5 (N-CH_2_), 31.2 (β-CH_2_), 30.4 (β-CH_2_), 21.4 (γ-CH_2_), 20.3 (γ-CH_2_), 14.4 (CH_3_), 13.8 (CH_3_); ^19^F NMR (CDCl_3_) δ-99.90 (d, *^3^J_FH_* = 8.4 Hz), -147.79; IR (ν, cm^−1^) 3078, 2957, 2934, 2873 (C-H stretching vibrations), 1604 (C=O); HRMS (ESI-TOF) (*m*/*z*) [M + H]^+^ calcd for C_30_H_40_F_2_N_6_NdO_8_^+^ 792.1947, found 792.1944.

*N*^2^,*N*^2^,*N*^9^,*N^9^-tetrabutyl-4,7-difluoro-1,10-phenanthroline-2,9-dicarboxamide europium trinitrate***7a•Eu(NO_3_)_3_**. Yield 96.8% (83.3 mg). White powder. m.p. 221 °C (with decomposition); ^1^H NMR (CDCl_3_) δ 6.45 (s, 2H, Phen-H^5,6^), 5.88 (d, *^3^J_HF_* = 8.9 Hz, 2H, Phen-H^3,8^), 3.48–3.23 (m, 4H, N-CH_2_), 2.22–2.02 (m, 4H, N-CH_2_), 1.69 (m, 4H, β-CH_2_), 1.60–1.48 (m, 4H, β-CH_2_), 1.09 (t, *^3^J_HH_* = 7.1 Hz, 6H, CH_3_), 1.01–0.79 (m, 8H, γ-CH_2_), 0.69 (t, *^3^J_HH_* = 7.1 Hz, 6H, CH_3_); ^13^C NMR (CDCl_3_) δ 176.8 (d, *J_CF_* = 271.8 Hz, Phen-C^4,7^), 175.4, 151.0 (d, *J* = 7.1 Hz), 138.7, 123.4 (d, *J* = 8.8 Hz), 118.0 (d, *^3^J_CF_* = 2.6 Hz, Phen-C^5,6^), 94.4 (d, *J* = 14.6 Hz), 72.3 (d, *^2^J_CF_* = 18.7 Hz, Phen-C^3,8^), 47.8 (N-CH_2_), 47.3 (N-CH_2_), 31.3 (β-CH_2_), 27.8 (β-CH_2_), 19.9 (γ-CH_2_), 19.7 (γ-CH_2_), 13.8 (CH_3_), 13.4 (CH_3_); ^19^F NMR (CDCl_3_) δ-100.33 (d, *^3^J_FH_* = 8.9 Hz); IR (ν, cm^−1^) 3079, 2957, 2934, 2873 (C-H stretching vibrations), 1606 (C=O); HRMS (ESI-TOF) (*m*/*z*) [M + H]^+^ calcd for C_30_H_40_EuF_2_N_6_O_8_^+^ 803.2082, found 803.2103.

*N^2^,N^2^,N^9^,N^9^-tetrabutyl-4,7-difluoro-1,10-phenanthroline-2,9-dicarboxamide lutetium trinitrate***7a•Lu(NO_3_)_3_.** Yield 77.6% (69.1 mg). White powder. m.p. 138–141 °C, T_decomp._ 205 °C; ^1^H NMR (CDCl_3_) δ 8.25 (s, 2H, Phen-H^5,6^), 7.80 (d, *^3^J_HF_* = 9.0 Hz, 2H, Phen-H^3,8^), 3.72 (m, 8H, N-CH_2_), 1.97 (m, 4H, β-CH_2_), 1.80 (m, 4H, β-CH_2_), 1.48 (m, 8H, γ-CH_2_), 1.08 (t, *^3^J_HH_* = 7.3 Hz, 6H, CH_3_), 1.01 (t, *^3^J_HH_* = 7.3 Hz, 6H, CH_3_); ^13^C NMR (CDCl_3_) δ 167.4 (d, *^5^J_CF_* = 1.9 Hz, C=O), 166.7 (d, *J_CF_* = 277.8 Hz, Phen-C^4,7^), 150.9 (d, *^3^J_CF_* = 7.6 Hz, Phen-C ^1′,10′^, Phen-C ^1′,10′^), 145.4 (d, *^3^J_CF_* = 9.1 Hz, Phen-C^2,9^), 121.9 (Phen-C^5,6^), 121.3 (d, *^2^J_CF_* = 14.7 Hz, Phen-C^4′,6′^), 111.5 (d, *^2^J_CF_* = 20.5 Hz, Phen-C^3,8^), 50.8 (N-CH_2_), 50.3 (N-CH_2_), 30.6 (β-CH_2_), 28.4 (β-CH_2_), 20.4 (γ-CH_2_), 20.1 (γ-CH_2_), 13.8 (CH_3_), 13.7 (CH_3_); ^19^F NMR (CDCl_3_) δ-97.82 (d, *^3^J_FH_* = 9.0 Hz); IR (ν, cm^−1^) 3079, 2963, 2935, 2874 (C-H stretching vibrations), 1609 (C=O); HRMS (ESI-TOF) (*m*/*z*) [M + H]^+^ calcd for C_30_H_40_F_2_LuN_6_O_8_^+^ 825.2278, found 825.2254.

## 4. Conclusions

Thus, nucleophilic substitution of chlorine atoms was found to be effective for obtaining diamides of 1,10-phenanthroline-2,9-dicarboxylic acid containing fluorine atoms at positions 4 and 7. New fluorinated phenanthrolinediamides **7a**–**d** were synthesized in high yield using DMSO as a solvent and cesium fluoride as a fluorinating agent. The method permits preparation of both N,N,N′,N′-tetraalkyl and N,N′-dialkyl-N,N′-diaryl diamides. The structure of all the obtained ligands was investigated by spectral methods, including 2D and ^19^F NMR spectroscopy and X-ray for ligands **7a** and **7c**. A set of complex compounds with nitrates La, Nd, Eu, and Lu was prepared using ligand **7a** as a model. The structure of these complexes has been studied both in solutions and in the solid state. X-ray diffraction analysis of the complexes showed that in the case of La, Nd, and Eu nitrates, these complexes are isostructural, the metal atom is associated with 4 coordination centers of the ligand and three bidentate nitrate groups, reaching a coordination number of 10. The exact structure of the lutetium complex may differ from the complexes with light lanthanoids. We plan to study the features of the structure of this complex in detail.

## Figures and Tables

**Figure 11:**
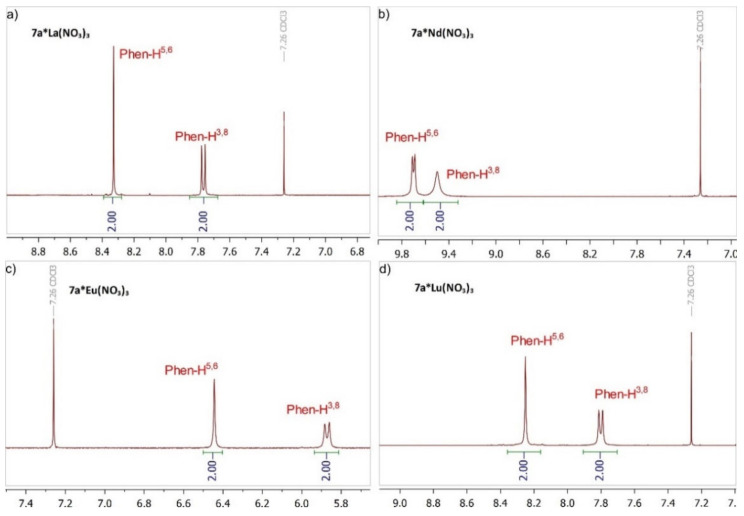
Fragmental views of ^1^H NMR spectra of complexes **7a•Ln(NO_3_)_3_** in CDCl_3_: (**a**) **7a•La(NO_3_)_3_**, (**b**) **7a•Nd(NO_3_)_3_**, (**c**) **7a•Eu(NO_3_)_3_**, (**d**) **7a•Lu(NO_3_)_3_**.

**Table 1:**
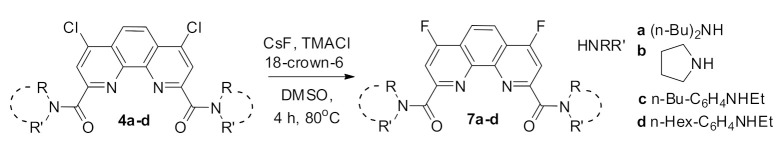
Yields and some properties of difluoro ligands **7a**–**d**.

Code	Yield, %	T_m.p._ °C	ν (CO), cm^−1^	NMR ^19^F, δ, ppm	Log *p* *
**7a**	88	101–103	1634	−110.0	5.72
**7b**	80	265–267	1628, 1618	−109.3	0.22
**7c**	74	103–105	1655, 1643	−112.8	7.83
**7d**	78	108–110	1663, 1640	−112.7	10.49

* The Log *p* values given in Table 1 were predicted using ACD/LogP software.

**Table 2:** NBO charges of N_Phen_ and O_amide_ of **TBuPhen** and **7a**.

Ligand	N_phen_	O_amide_
**TBuPhen**	−0.301/−0.305	−0.586/−0.581
**7a**	−0.310/−0.313	−0.582/−0.577

**Table 3:** Characteristic shifts of the C=O band in complexes.

	7a	7a•La(NO_3_)_3_	7a•Nd(NO_3_)_3_	7a•Eu(NO_3_)_3_	7a•Lu(NO_3_)_3_
ν(CO), cm^−1^	1634	1603	1604	1606	1609
Δν(CO)	-	31	30	28	25

**Table 4:** Selected bond lengths and angles for Nd complex with **7a** and **TBuPhen** ligands.

	7a•Nd(NO_3_)_3_	TBuPhen•Nd(NO_3_)_3_	7a
Nd(1)–N(1)	2.672(3)	2.643(2)	
Nd(1)–N(10)	2.631(3)	2.652(2)	
Nd(1)–O(1)	2.428(2)	2.434(1)	
Nd(1)–O(2)	2.415(2)	2.438(1)	
C(9)–C(21)	1.526(5)	1.519(4)	1.515(2)
C(2)–C(11)	1.514(4)	1.516(4)	1.511(2)
N(1)–C(2)	1.332(4)	1.335(2)	1.324(2)
C(2)–C(3)	1.411(4)	1.403(2)	1.411(2)
C(3)–C(4)	1.354(4)	1.376(2)	1.354(2)
C(4)–C(5A)	1.397(4)	1.403(2)	1.397(2)
tN(1)C(1)C(11)O(1)	18.9(1)	25.07(17)	131.8(1)
N(10)C(9)C(21)O(2)	28.0(1)	22.10(18)	136.3(1)

## Data Availability

Not applicable.

## Supplementary Materials

The following supporting information can be downloaded at: https://www.mdpi.com/article/10.3390/molecules27154705/s1, Figures S1–S59: copies of all ^1^H, ^13^C, ^19^F, 2D NMR, and IR spectra; Table S1: crystallographic data for **7a**, **7c**, **7a•La(NO_3_)_3_**, **7a•Nd(NO_3_)_3_**, **7a**•**Eu(NO_3_)_3_**.

## Sample Availability

Samples of the compounds are not available from the authors.
