# Supplementary material for: First Example of Fluorinated Phenanthroline Diamides: Synthesis, Structural Study, and Complexation with Lanthanoids
Source: Molecules. 2022 Jul 23;27(15):4705. doi: 10.3390/molecules27154705 (PMC9330590; doi:10.3390/molecules27154705)
Supplement: Supplementary file 1 [file molecules-27-04705-s001.zip › molecules-1832371-supplementary.pdf]

Article

First example of fluorinated phenanthroline diamides for complexation with lanthanides. Synthesis and structural study

Nane A. Avagyan [1], Pavel S. Lemport [1], Konstantin A. Lyssenko [1], Alexey O. Gudovannyy [1], Vitaly A. Roznyatovsky [1], Valentine S. Petrov [1], Mikhail F. Vokuev [1], Yuri A. Ustynyuk [1], Valentine G. Nenajdenko[1*]

[1] Department of Chemistry, Lomonosov Moscow State University, Leninskie gory 1 bld. 3, Moscow, Russia; nenajdenko@gmail.com (V.N.)

* Correspondence: nenajdenko@gmail.com (V.N.)

Supplementary Materials

Table of contents

1. NMR and IR spectra of synthesized compounds……………………………………………S2-S60
2. X-ray analysis data………………………………………………………………………….S61

1. NMR and IR spectra of synthesized compounds

N2,N9-bis(4-butylphenyl)-4,7-dichloro-N2,N9-diethyl-1,10- phenanthroline -2,9- dicarboxamide (4c)

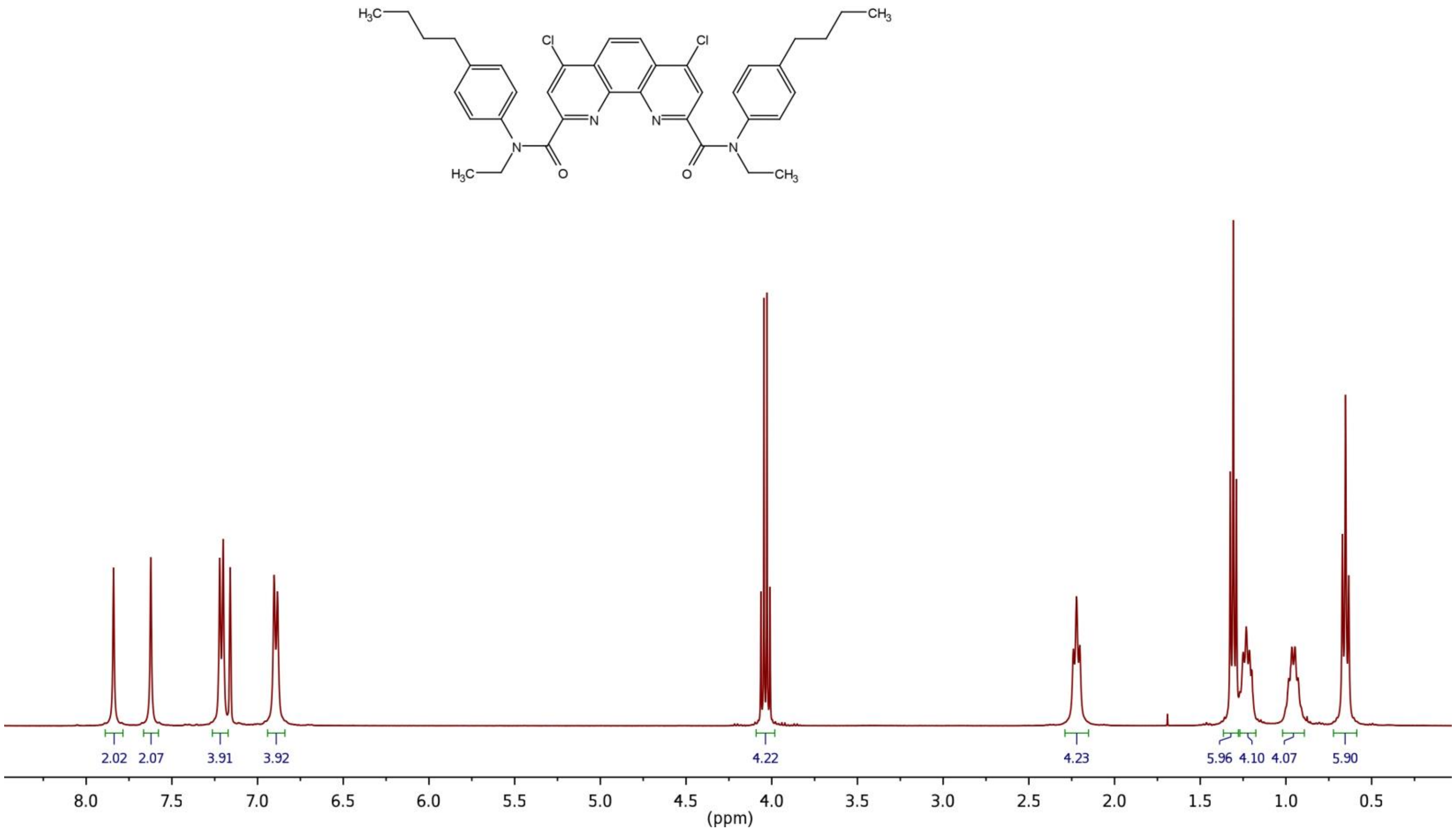

Figure S1. ^{1}H NMR spectrum in C_6D_6 at 60ºC

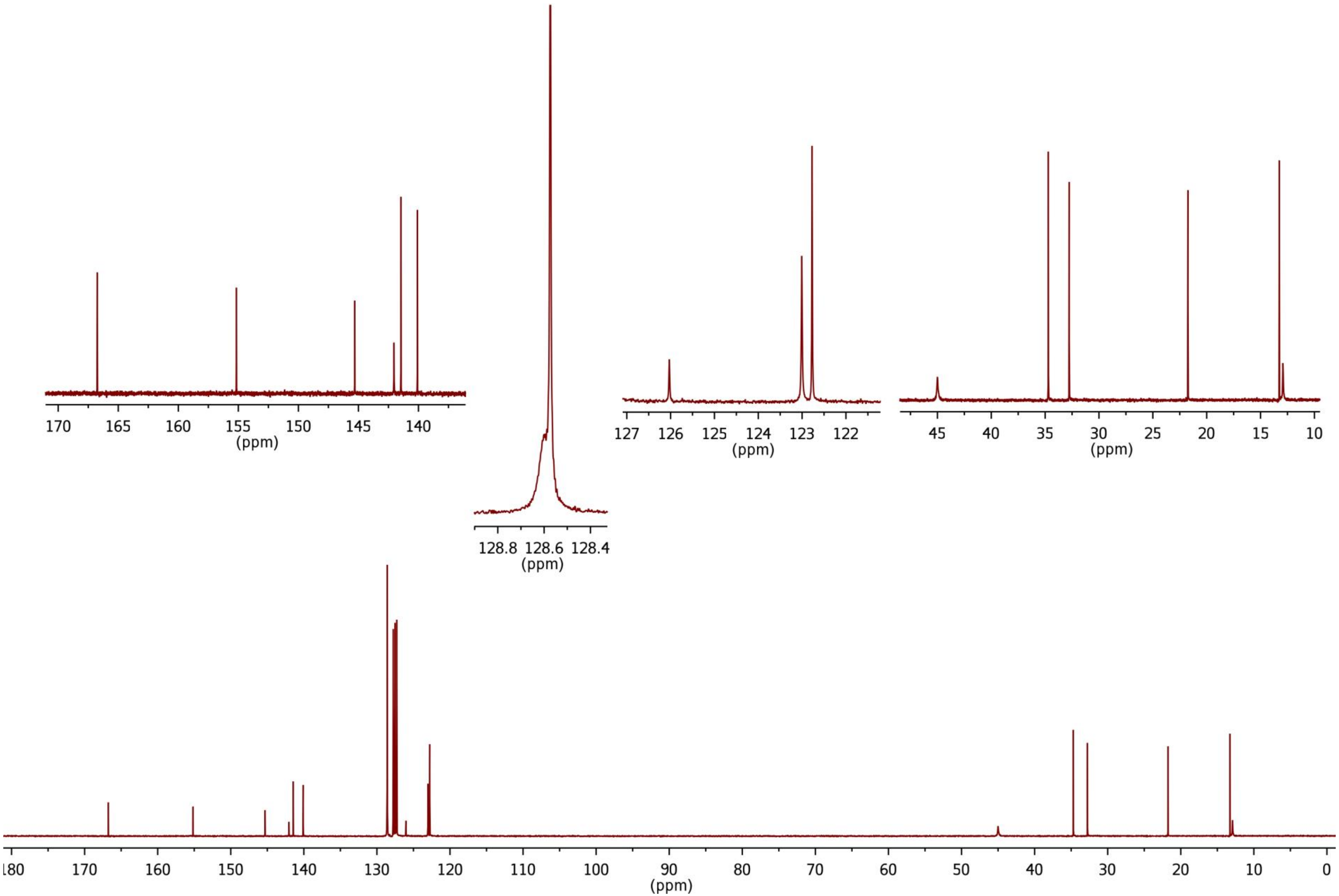

Figure S2. ^{13}C NMR spectrum in C_6D_6 at 60ºC

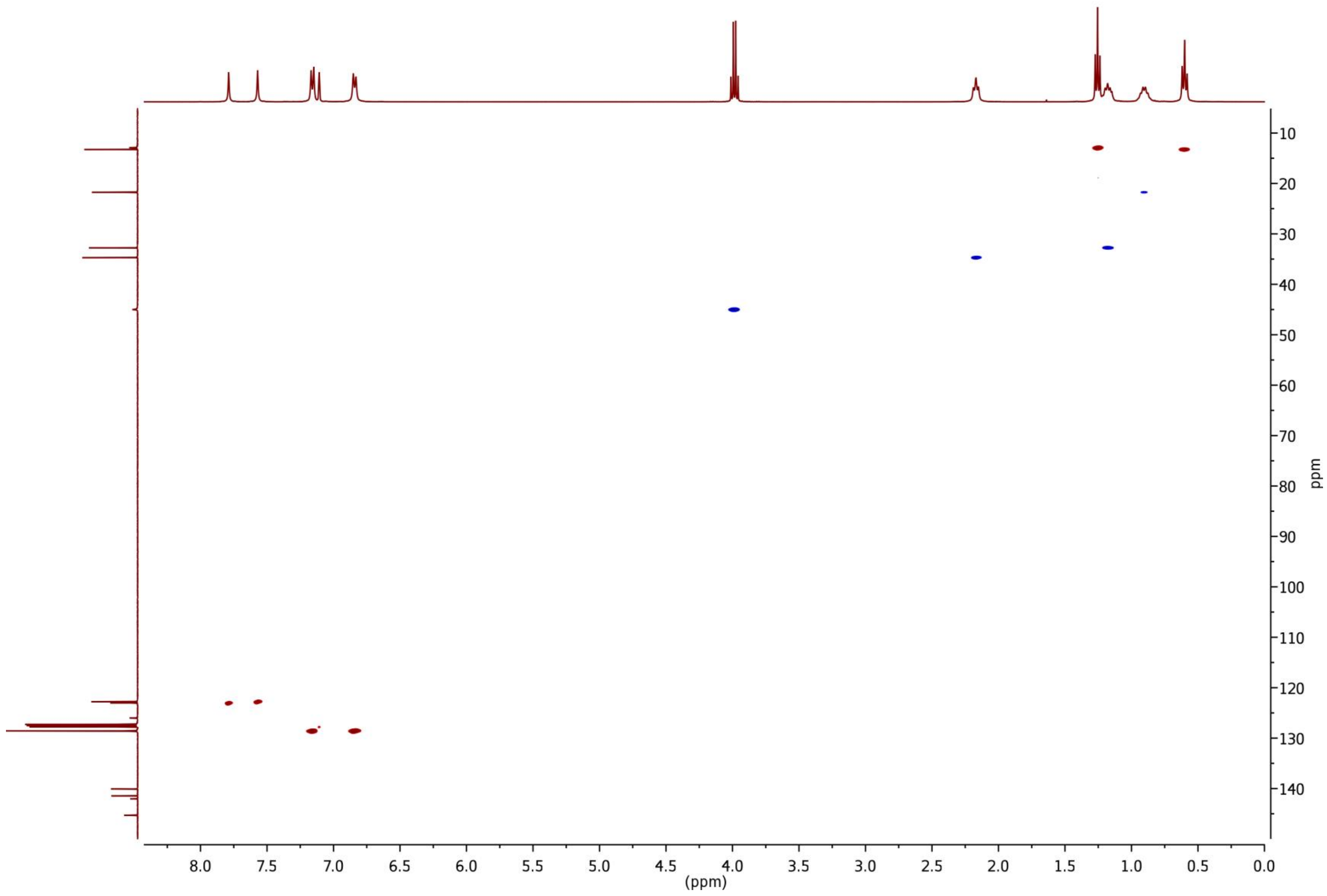

Figure S3. $^1H/^{13}C$ HSQC NMR spectrum in C_6D_6 at 60ºC

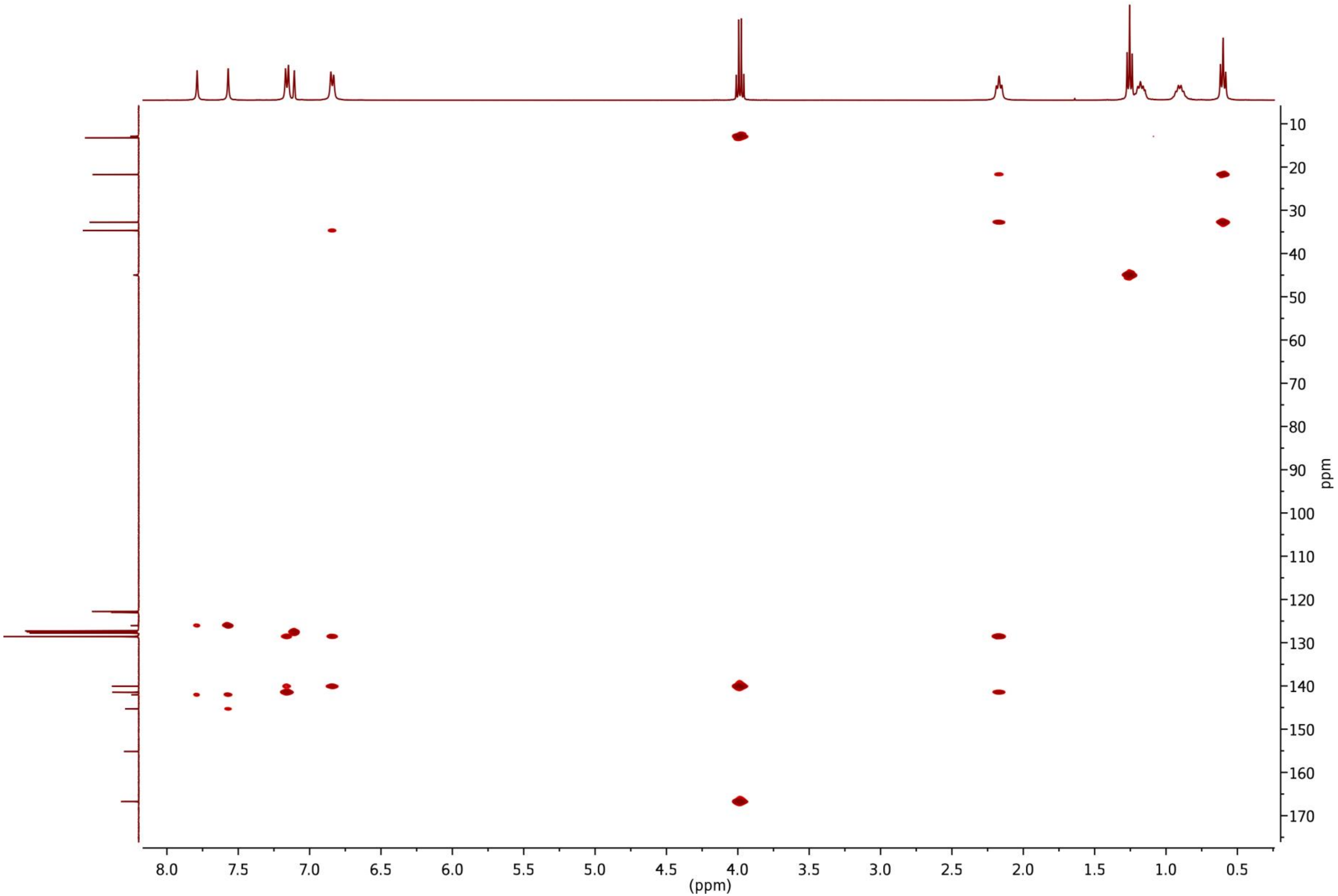

Figure S4. $^{1}H/^{13}C$ HMBC NMR spectrum in C_6D_6 at 60ºC

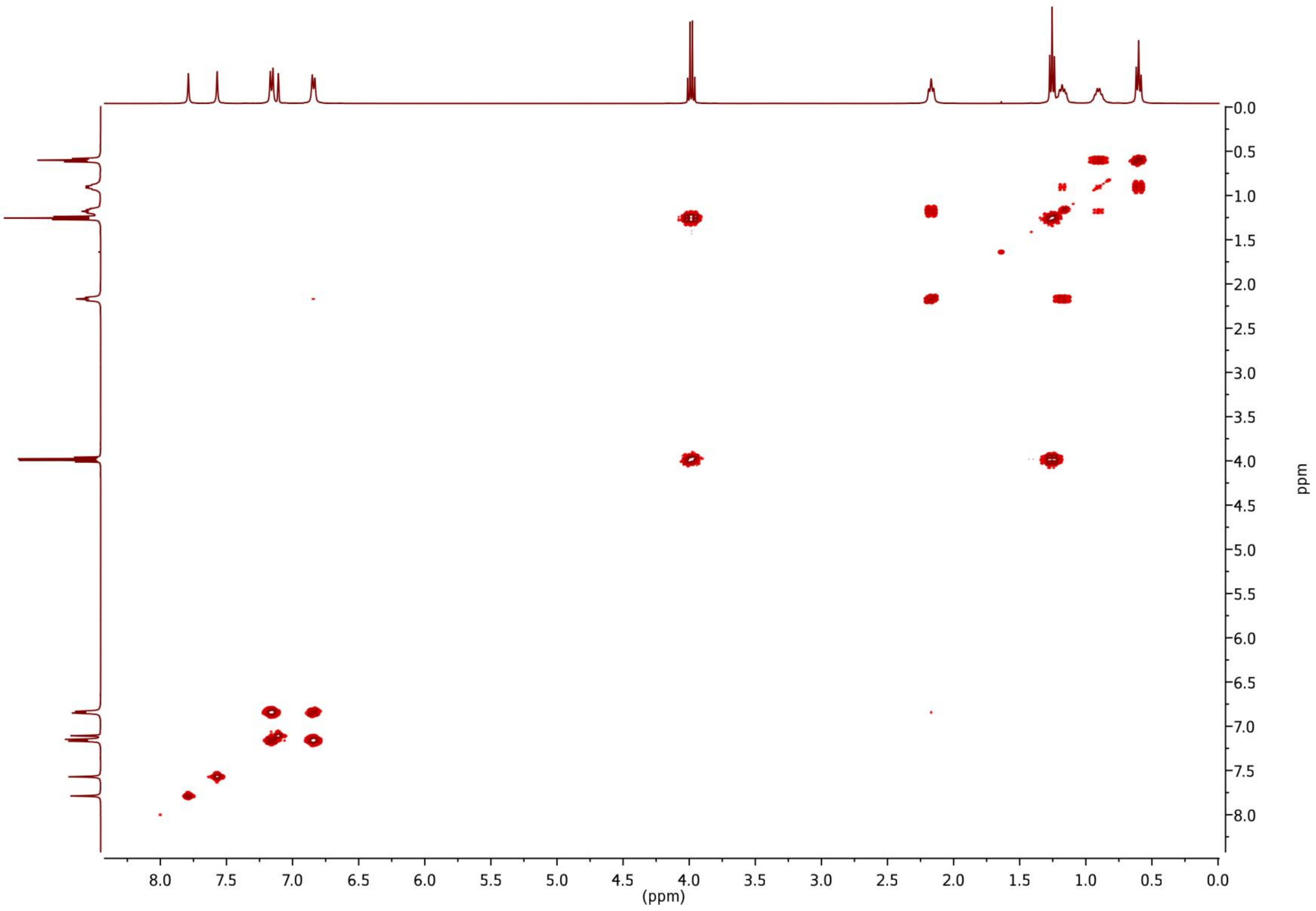

Figure S5. $^{1}H/^{1}H$ COSY NMR spectrum in C_6D_6 at 60ºC

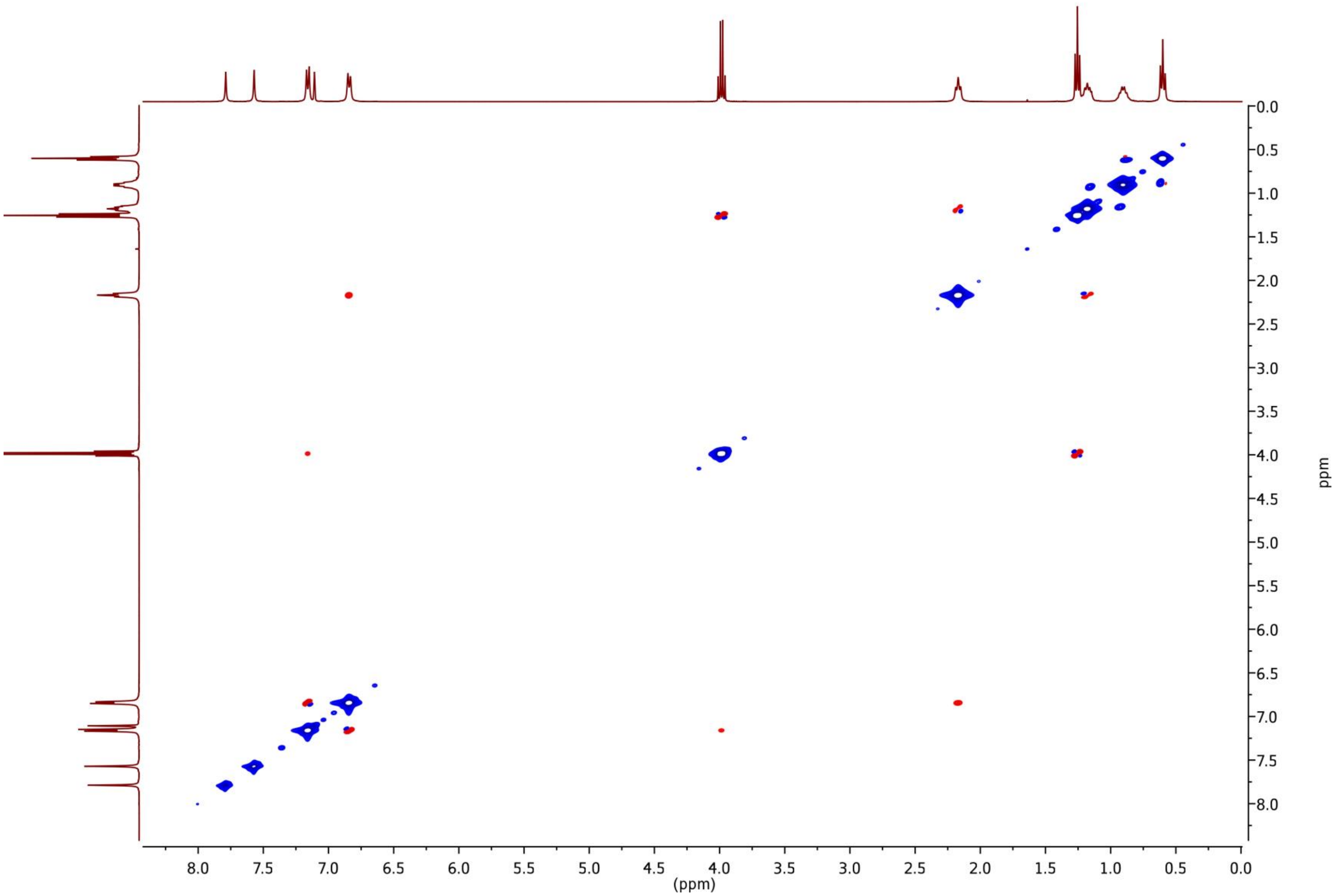

Figure S6. $^{1}H/^{1}H$ NOESY NMR spectrum in C_6D_6 at 60ºC

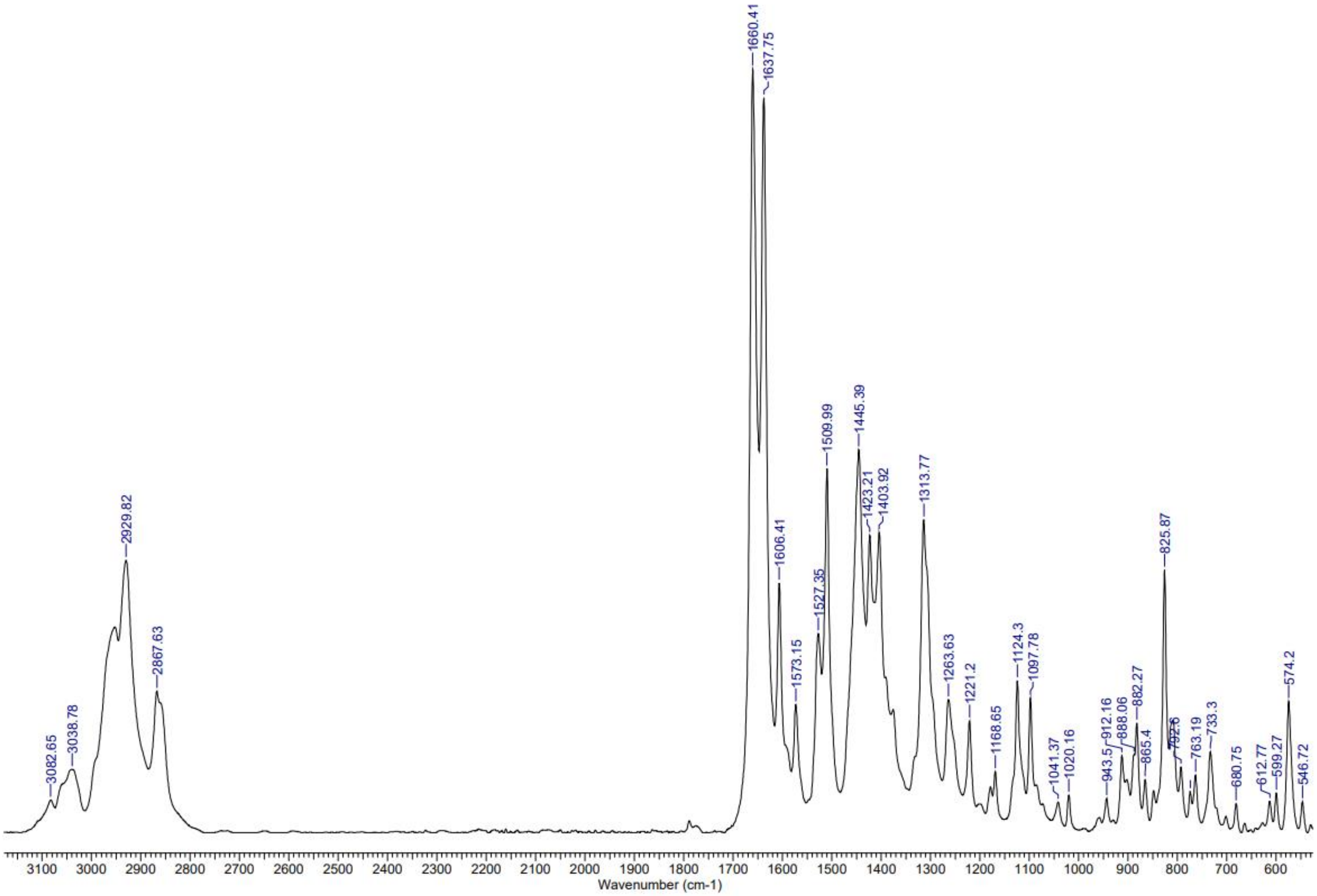

Figure S7. Solid-state IR spectrum at 25ºC

4,7-difluoro-2,9-dimethyl-1,10-phenanthroline (6).

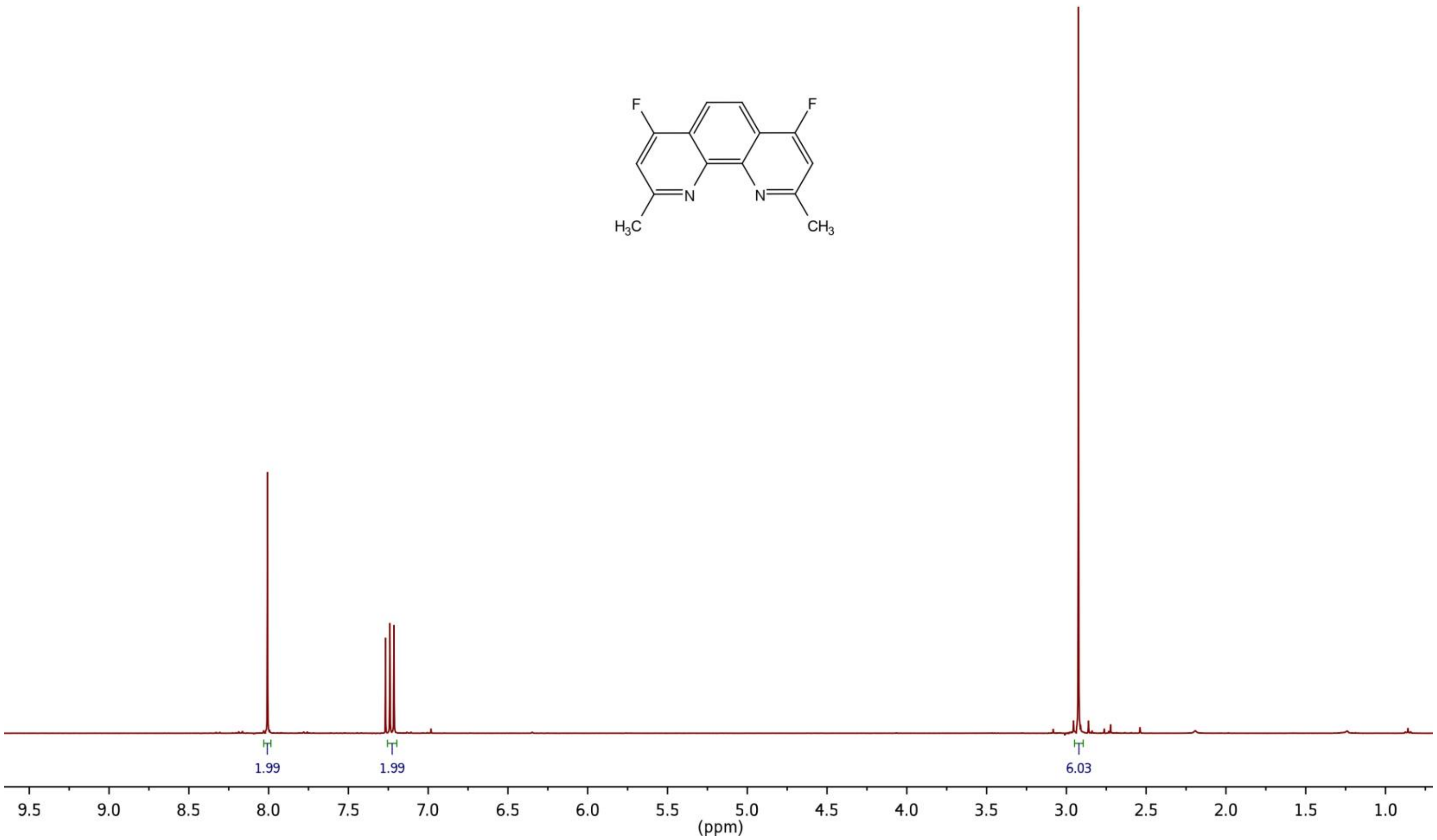

Figure S8. ^{1}H NMR spectrum in $CDCl_3$ at 25ºC

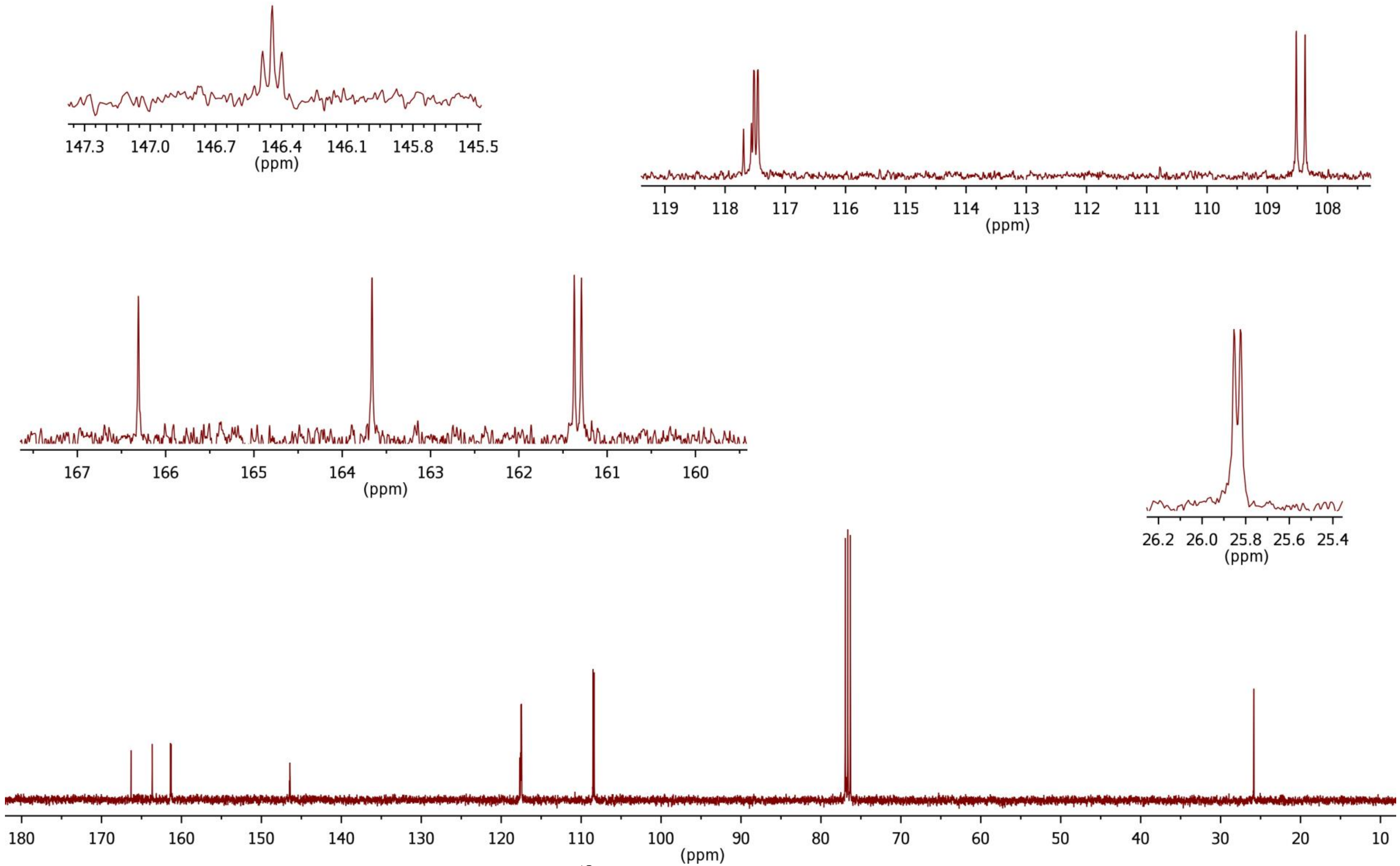

Figure S9. ^{13}C NMR spectrum in $CDCl_3$ at 25ºC

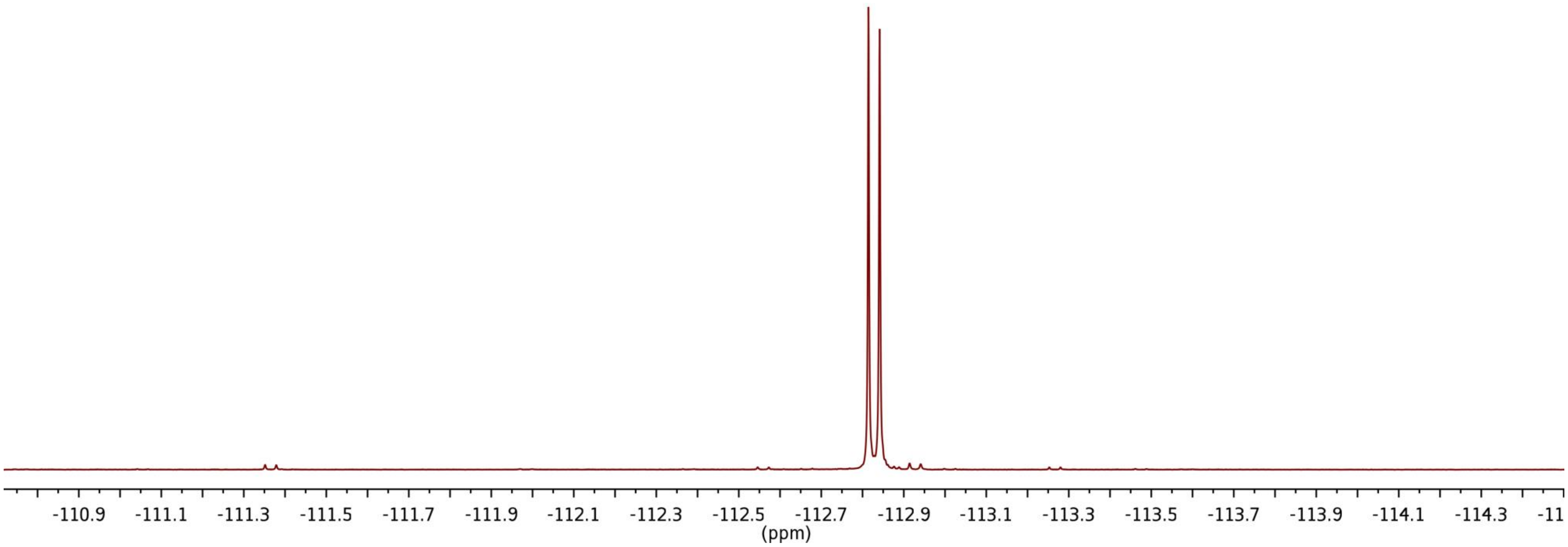

Figure S10. ^{19}F NMR spectrum in $CDCl_3$ at 25ºC

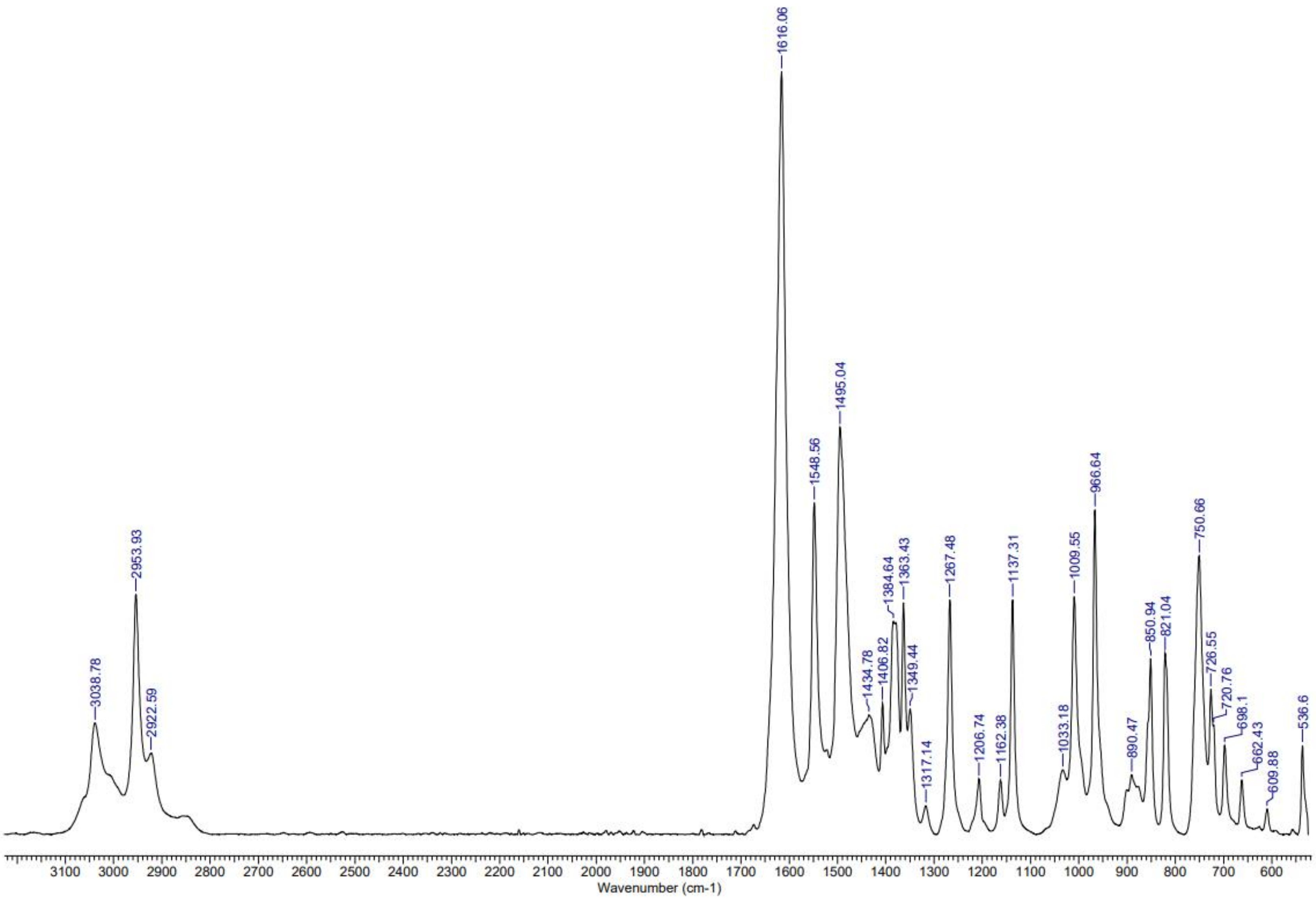

Figure S11. Solid-state IR spectrum at 25ºC

N^2,N^2,N^9,N9- tetrabutyl -4,7- difluoro -1,10- phenanthroline -2,9- dicarboxamide (7a)

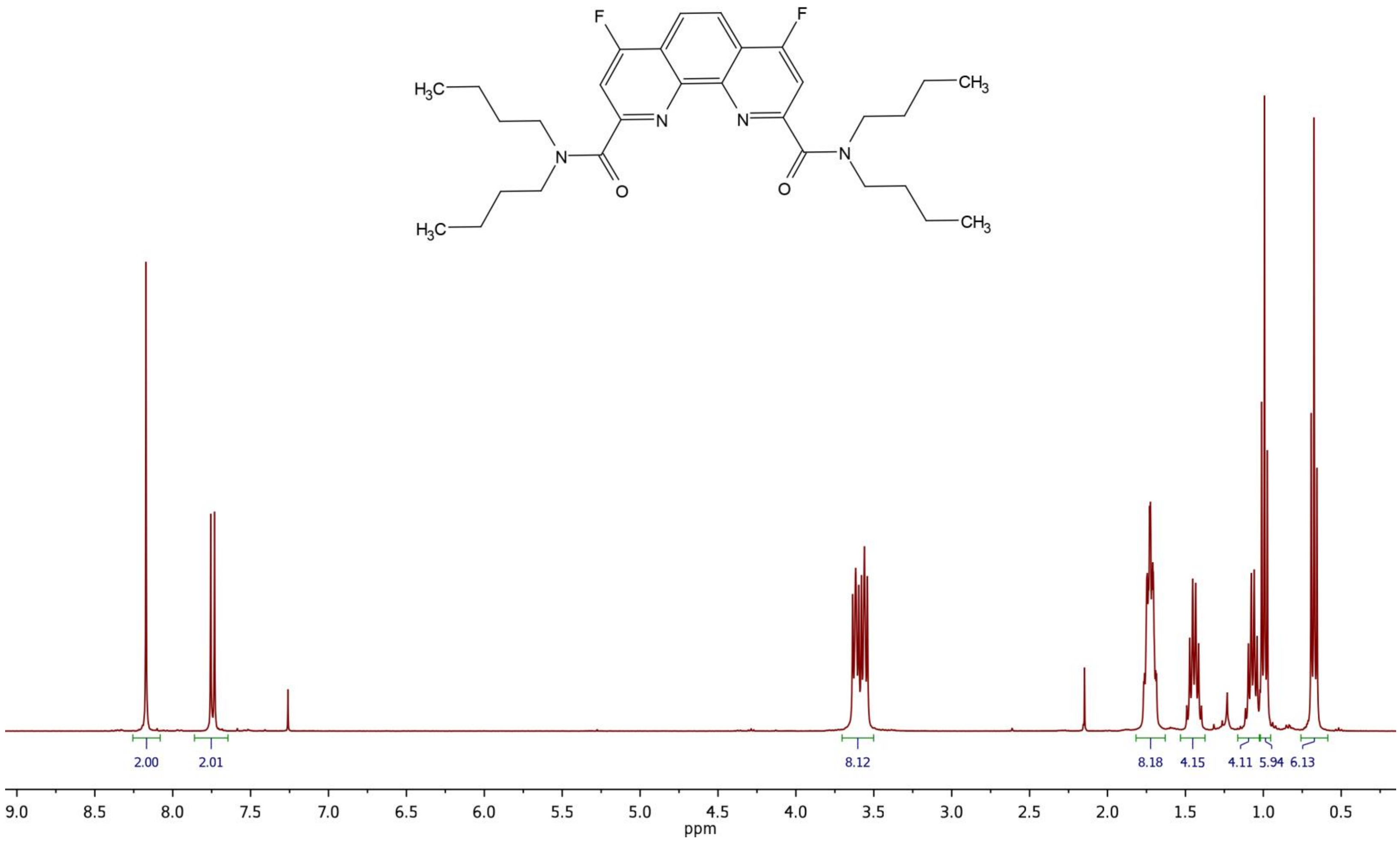

Figure S12. ^{1}H NMR spectrum in $CDCl_3$ at 25ºC

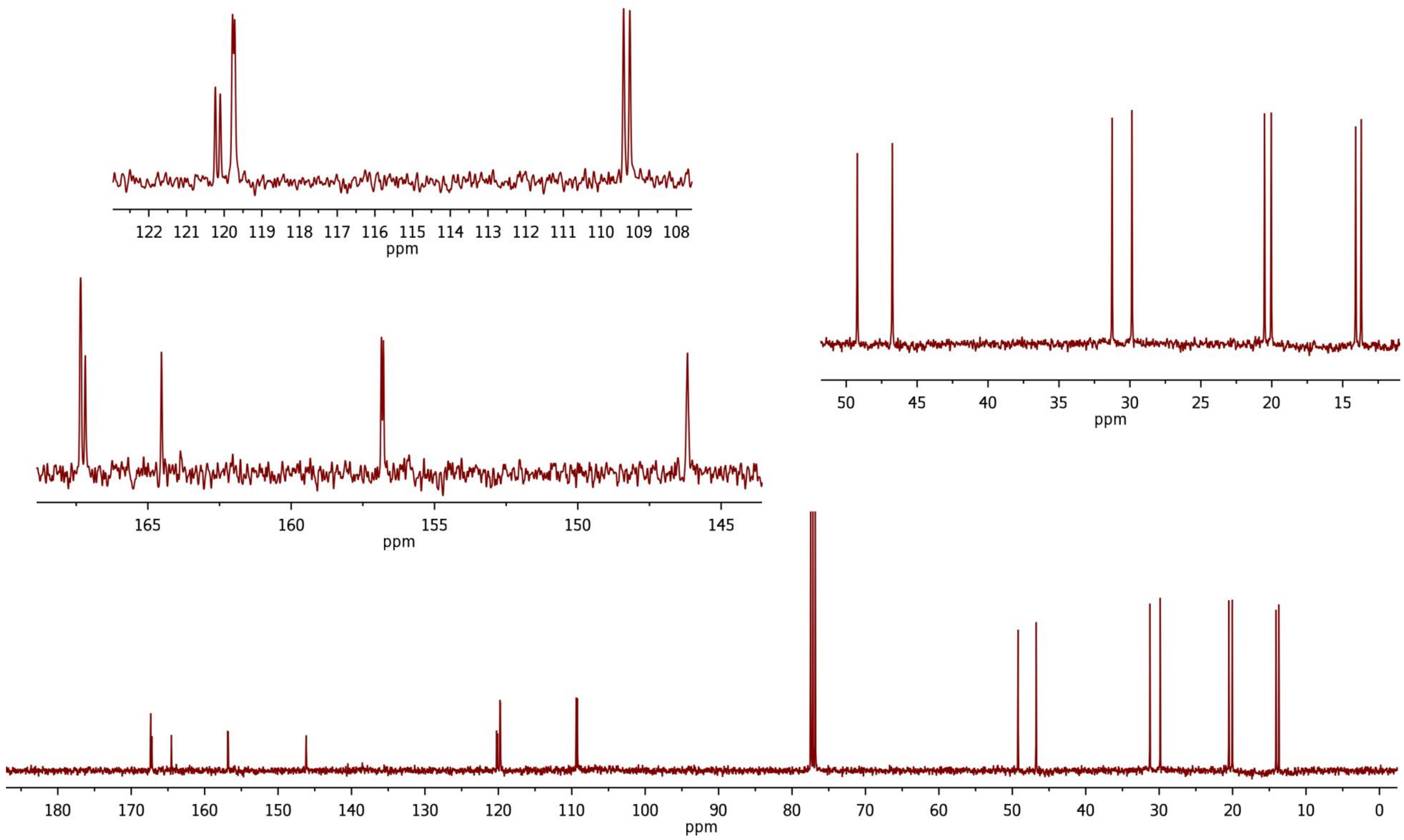

Figure S13. ^{13}C NMR spectrum in $CDCl_3$ at 25ºC

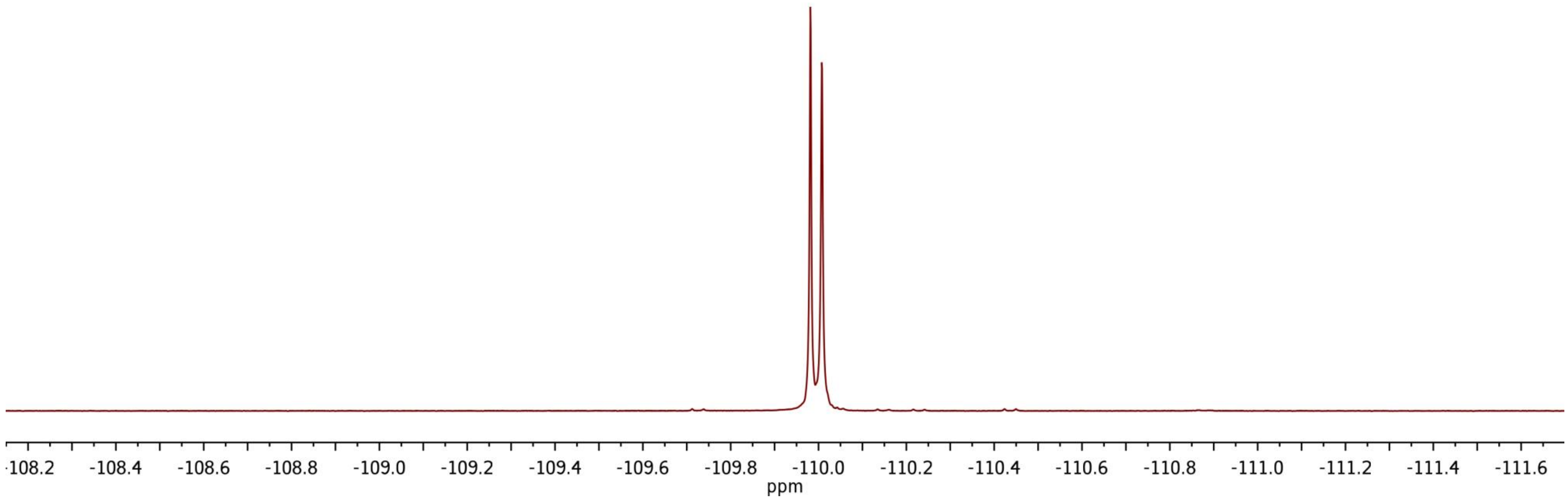

Figure S14. ^{19}F NMR spectrum in $CDCl_3$ at 25ºC

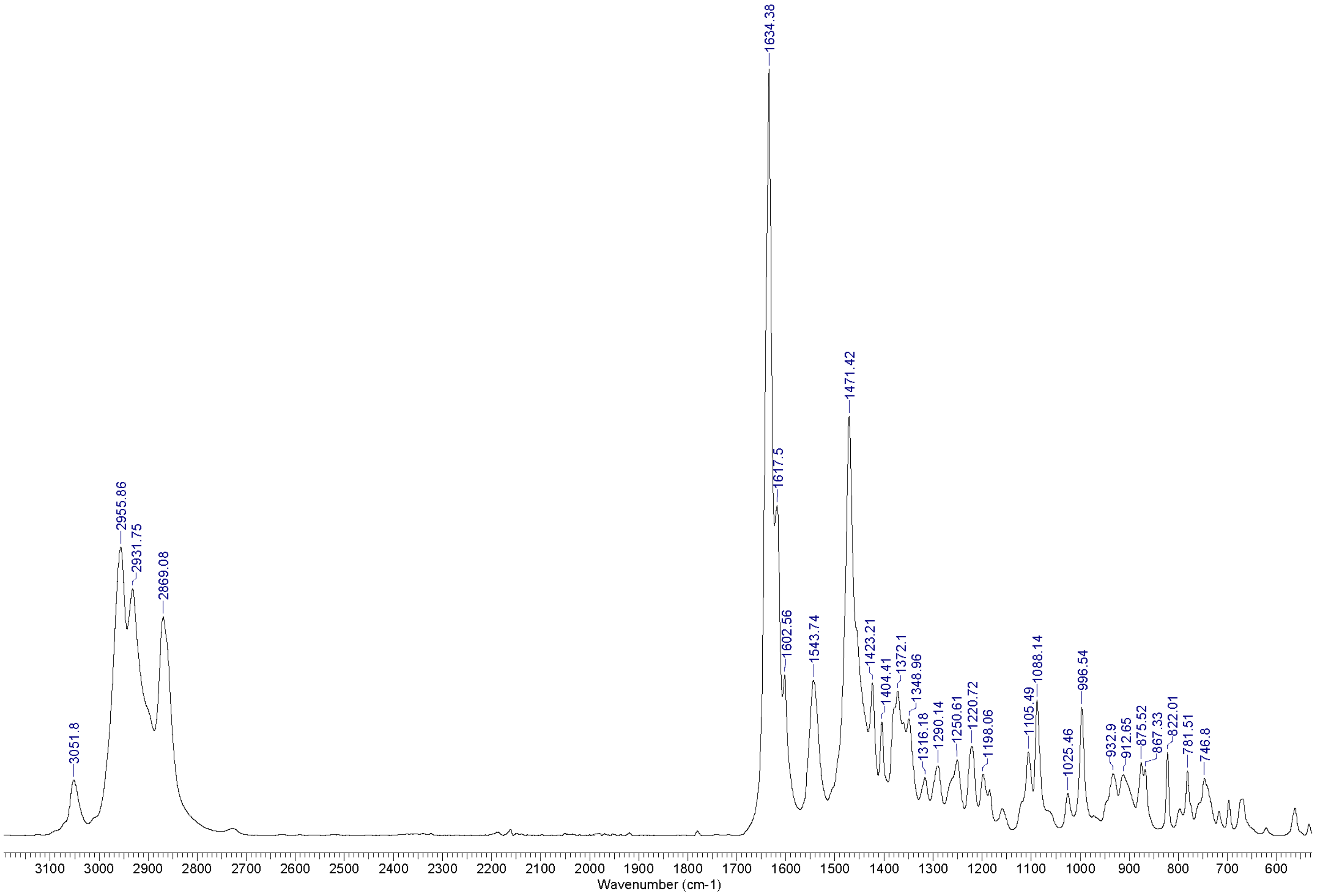

Figure S15. Solid-state IR spectrum at 25ºC

N^2,N^9- bis(pyrrolidine) -4,7- difluoro -N^2,N^9- diethyl -1,10- phenanthroline-2,9- dicarboxamide (7b)

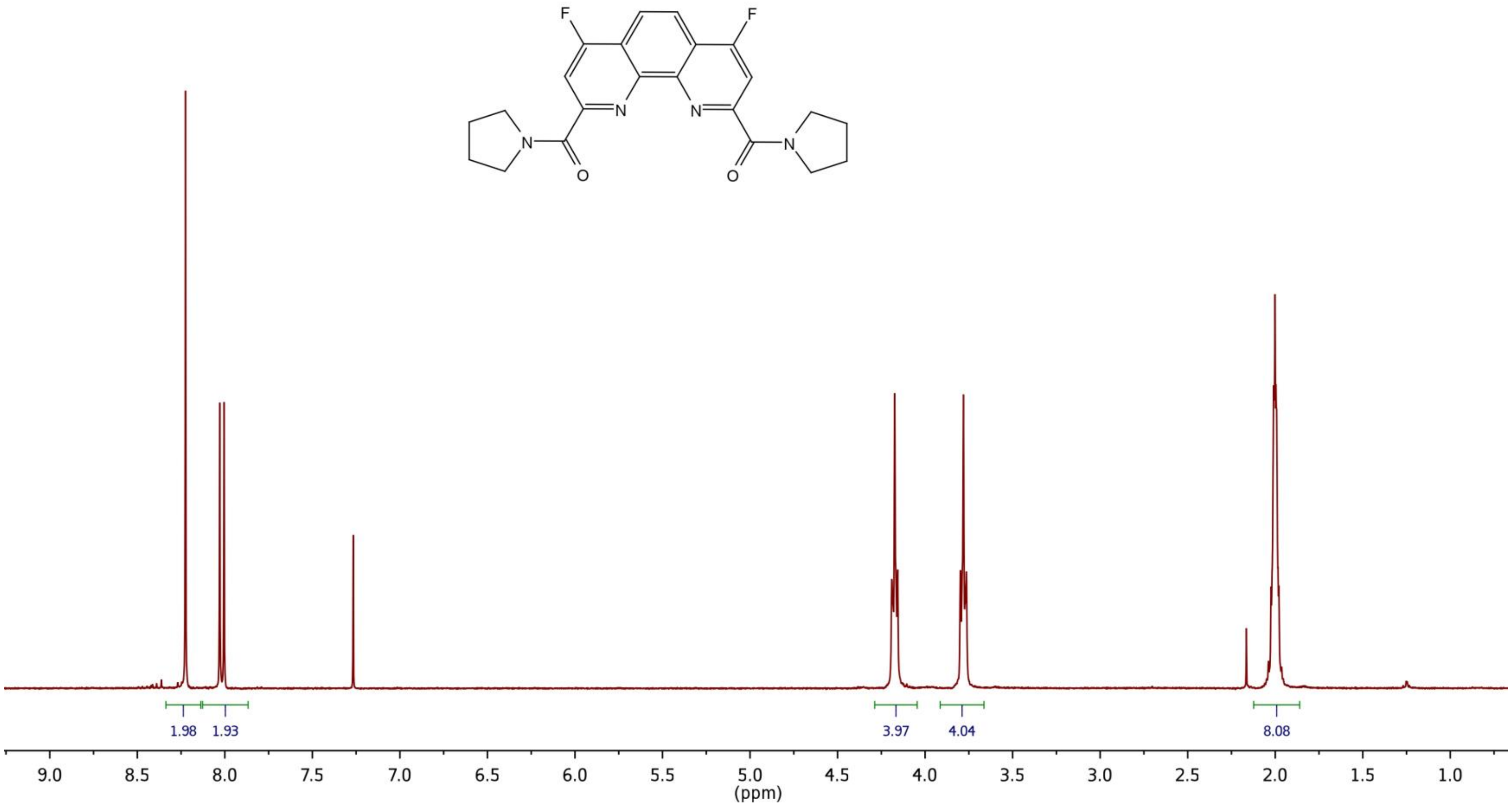

Figure S16. ^{1}H NMR spectrum in $CDCl_3$ at 25ºC

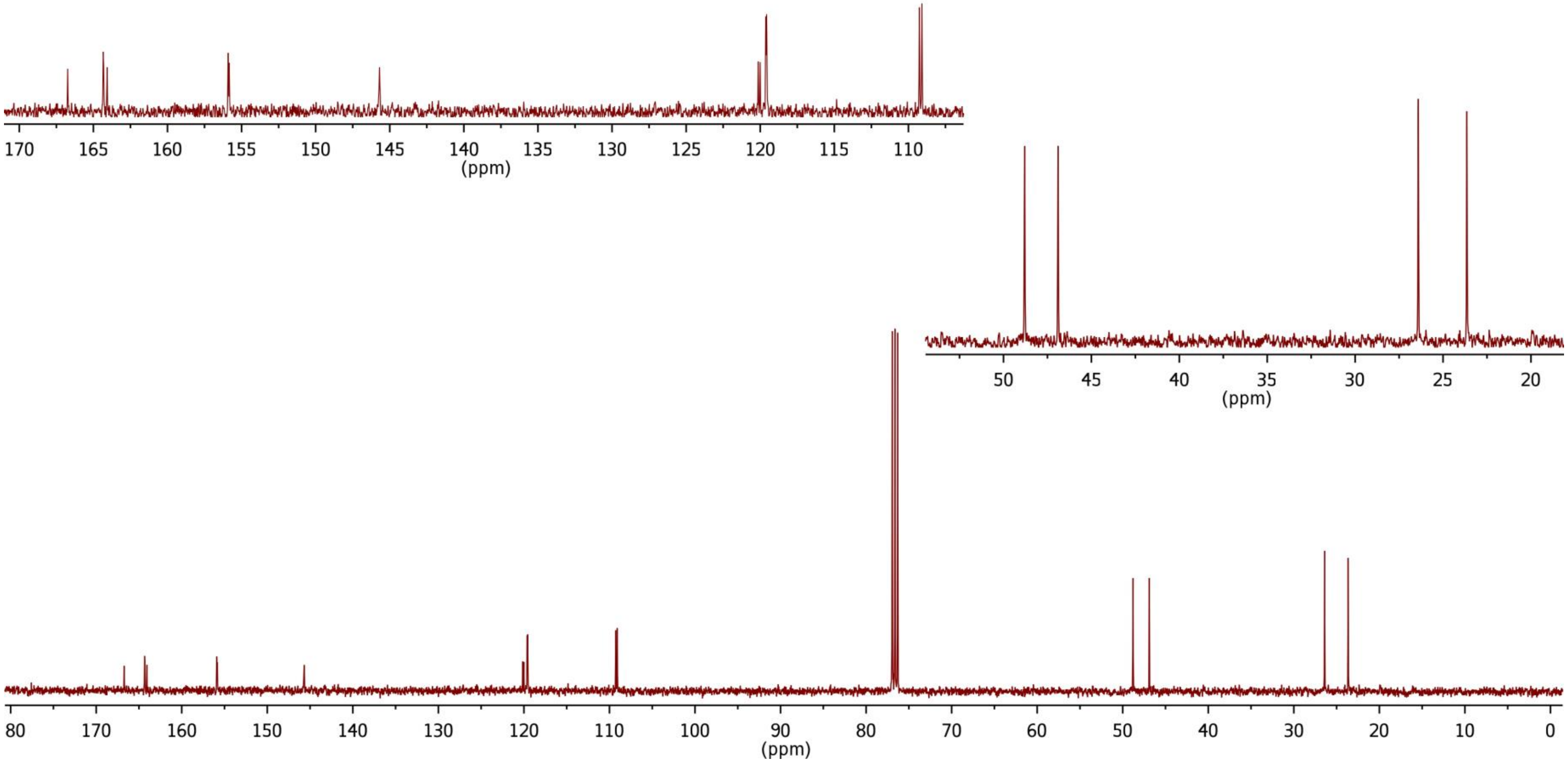

Figure S17. ^{13}C NMR spectrum in $CDCl_3$ at 25ºC

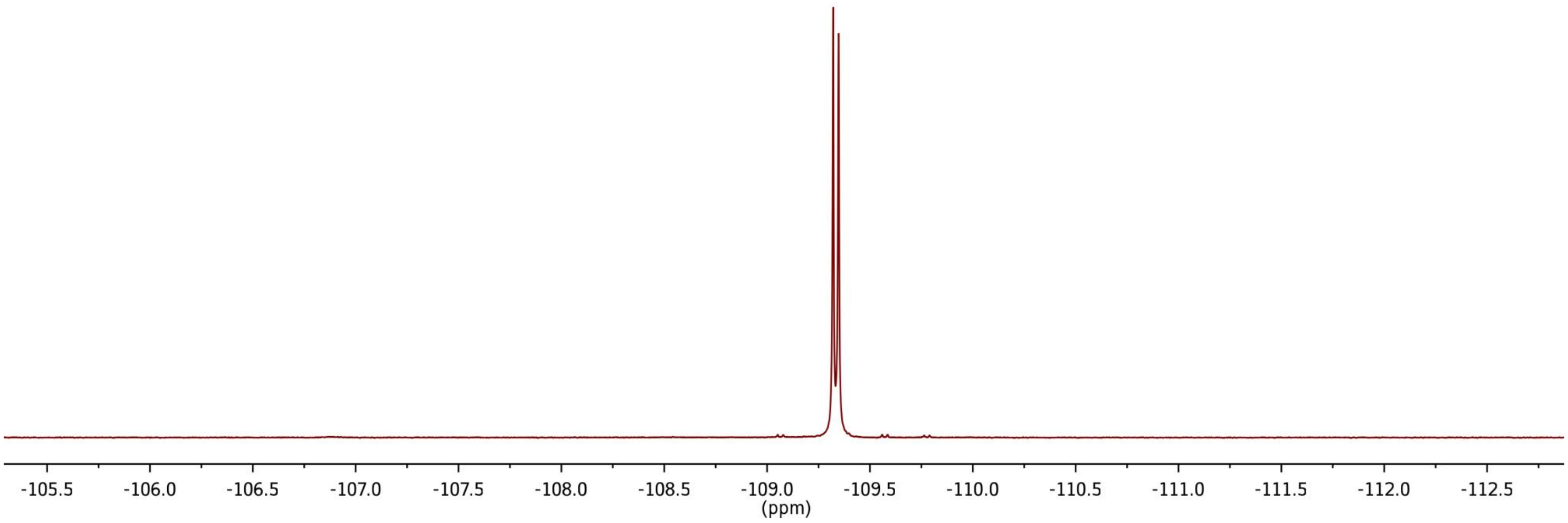

Figure S18. ^{19}F NMR spectrum in $CDCl_3$ at 25ºC

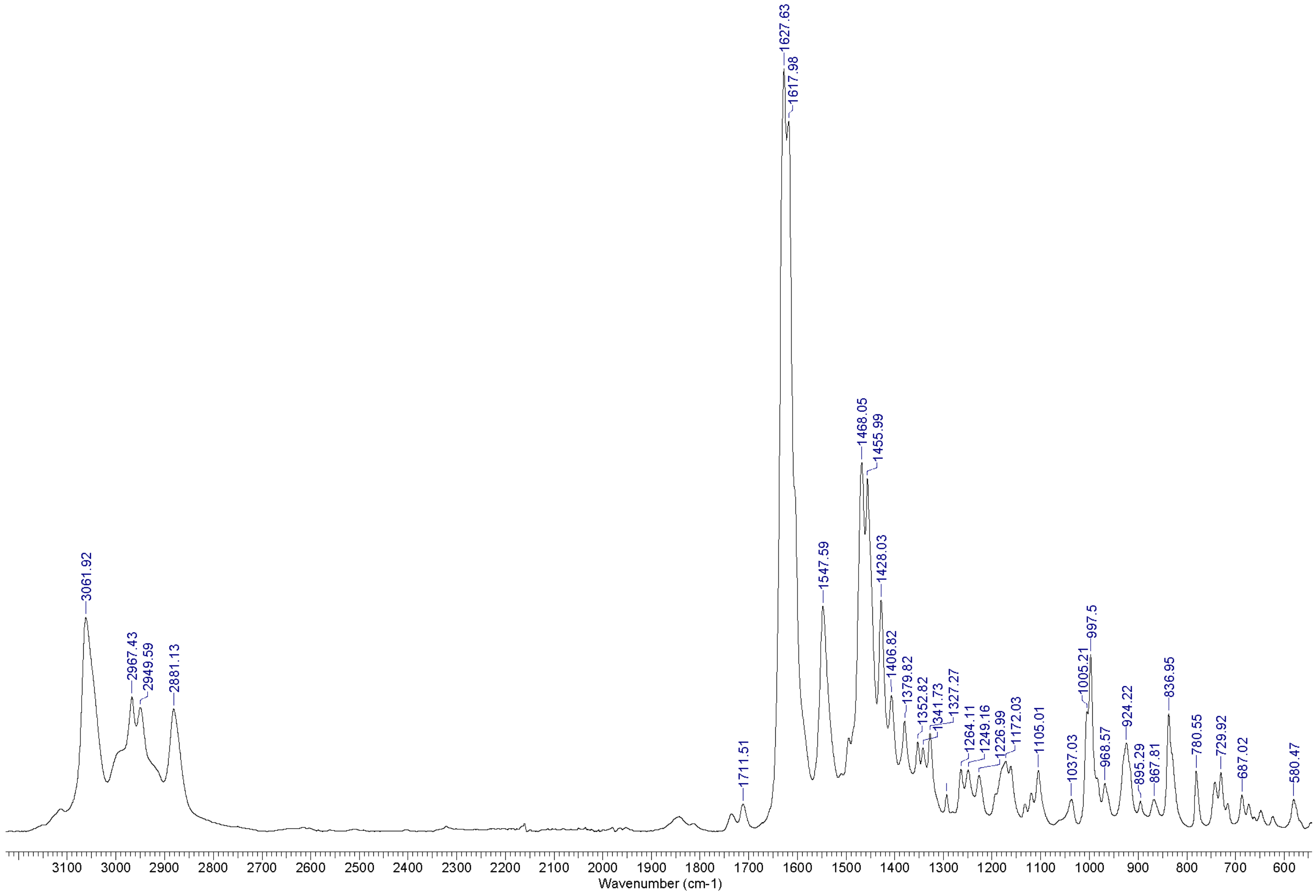

Figure S19. Solid-state IR spectrum at 25ºC

N^2,N^9-bis(4-butylphenyl)-4,7-difluoro-N^2,N^9-diethyl-1,10-phenanthroline-2,9-dicarboxamide (7c)

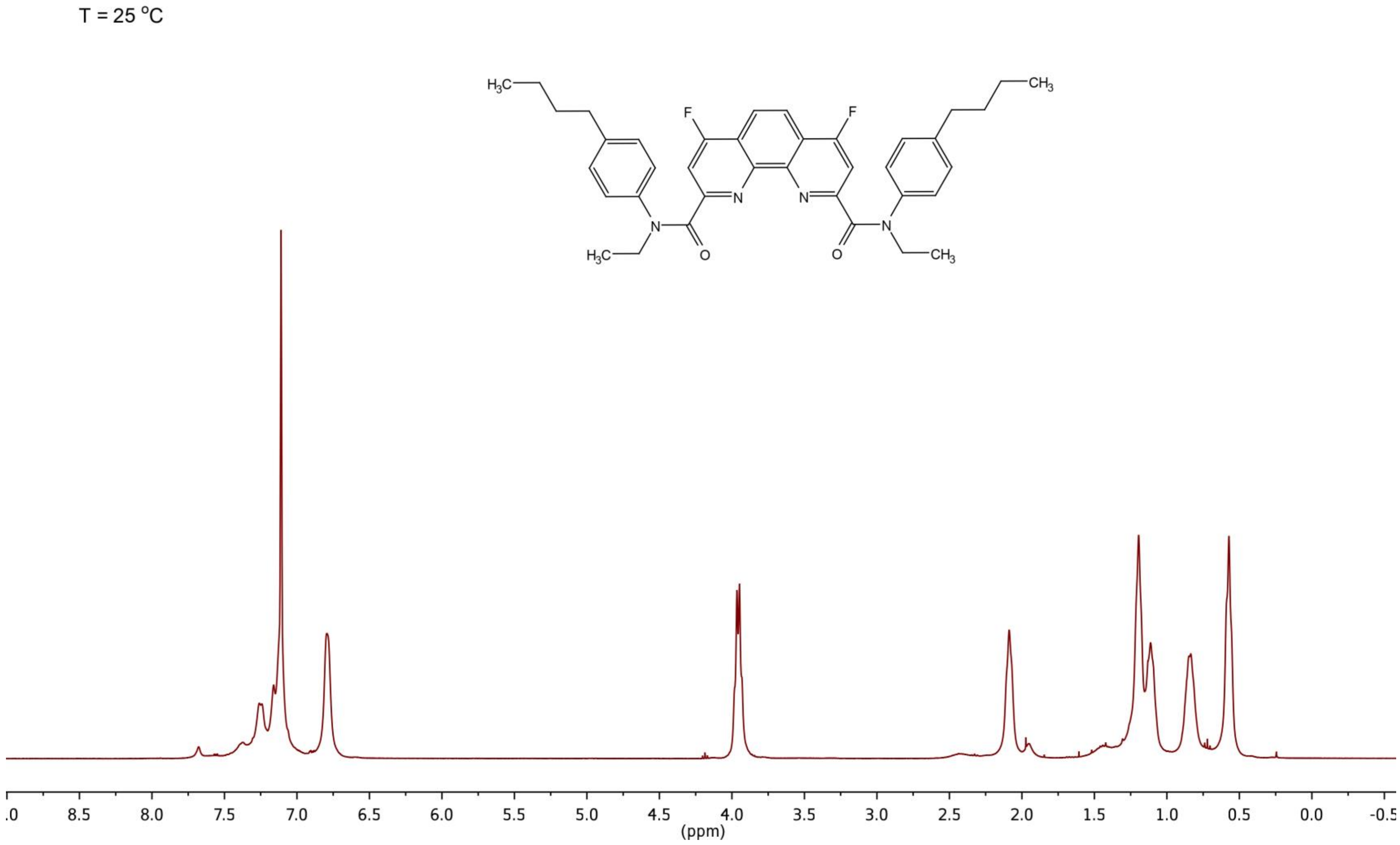

Figure S20. ^{1}H NMR spectrum in C_6D_6 at 25ºC

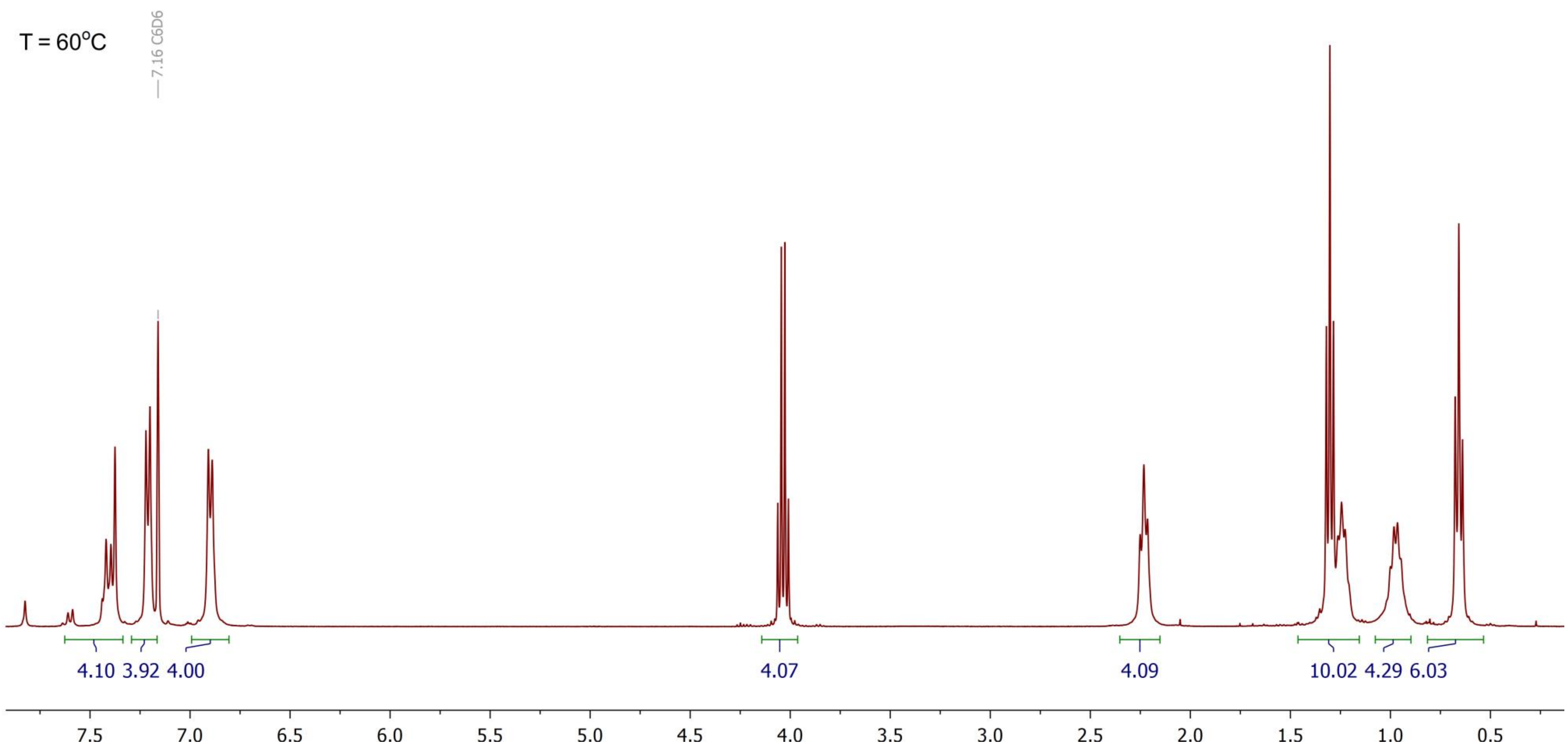

Figure S21. ^{1}H NMR spectrum in C_6D_6 at 60ºC

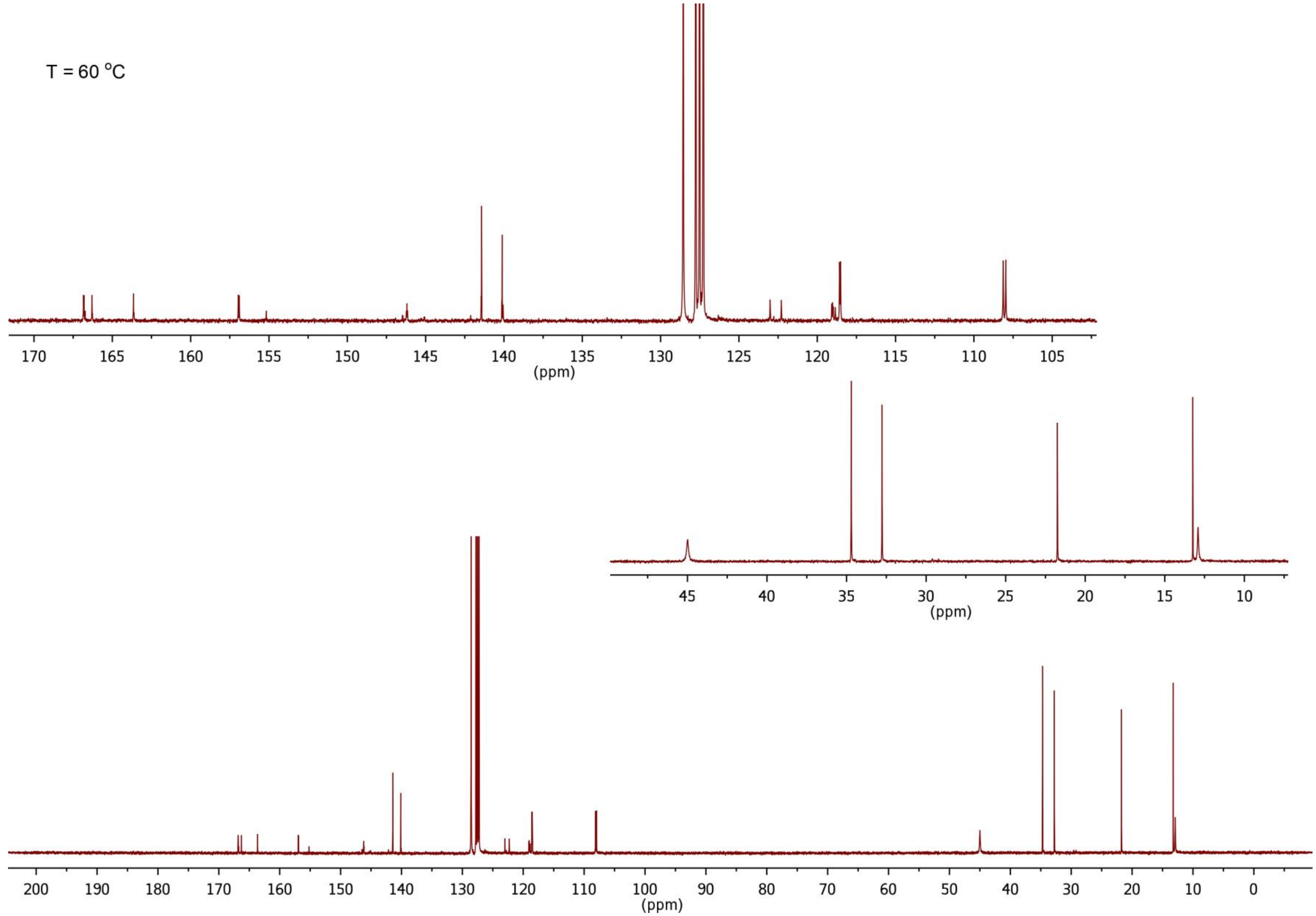

Figure S22. ^{13}C NMR spectrum in C_6D_6 at 60ºC

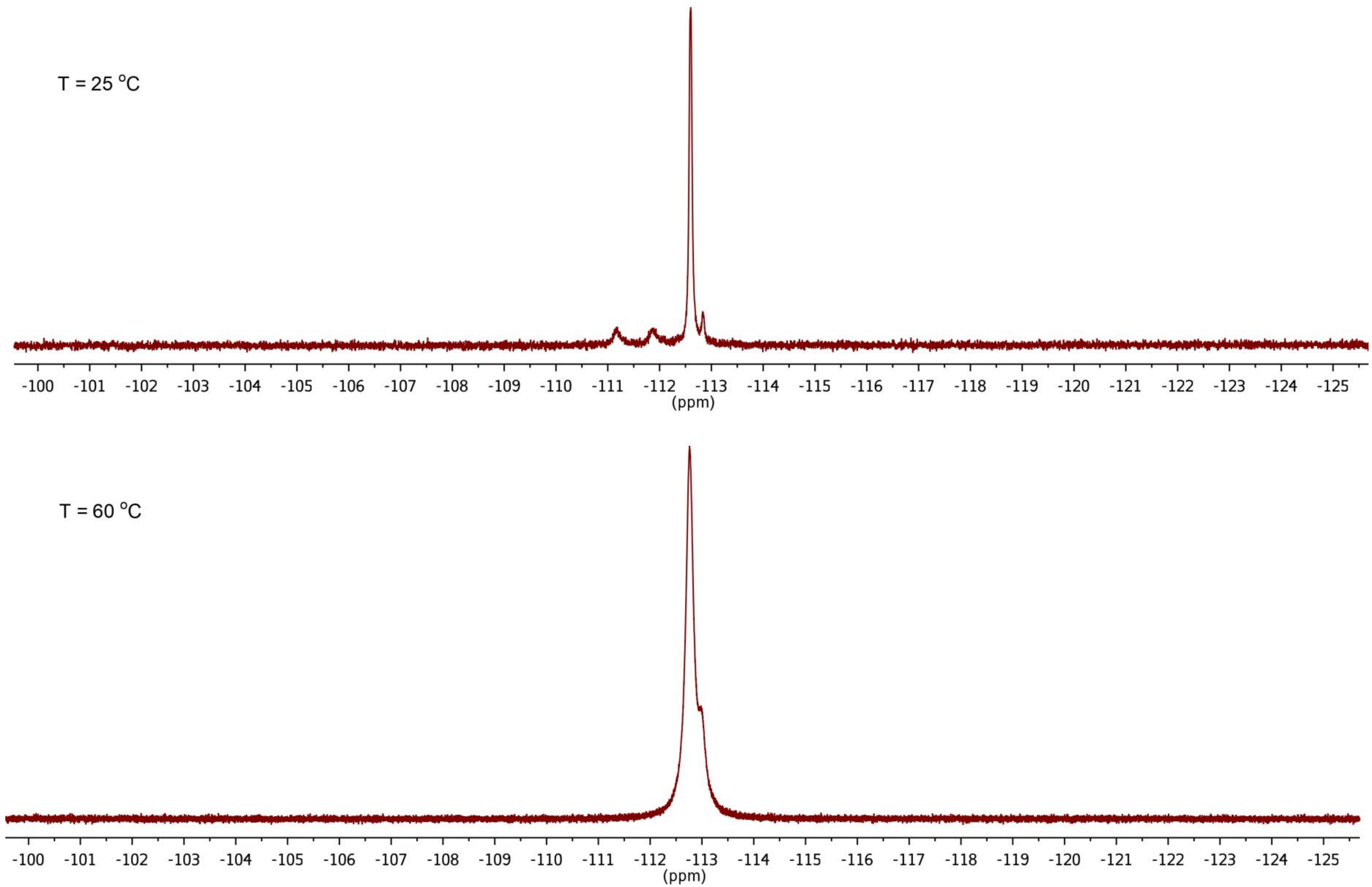

Figure S23. ^{19}F NMR spectra in C_6D_6 at 25 ºC and 60ºC

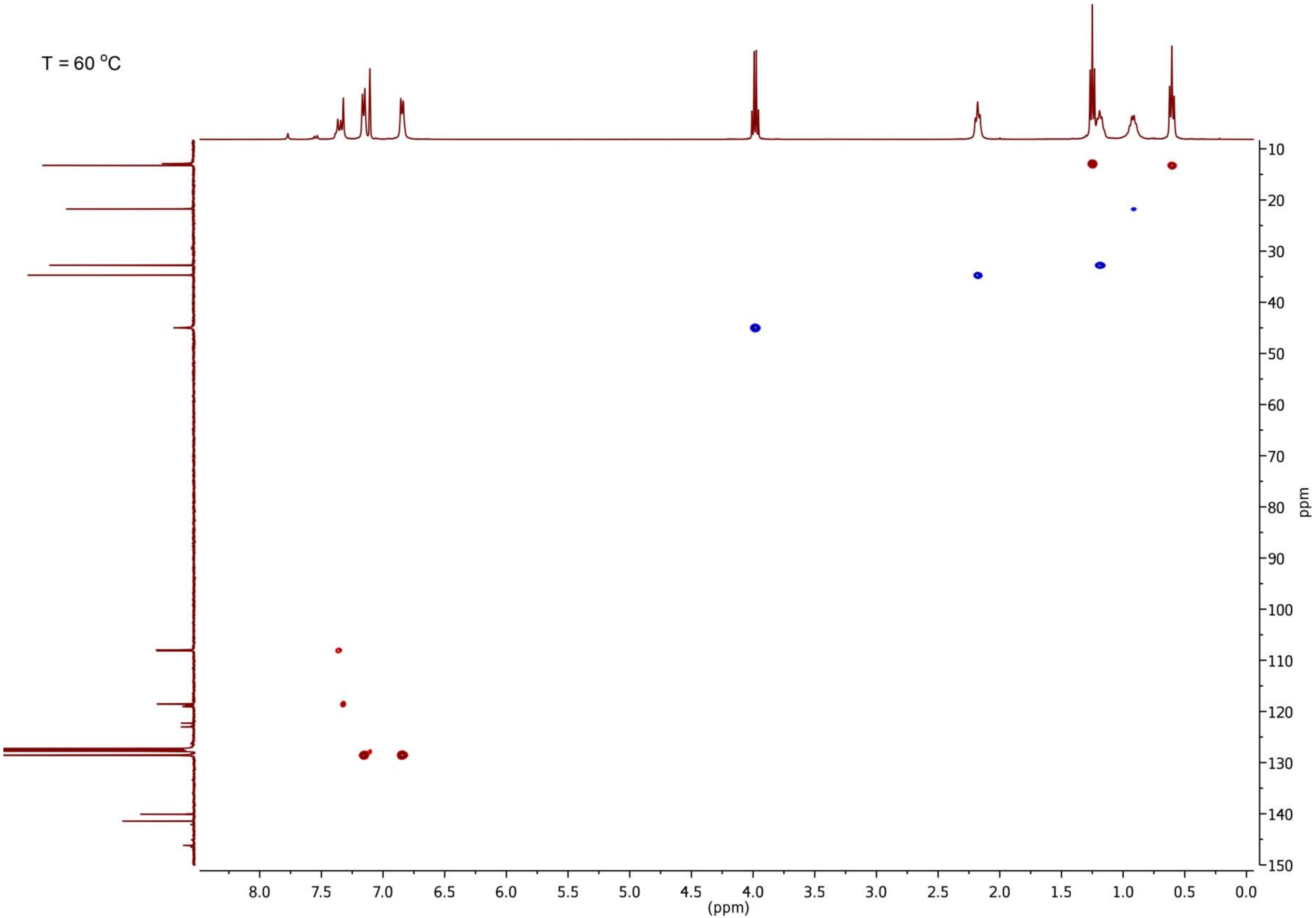

Figure S24. $^{1}H/^{13}C$ HSQC NMR spectrum in C_6D_6 at 60ºC

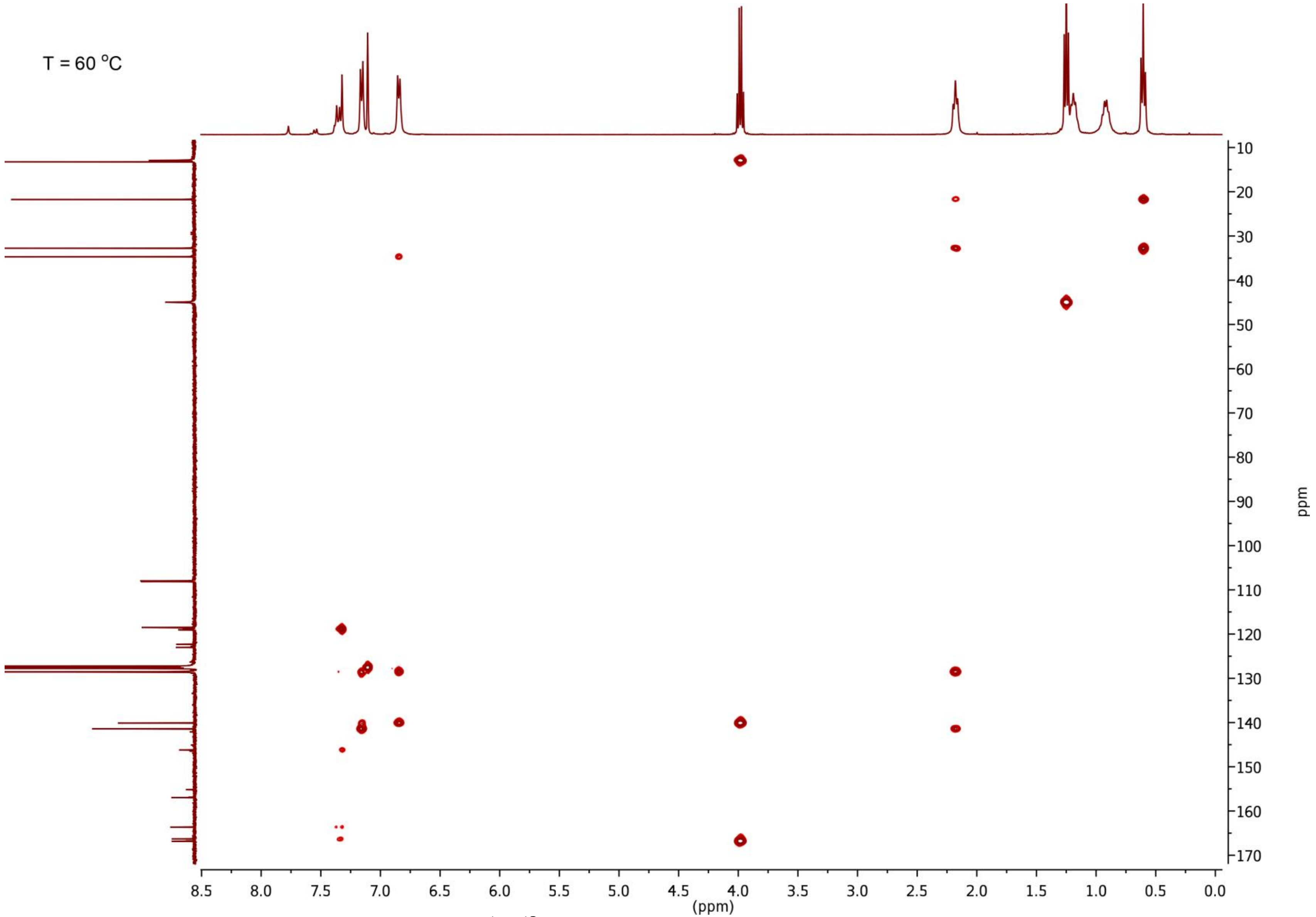

Figure S25. $^{1}H/^{13}C$ HMBC NMR spectrum in C_6D_6 at 60ºC

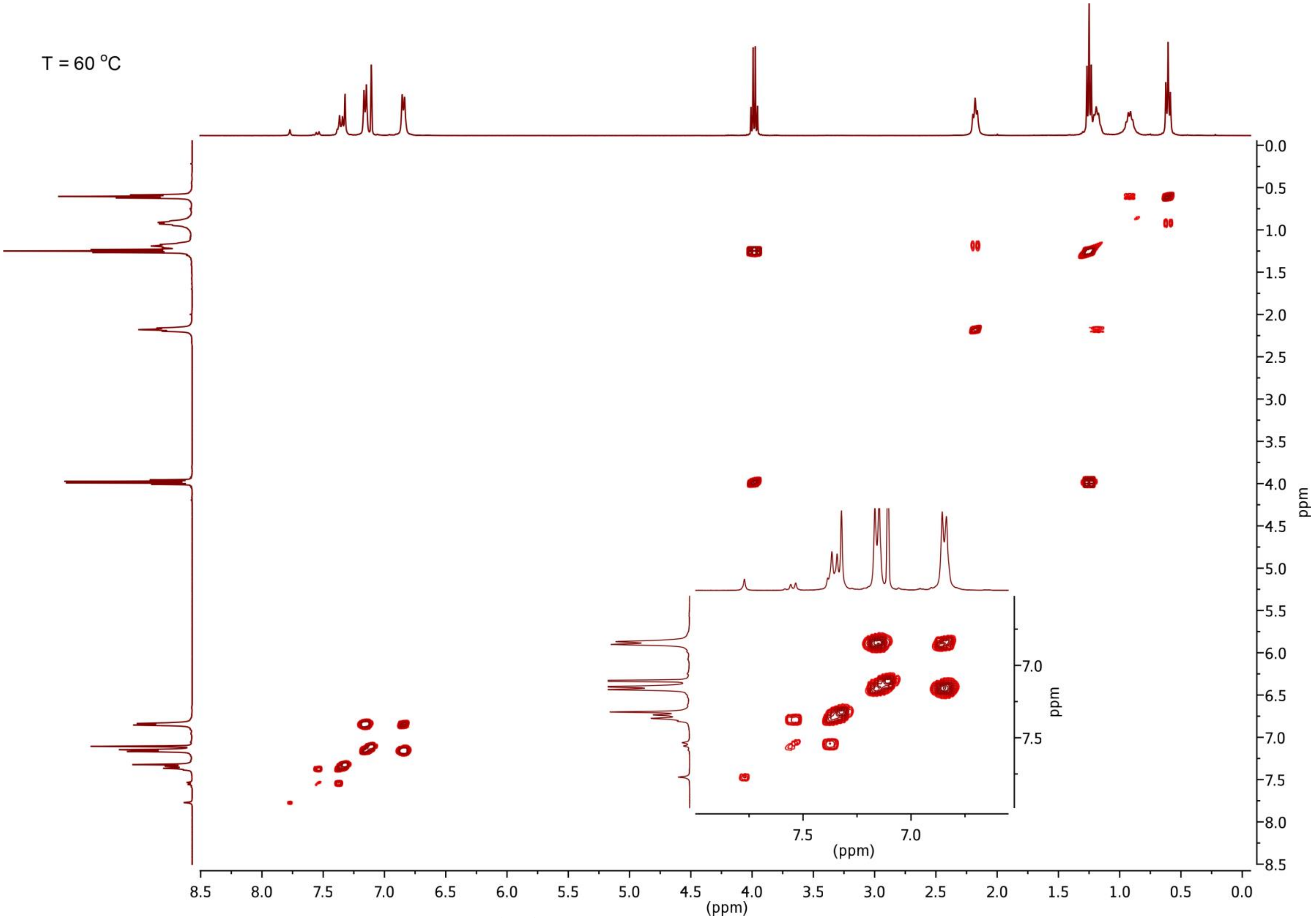

Figure S26. $^1H/^1H$ COSY NMR spectrum in C_6D_6 at 60ºC

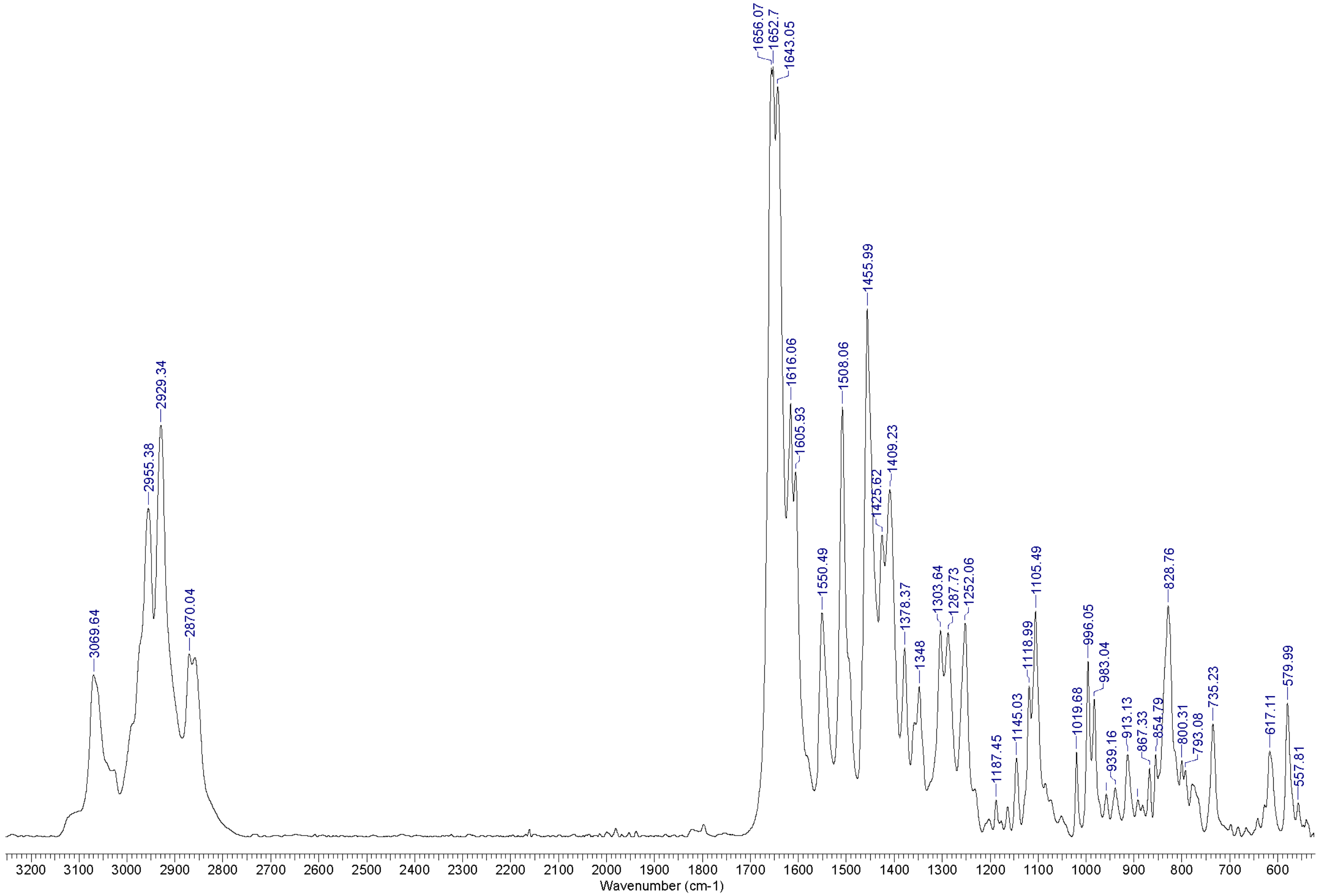

Figure S27. Solid-state IR spectrum at 25ºC

N^2,N^9-bis(4-hexylpheny)-4,7-difluoro-N^2,N^9-diethyl-1,10-phenanthroline-2,9-dicarboxamide (7d)

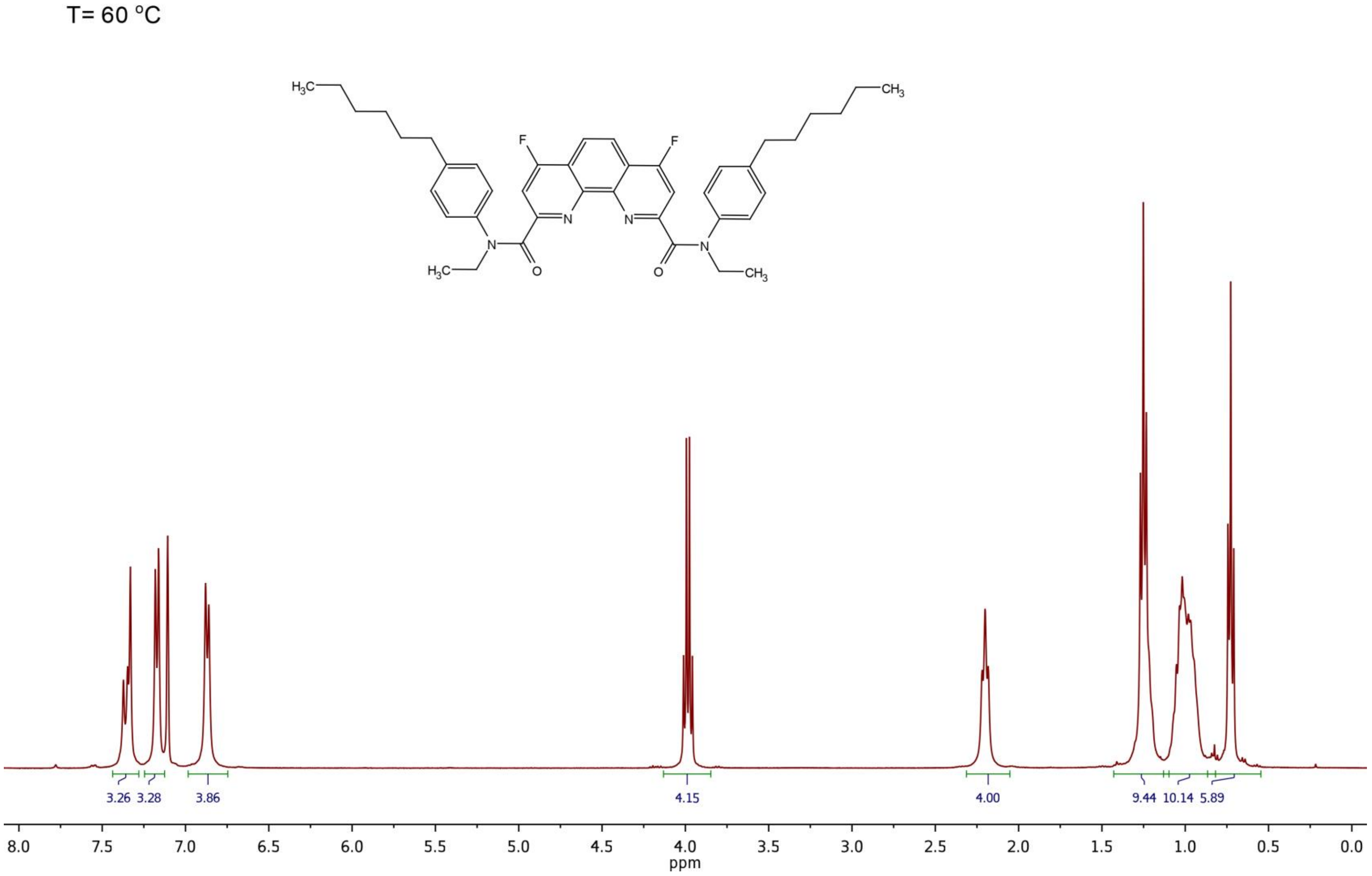

Figure S28. ^{1}H NMR spectrum in C_6D_6 at 60ºC

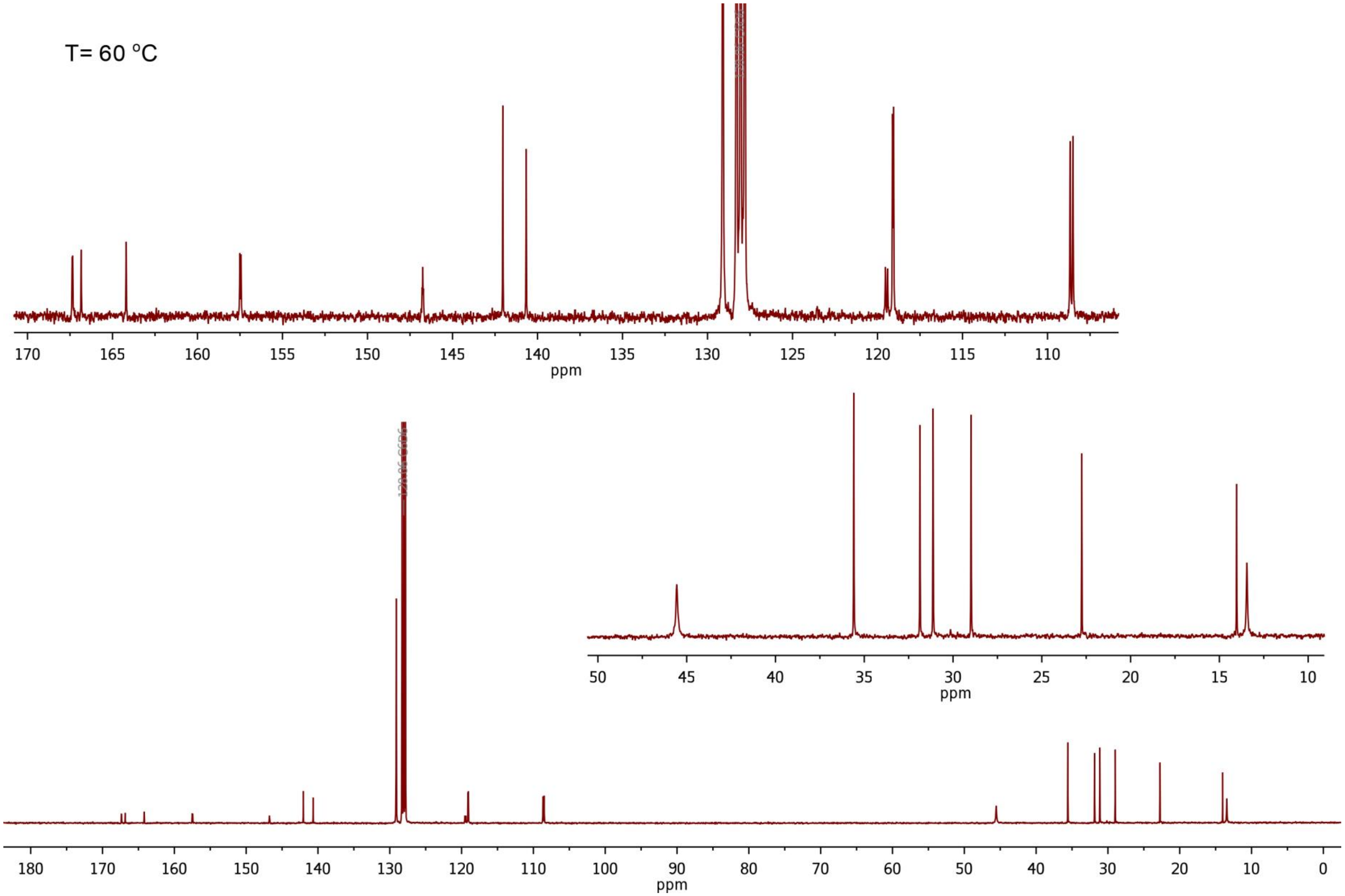

Figure S29. ^{13}C NMR spectrum in C_6D_6 at 60ºC

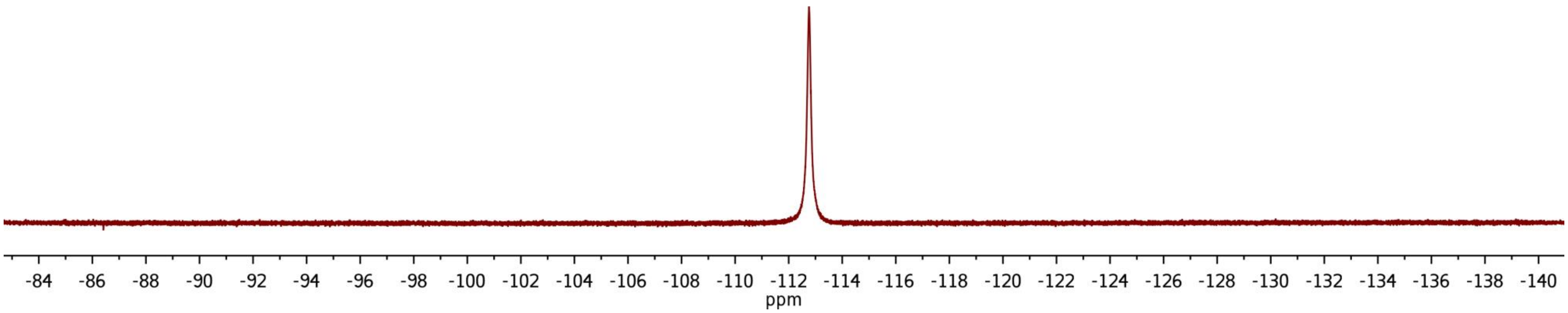

Figure S30. ^{19}F NMR spectrum in C_6D_6 at 60ºC

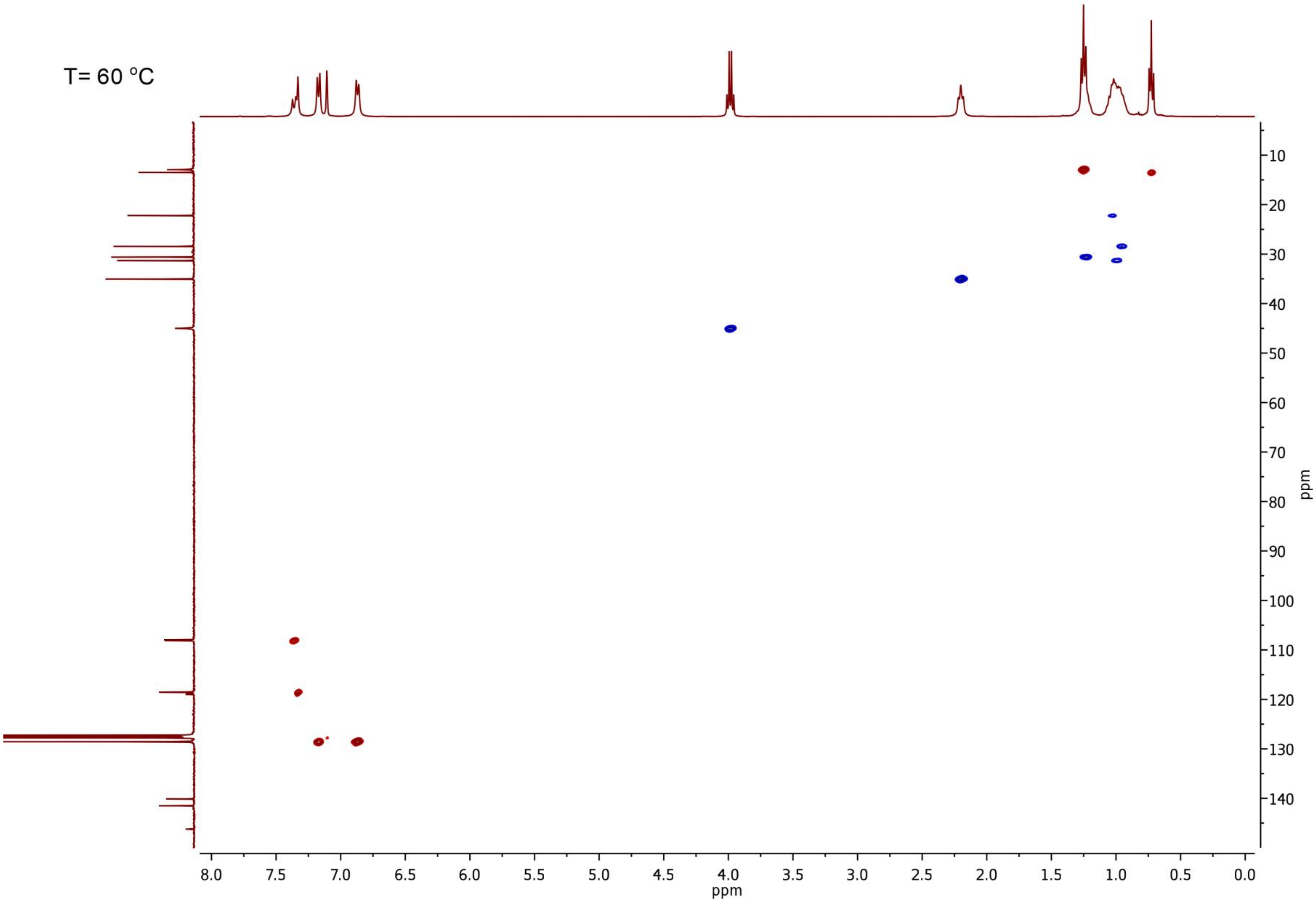

Figure S31. $^{1}H/^{13}C$ HSQC NMR spectrum in C_6D_6 at 60ºC

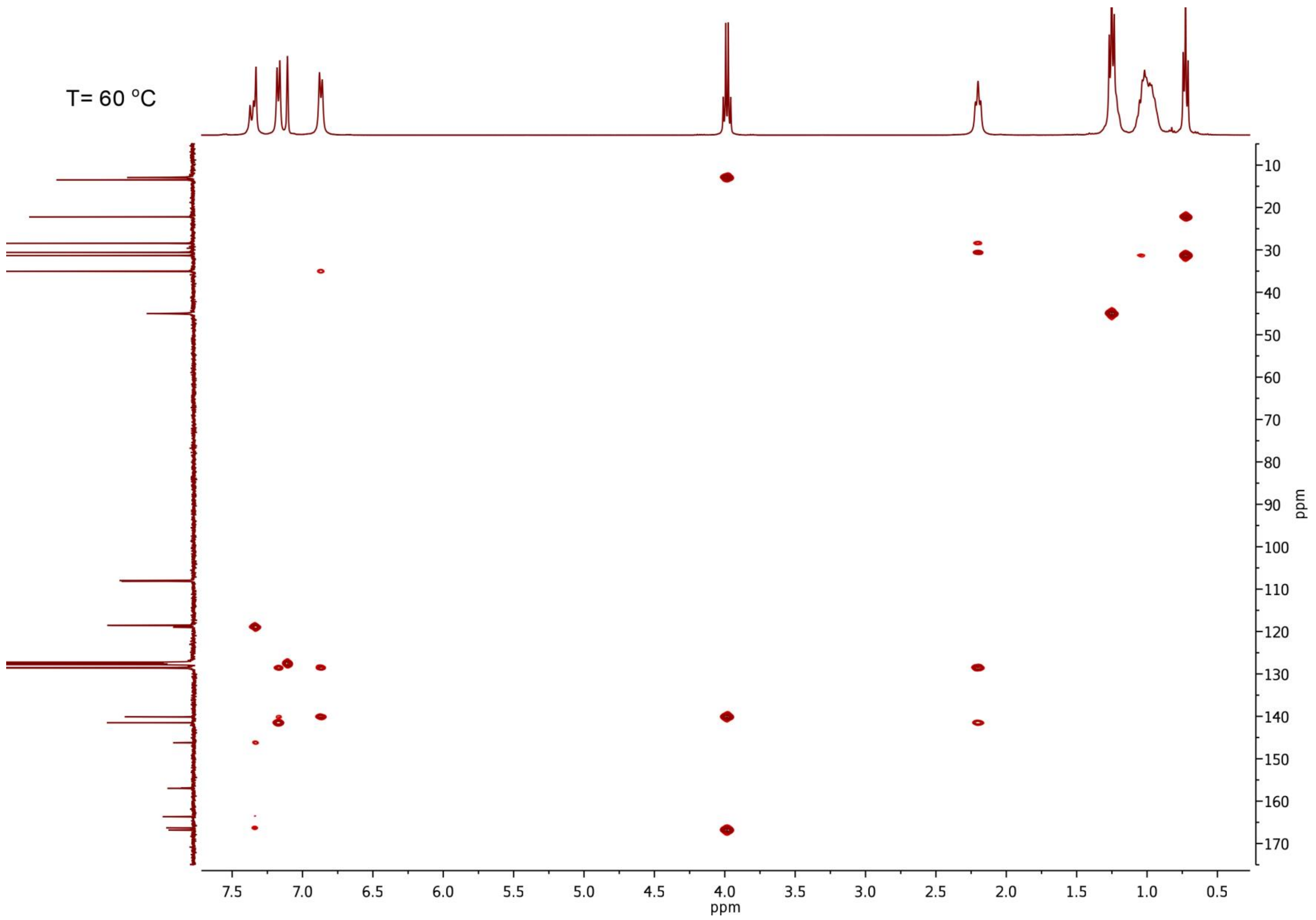

Figure S32. $^{1}H/^{13}C$ HMBC NMR spectrum in C_6D_6 at 60ºC

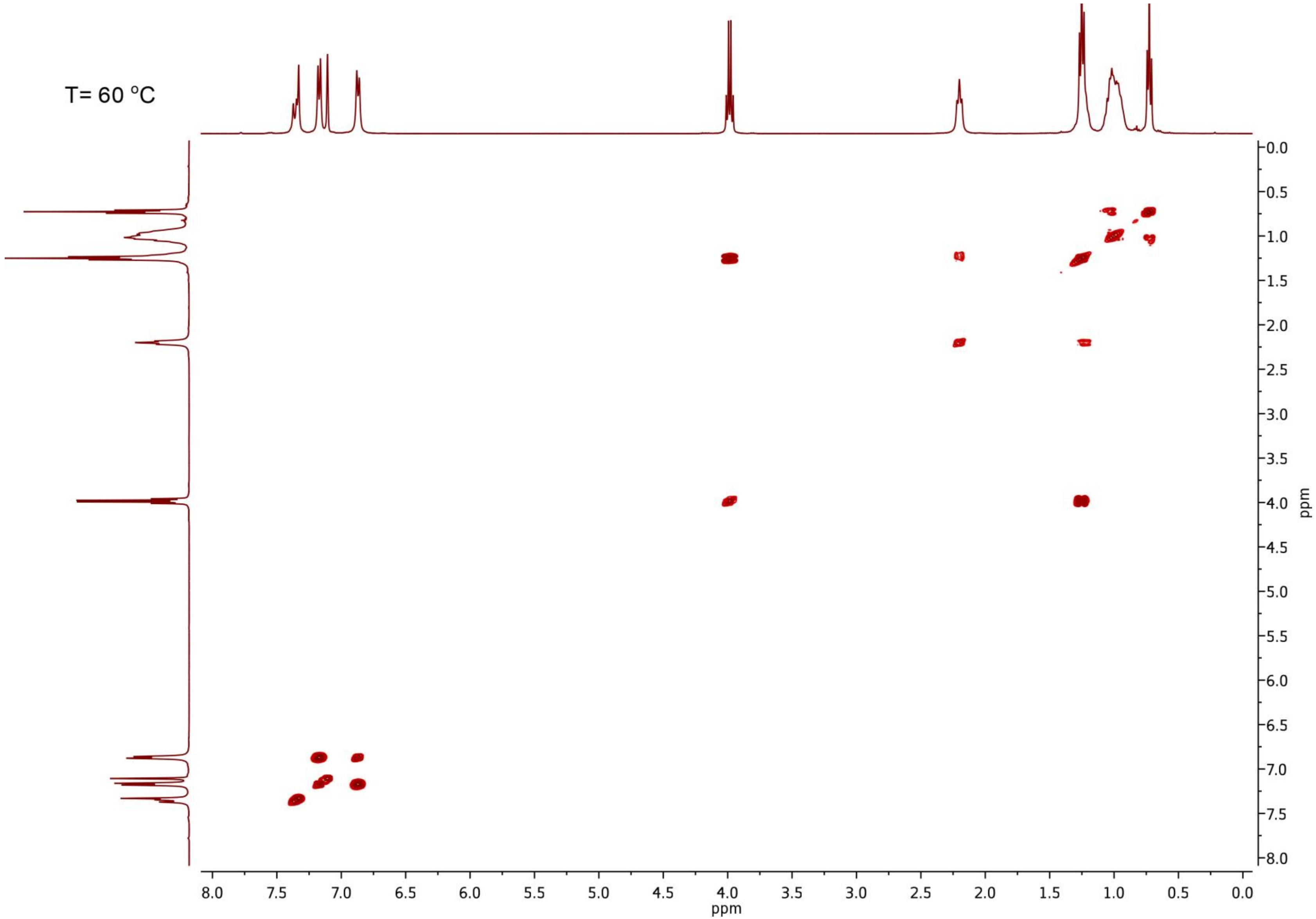

Figure S33. $^{1}H/^{1}H$ COSY NMR spectrum in C_6D_6 at 60ºC

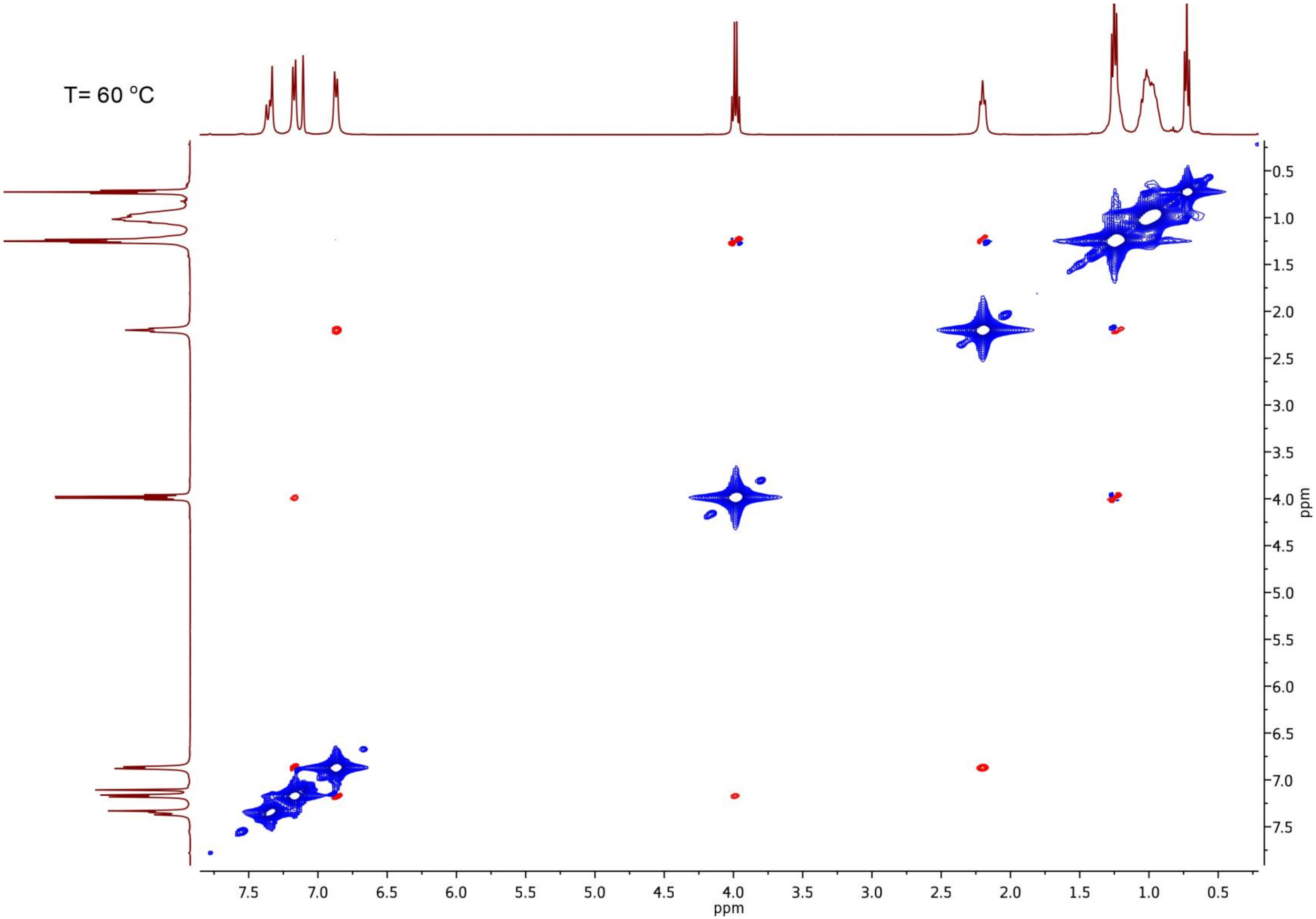

Figure S34. $^{1}H/^{1}H$ NOESY NMR spectrum in C_6D_6 at 60ºC

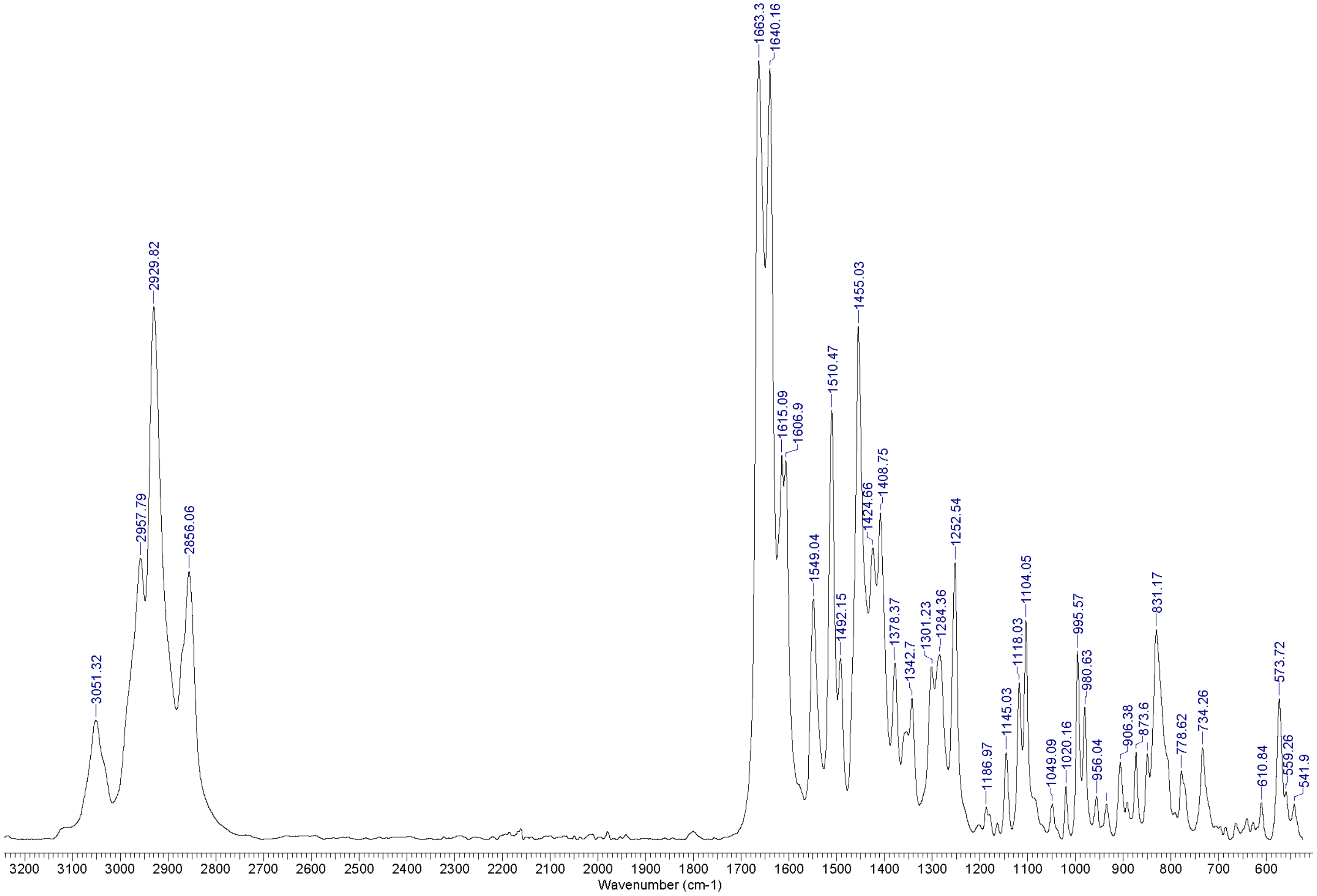

Figure S35. Solid-state IR spectrum at 25ºC

NMR and IR spectra of complexes

Complex 7a*La(NO_3)$_3$

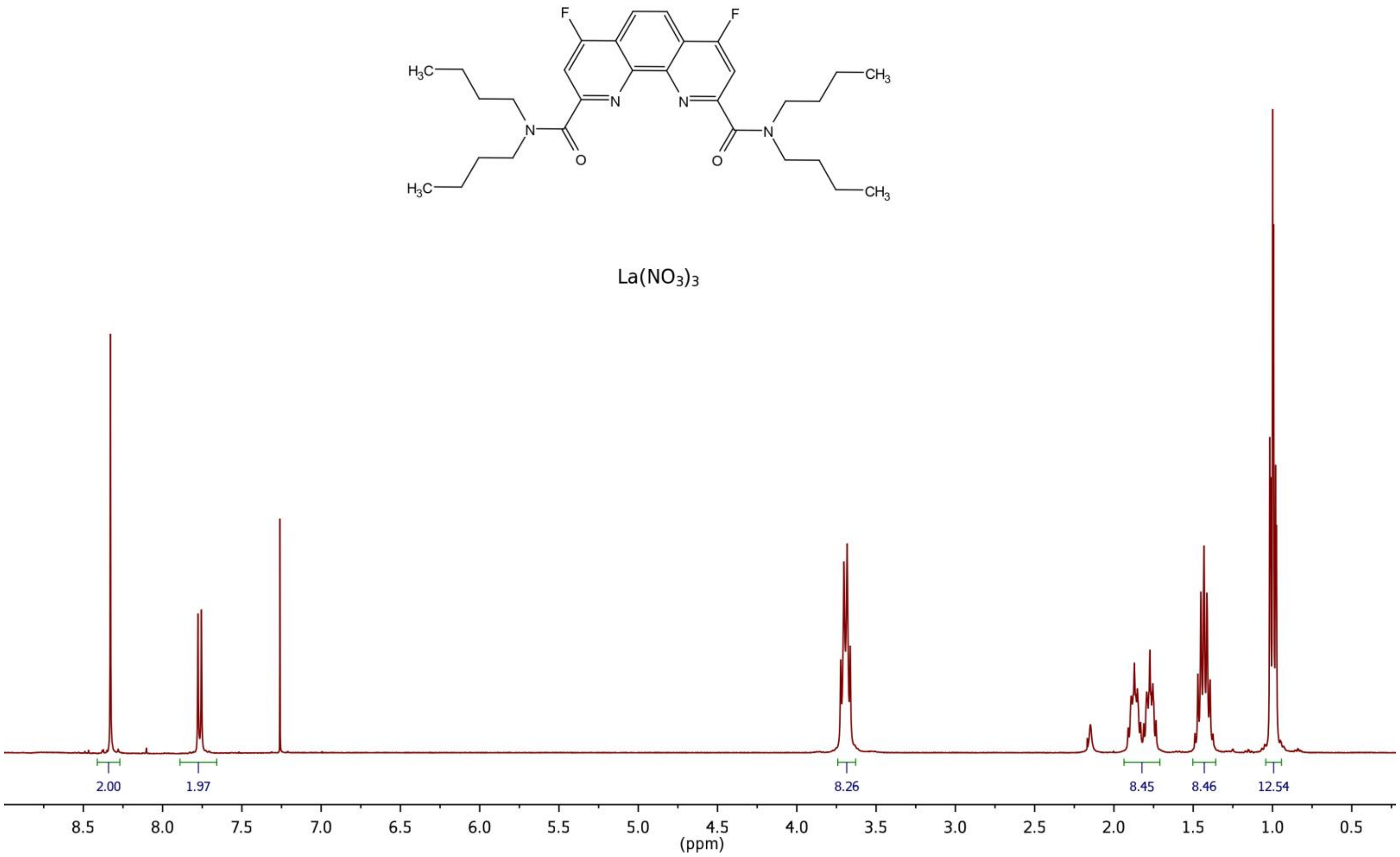

Figure S36. ^{1}H NMR spectrum in $CDCl_3$ at 25ºC

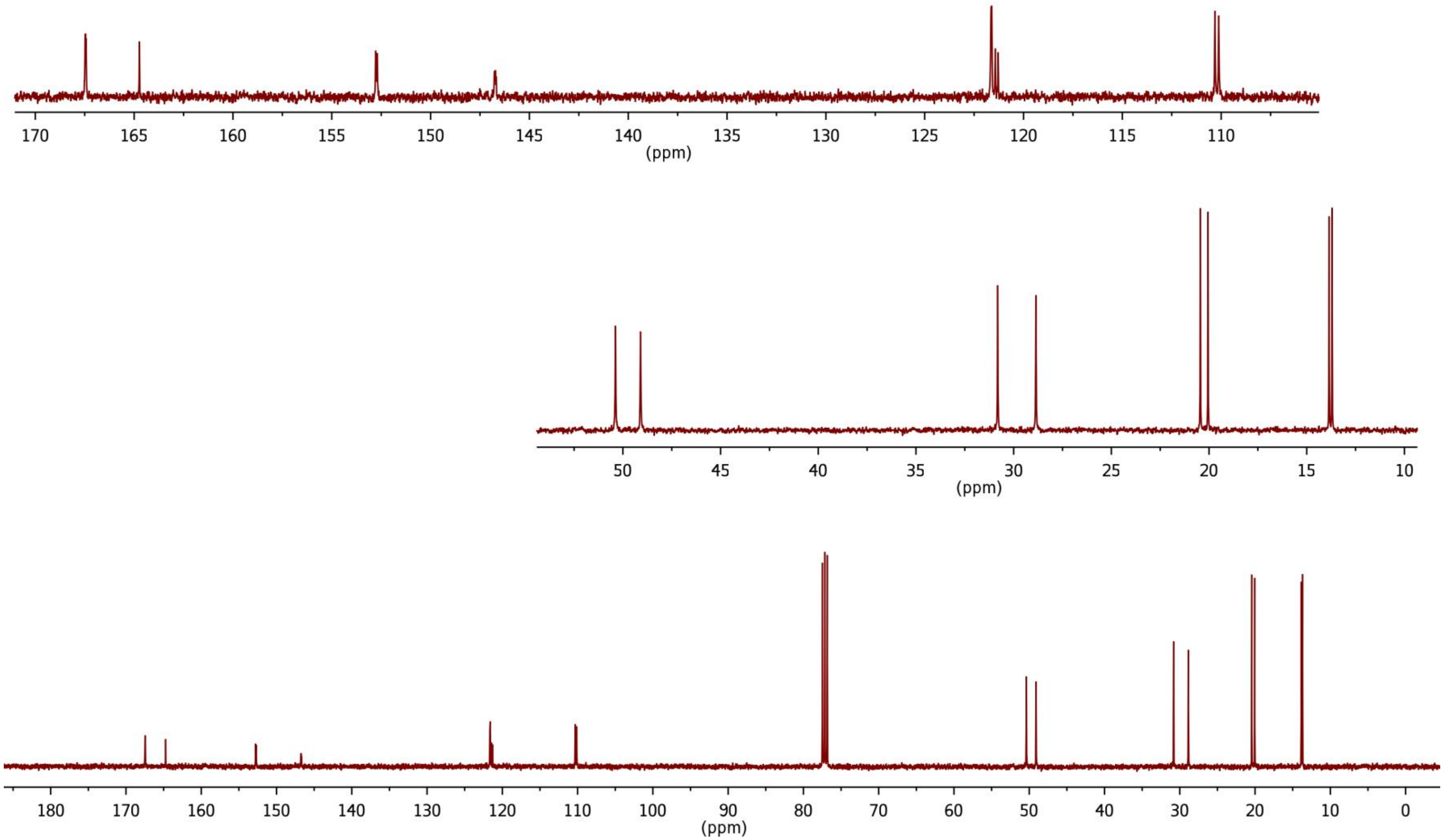

Figure S37. ^{13}C NMR spectrum in $CDCl_3$ at 25ºC

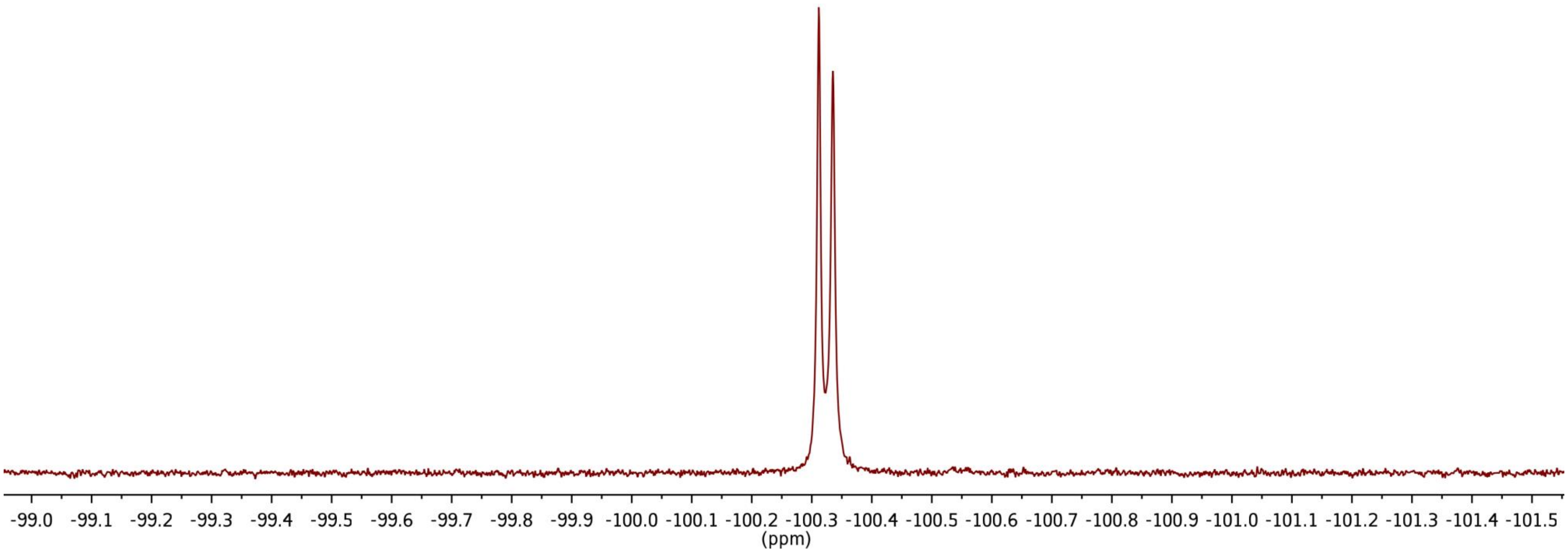

Figure S38. ^{19}F NMR spectrum in $CDCl_3$ at 25ºC

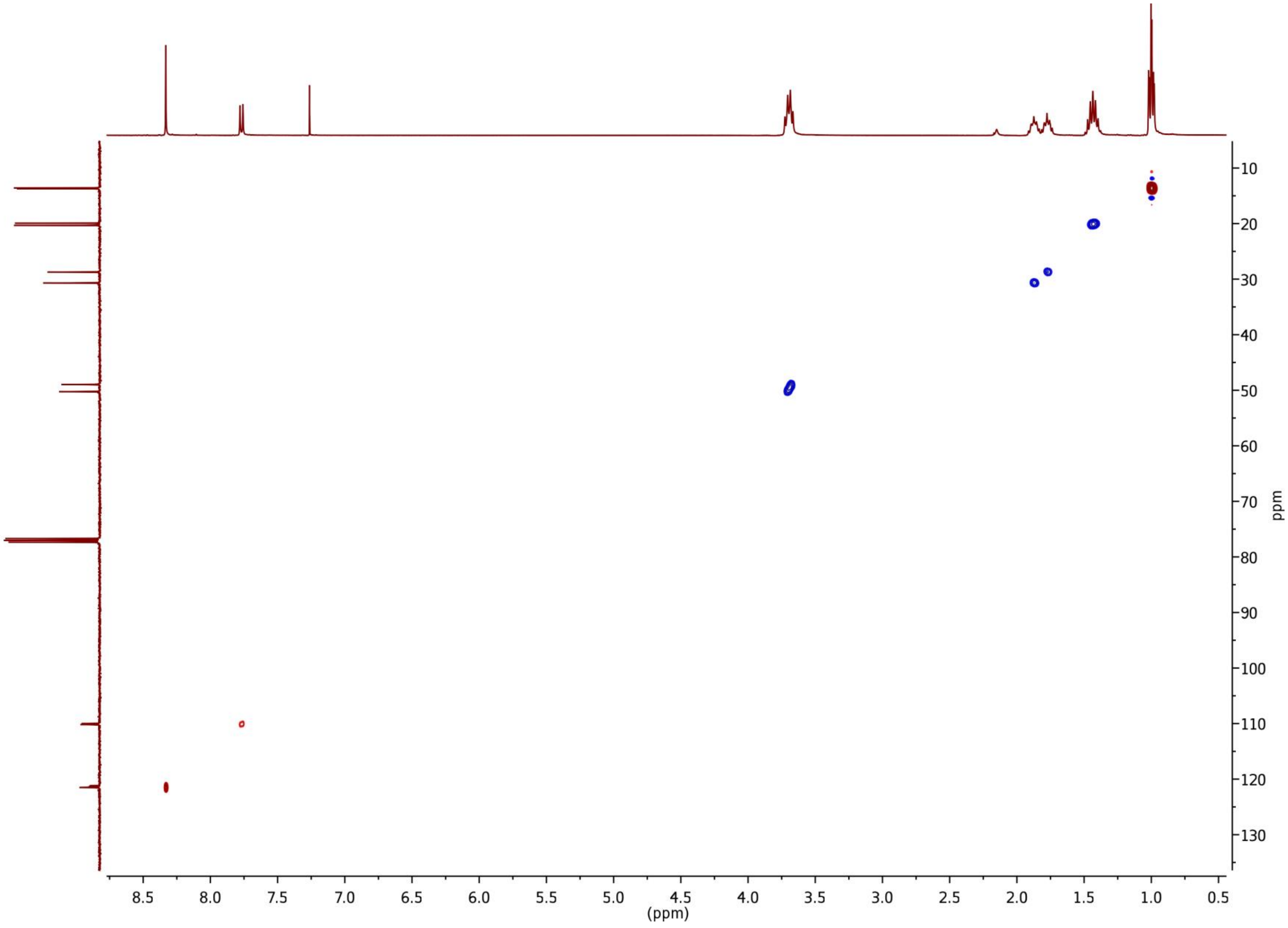

Figure S39. $^{1}H/^{13}C$ HSQC NMR spectrum in $CDCl_3$ at 25ºC

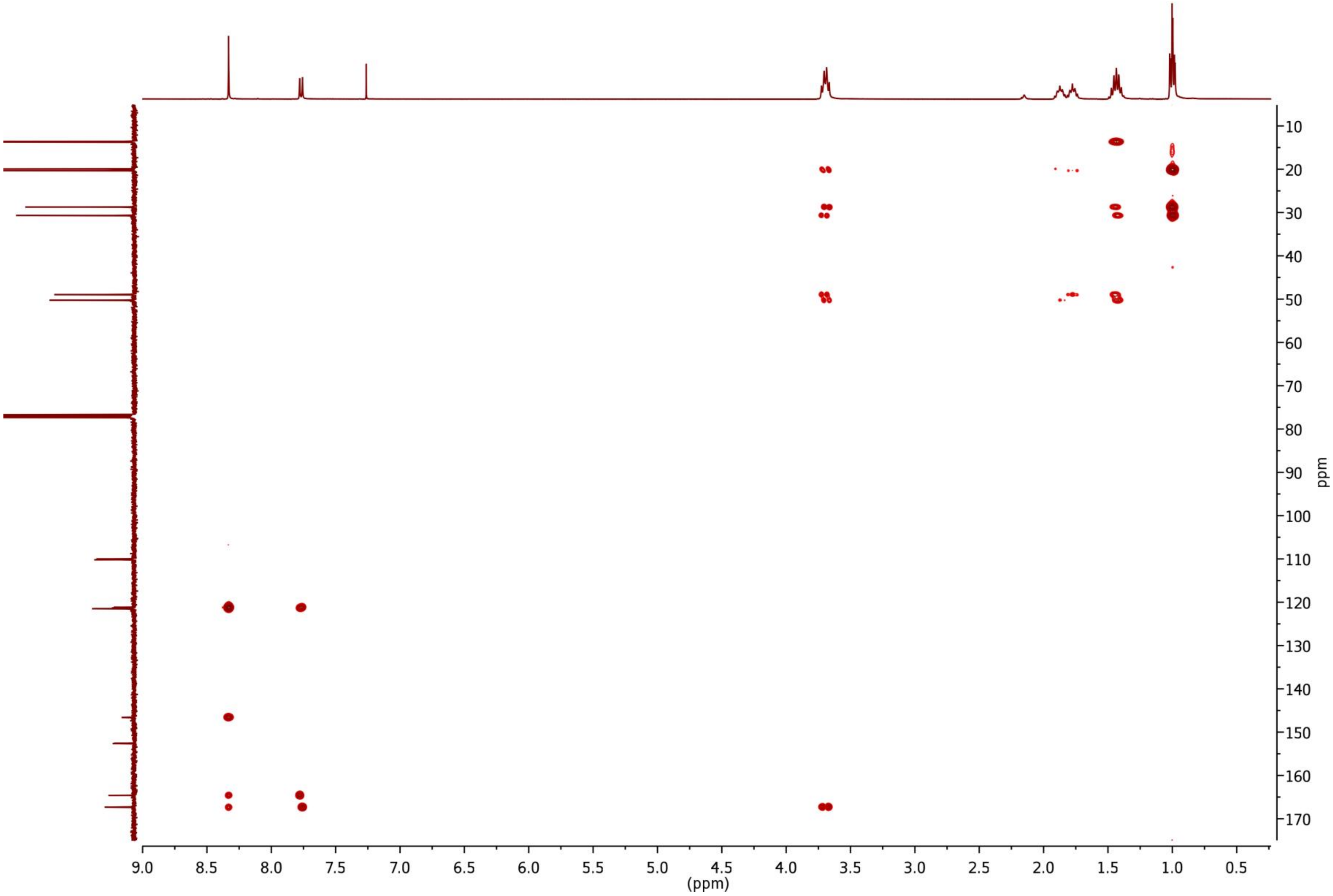

Figure S40. $^{1}H/^{13}C$ HMBC NMR spectrum in $CDCl_3$ at 25ºC

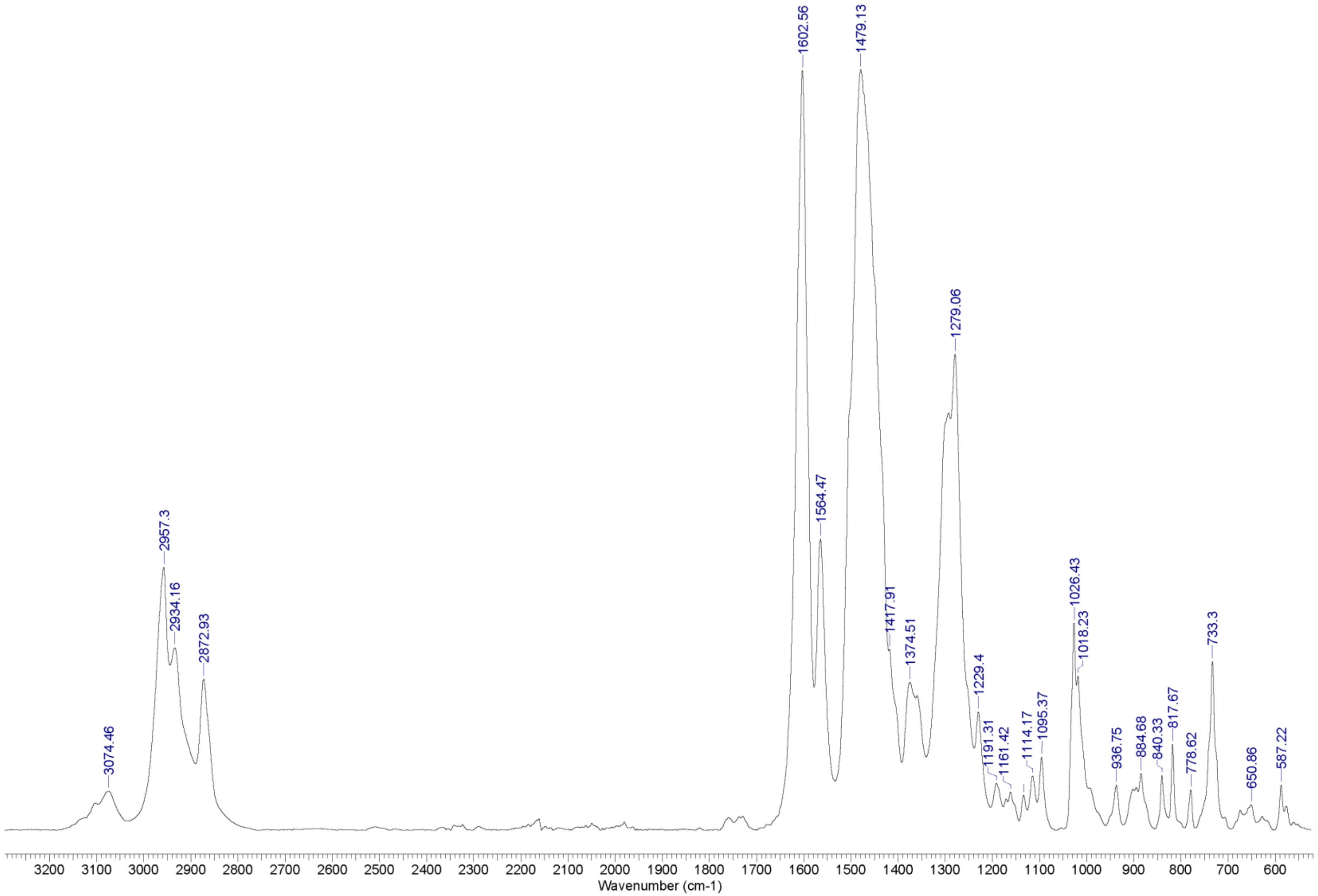

Figure S41. Solid-state IR spectrum at 25ºC

Complex 7a*Nd(NO_3)$_3$

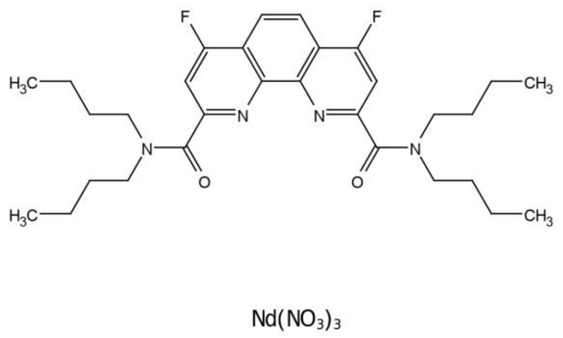

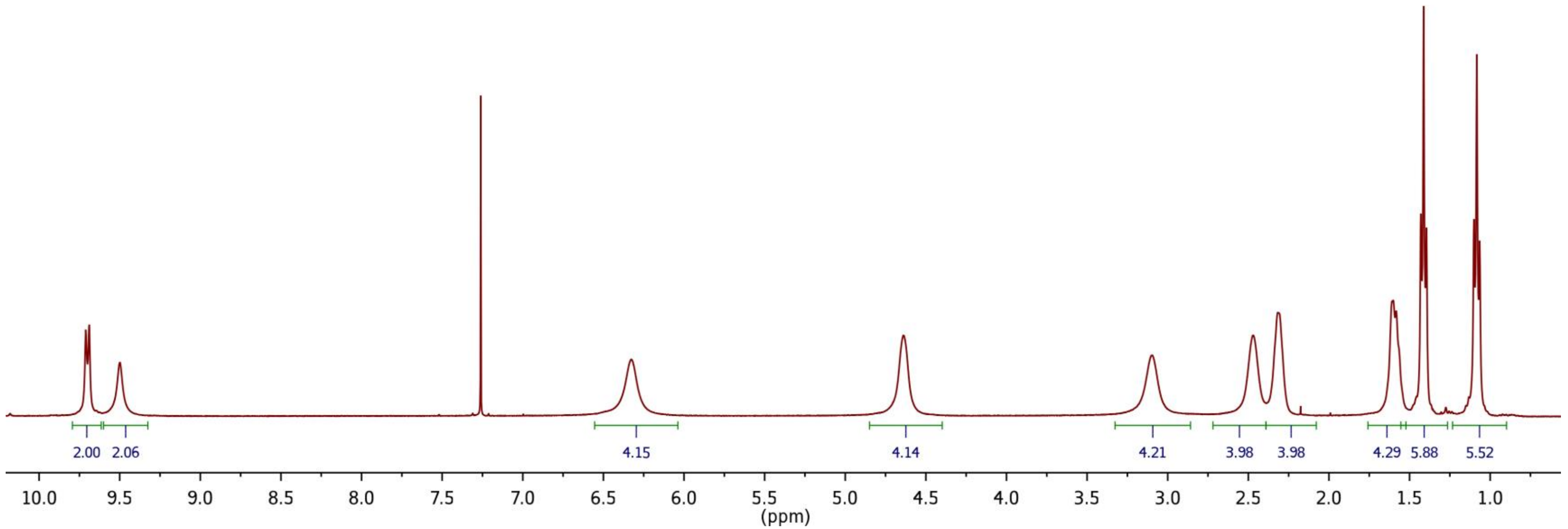

Figure S42. ^{1}H NMR spectrum in $CDCl_3$ at 25ºC

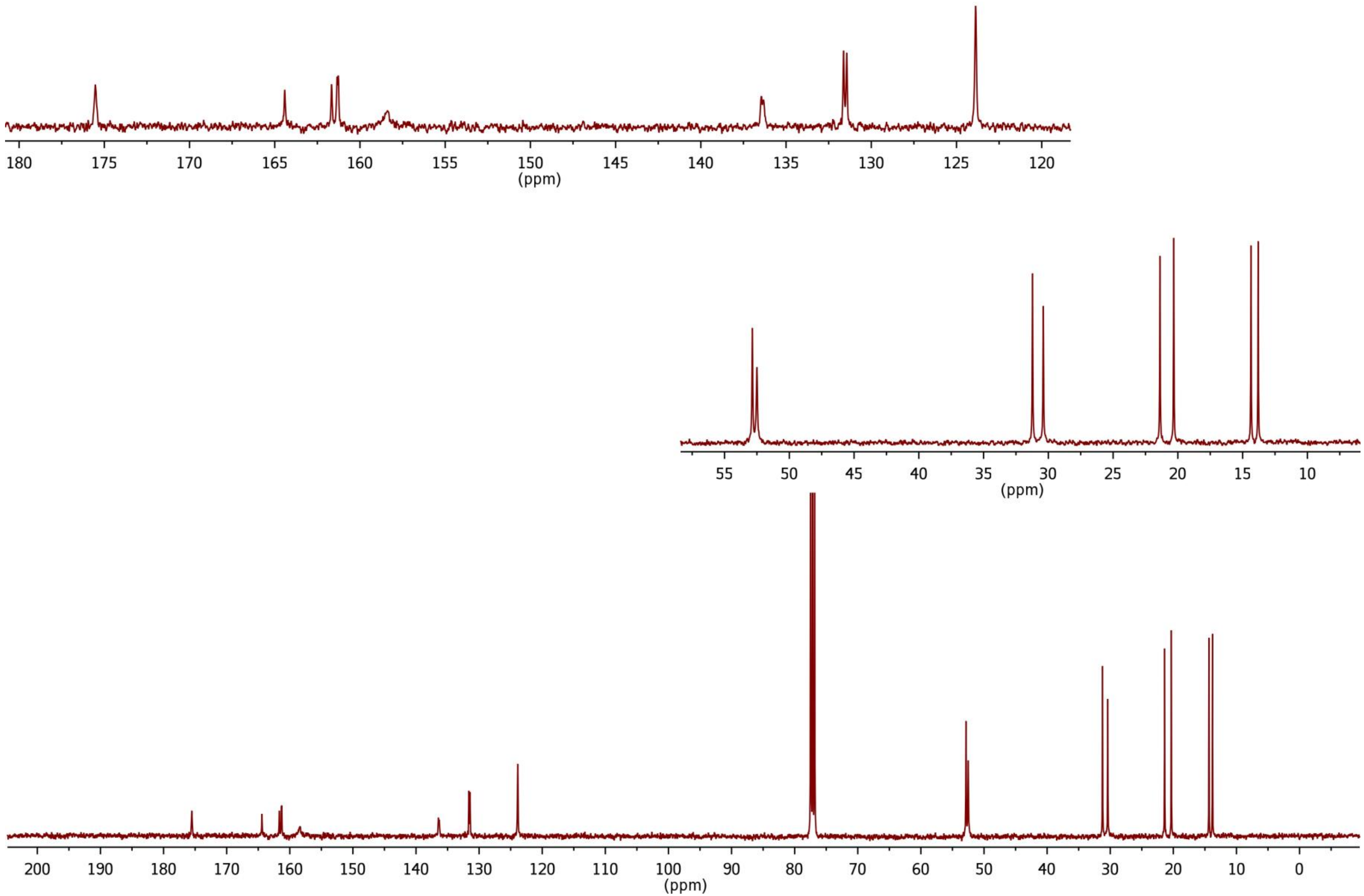

Figure S43. ^{13}C NMR spectrum in $CDCl_3$ at 25ºC

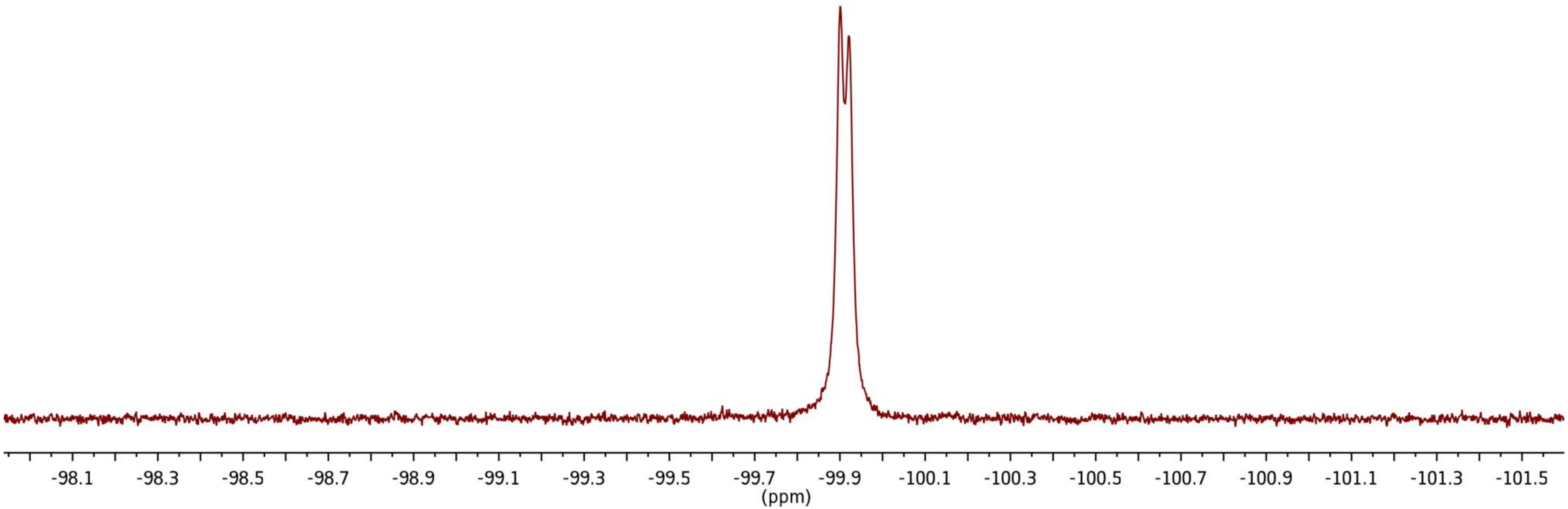

Figure S44. ^{19}F NMR spectrum in $CDCl_3$ at 25ºC

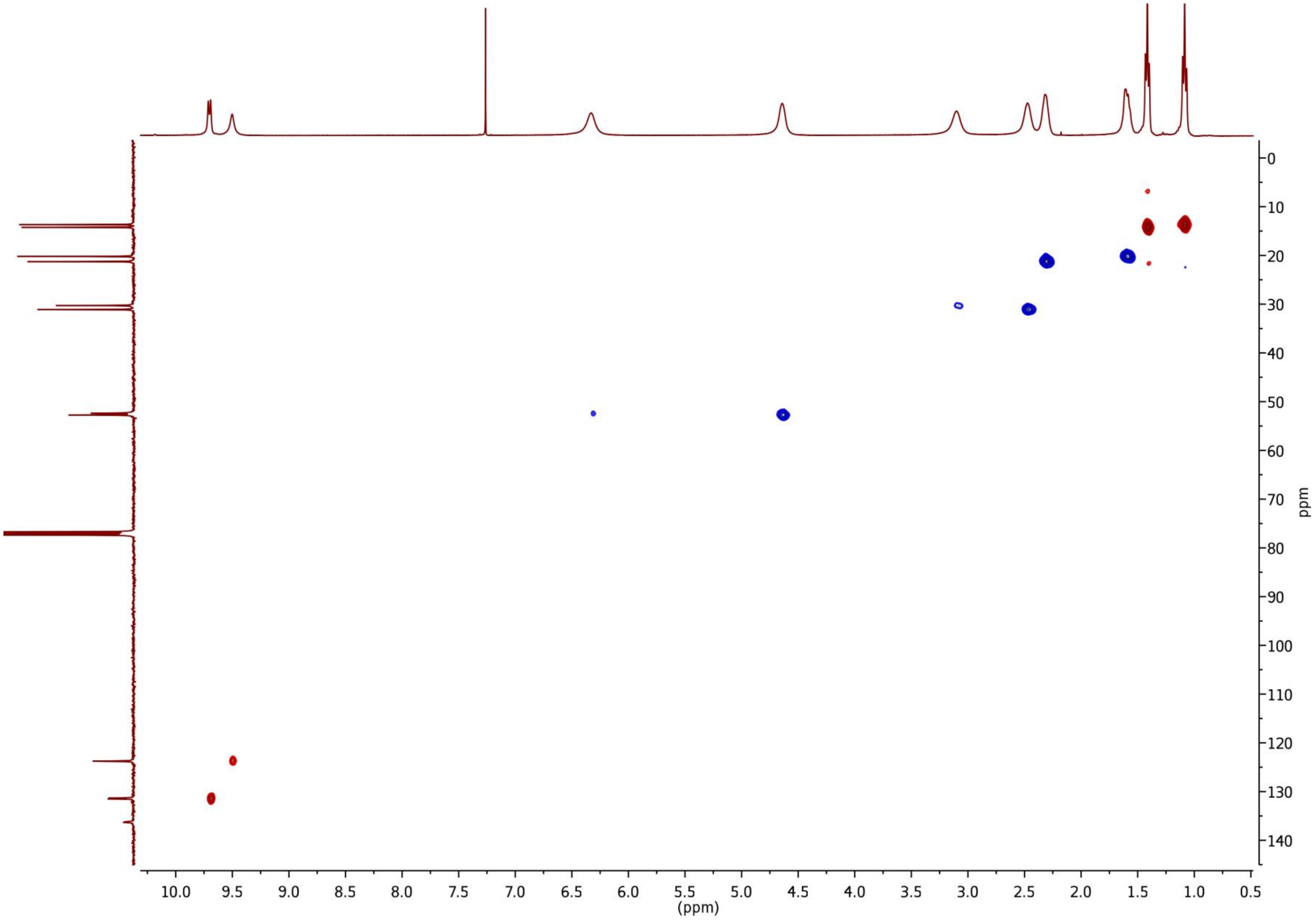

Figure S45. $^{1}H/^{13}C$ HSQC NMR spectrum in $CDCl_3$ at 25ºC

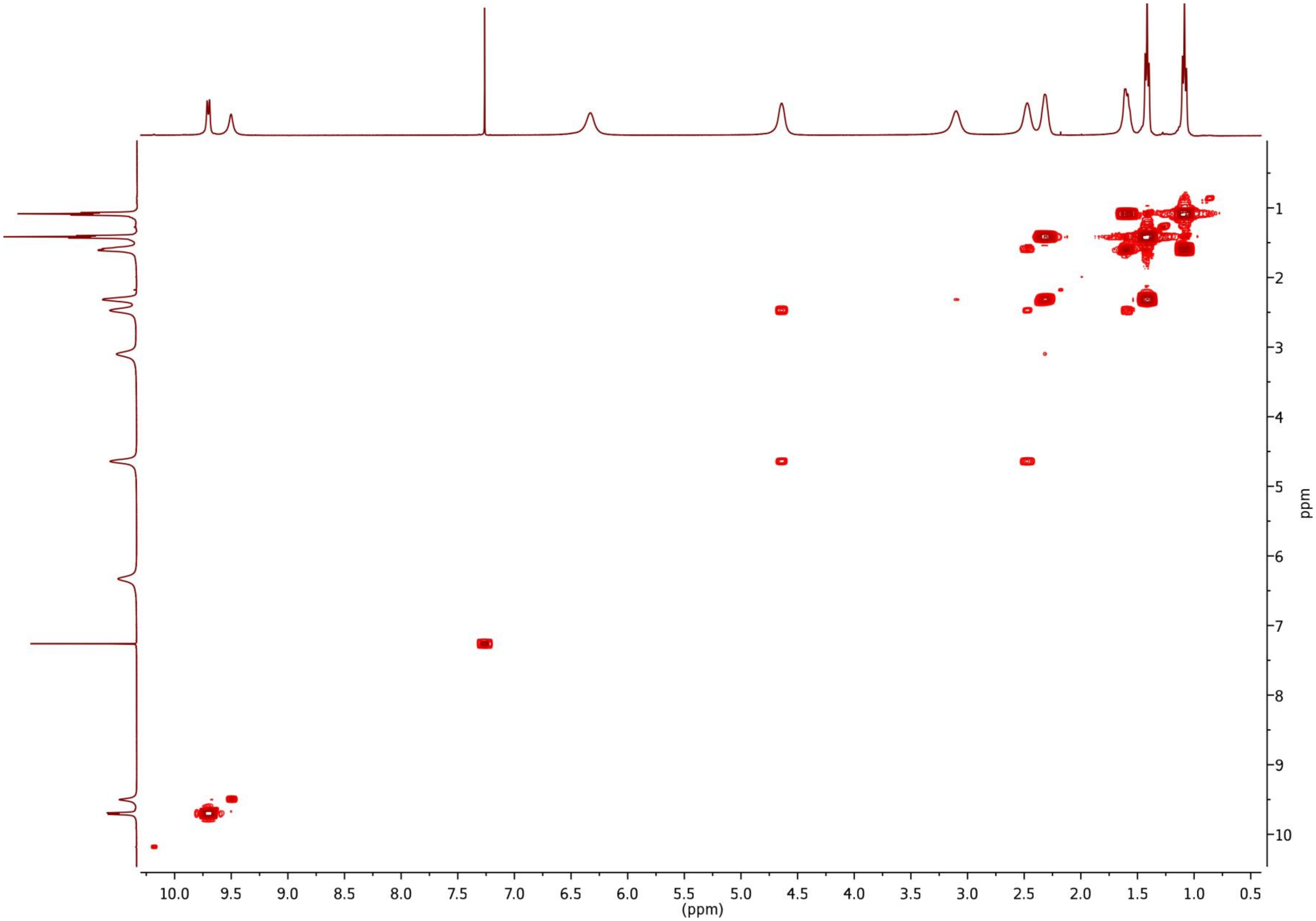

Figure S46. $^{1}H/^{13}C$ HMBC NMR spectrum in $CDCl_3$ at 25ºC

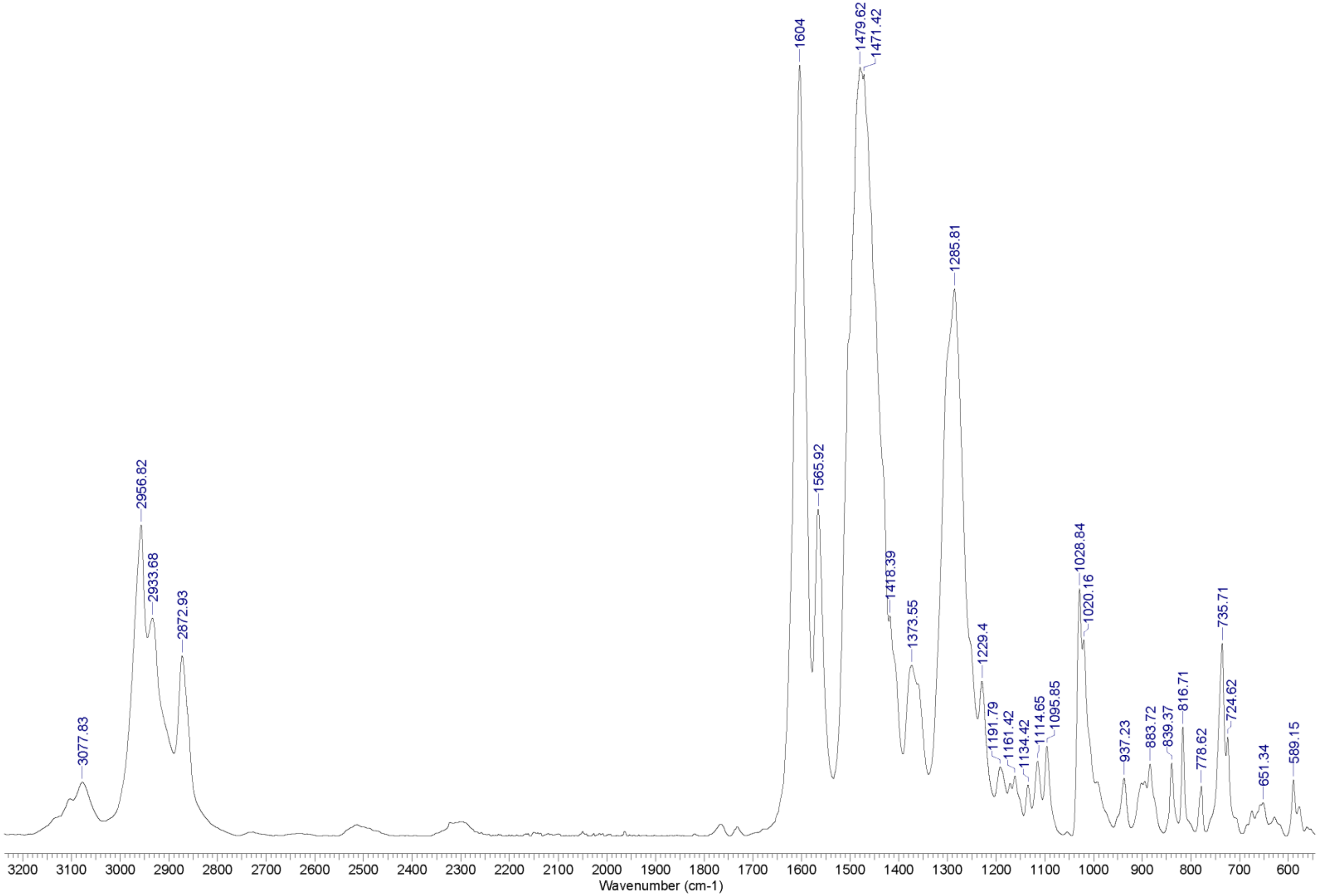

Figure S47. Solid-state IR spectrum at 25ºC

Complex 7a*Eu(NO3)3

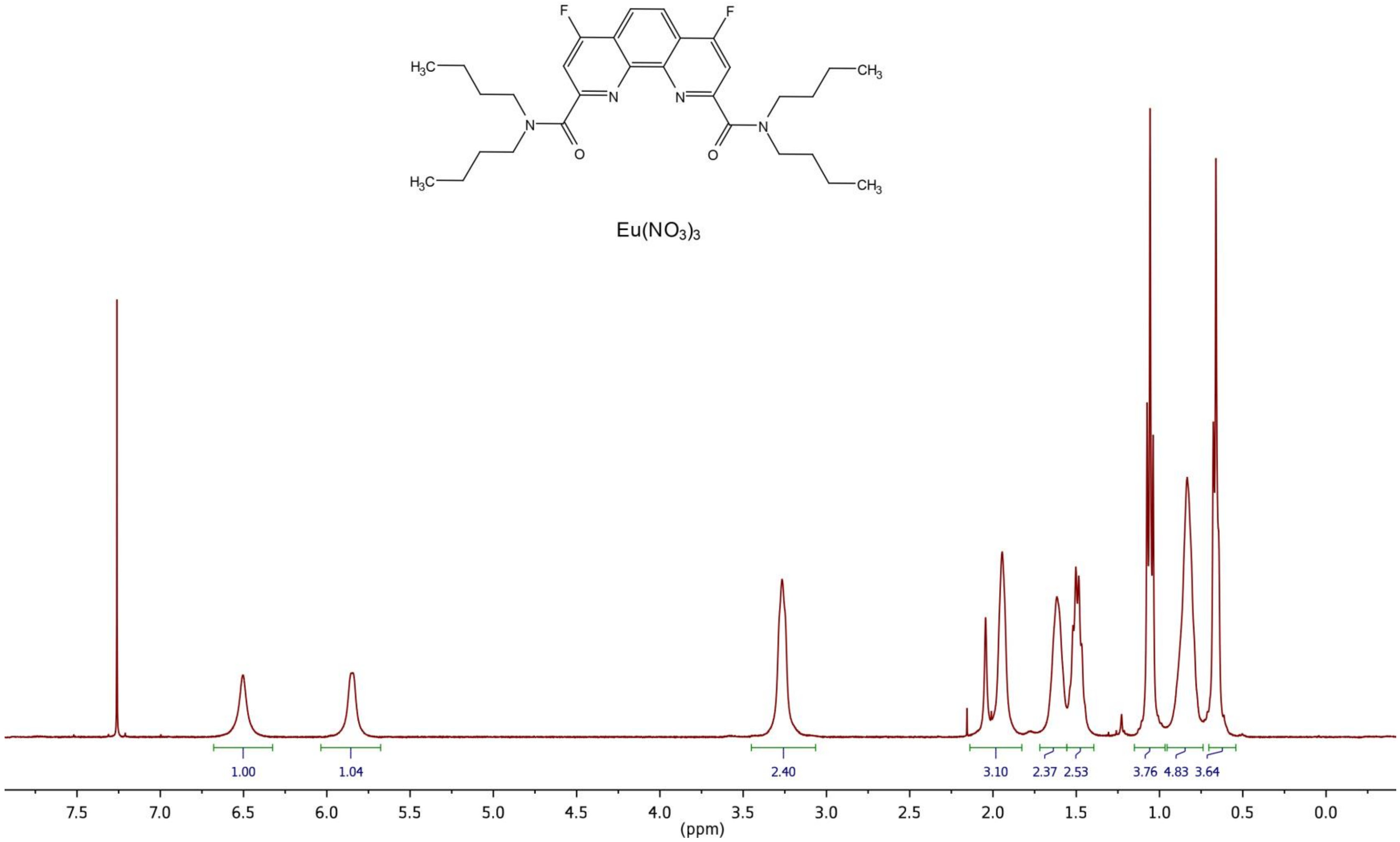

Figure S48. ^{1}H NMR spectrum in $CDCl_3$ at 25ºC

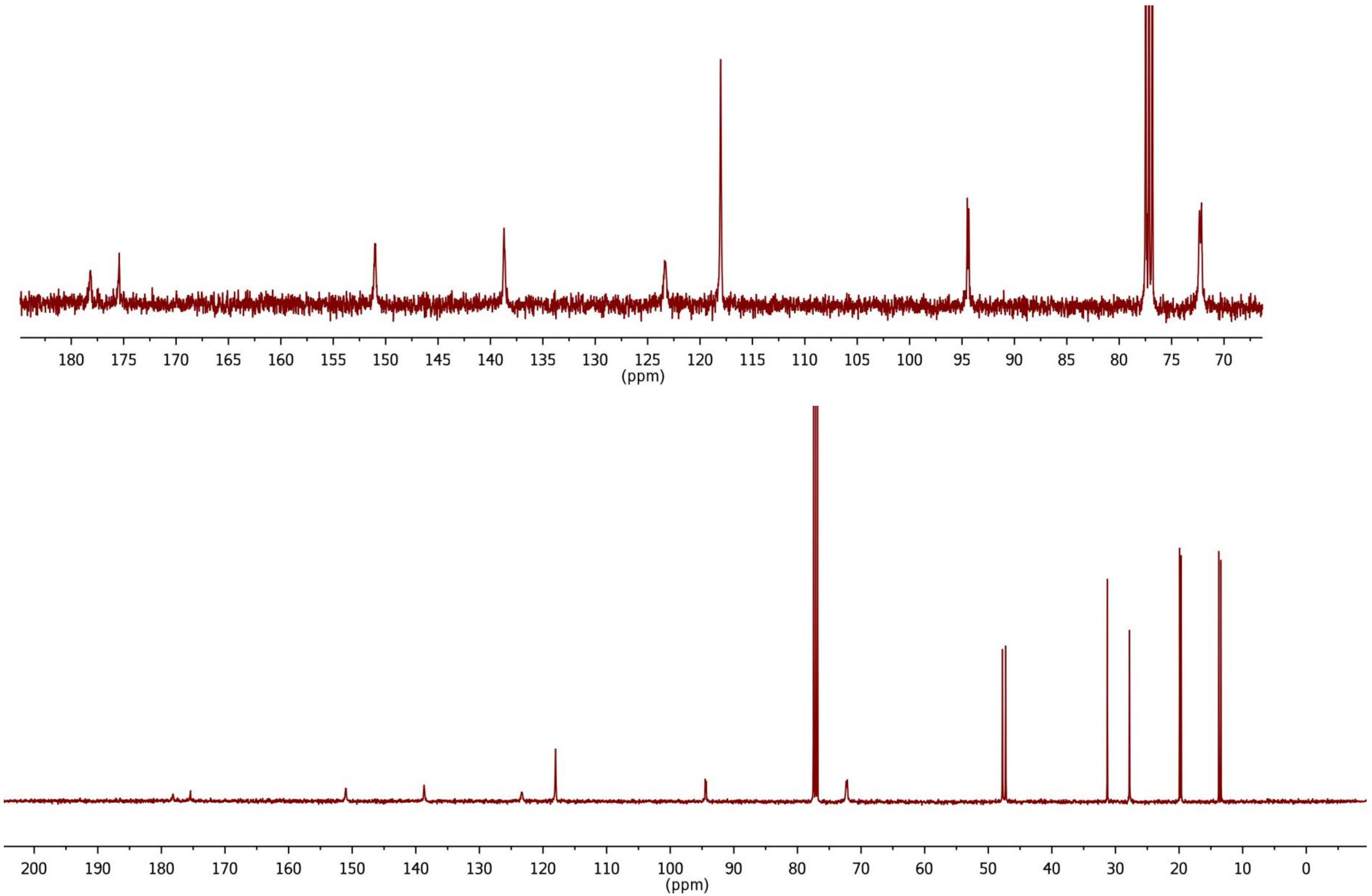

Figure S49. ^{13}C NMR spectrum in $CDCl_3$ at 25ºC

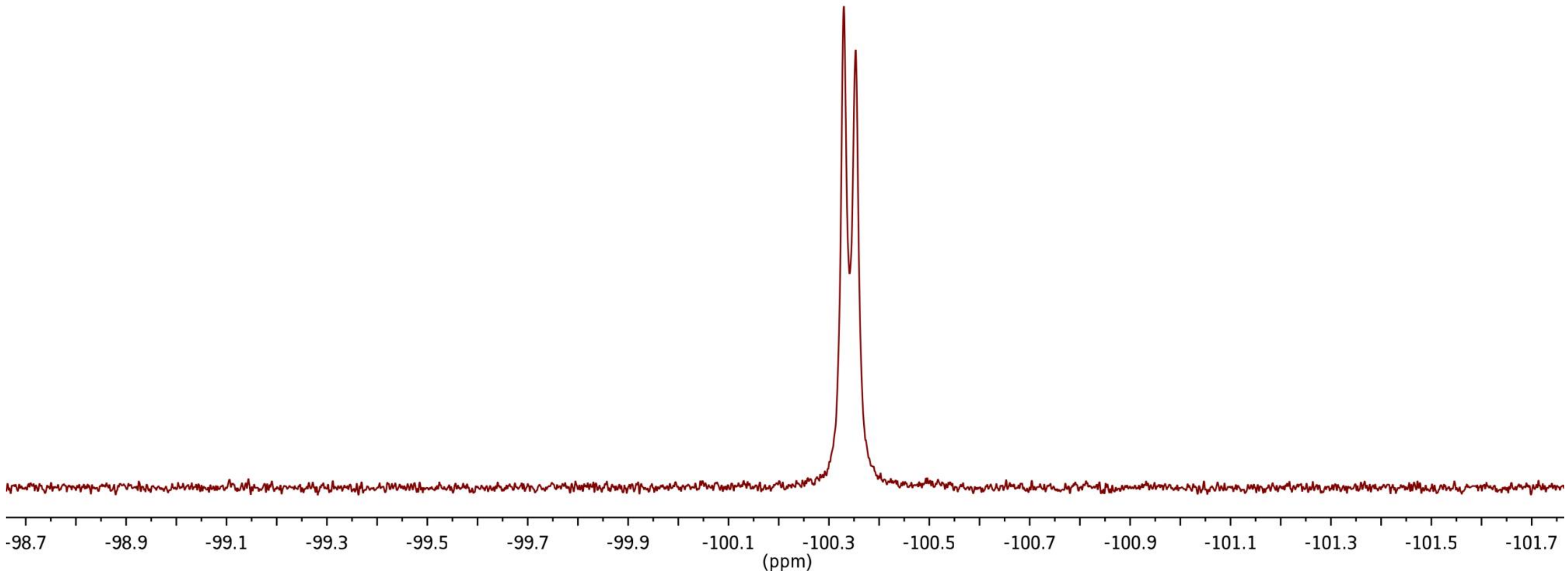

Figure S50. ^{19}F NMR spectrum in $CDCl_3$ at 25ºC

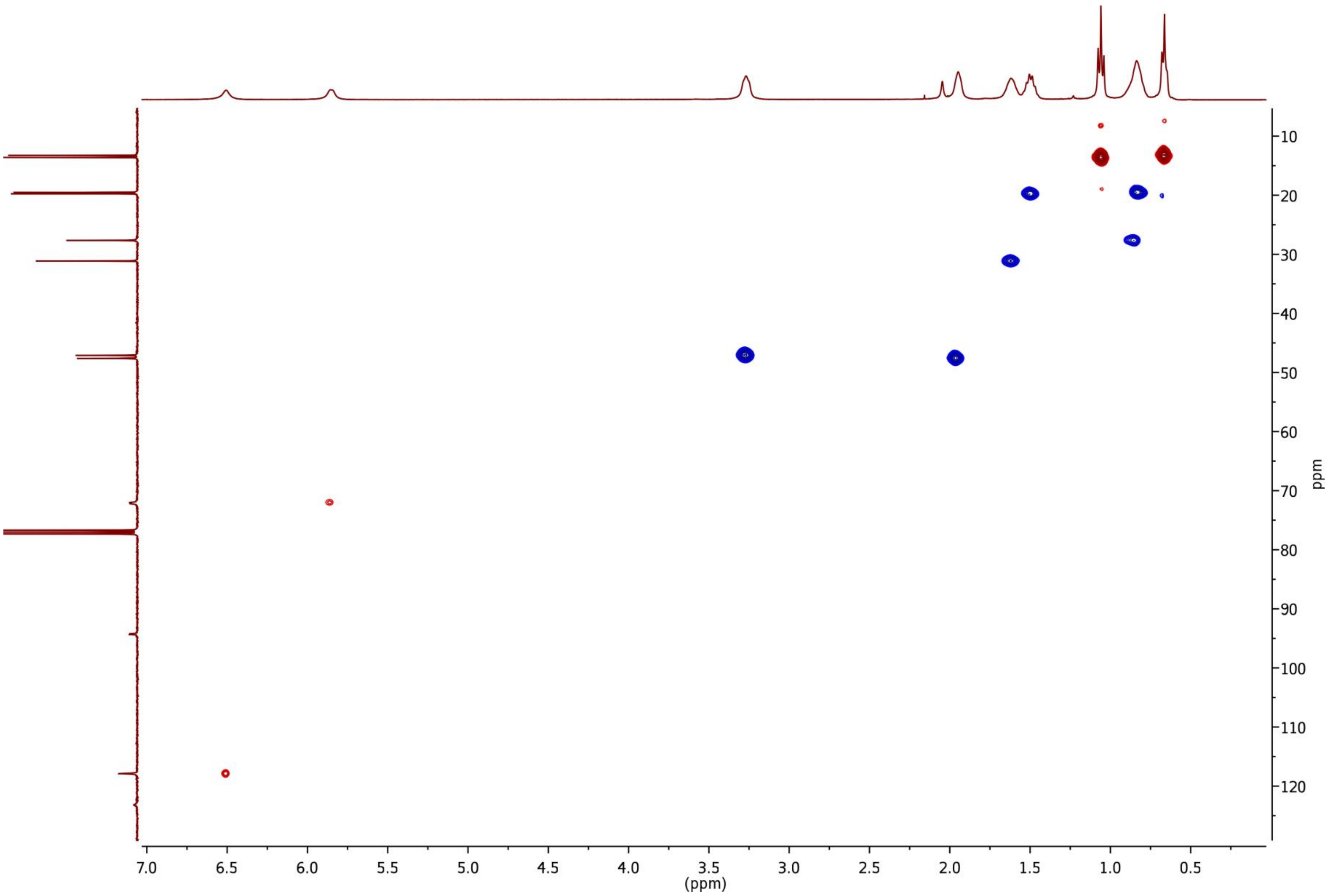

Figure S51. $^{1}H/^{13}C$ HSQC NMR spectrum in $CDCl_3$ at 25ºC

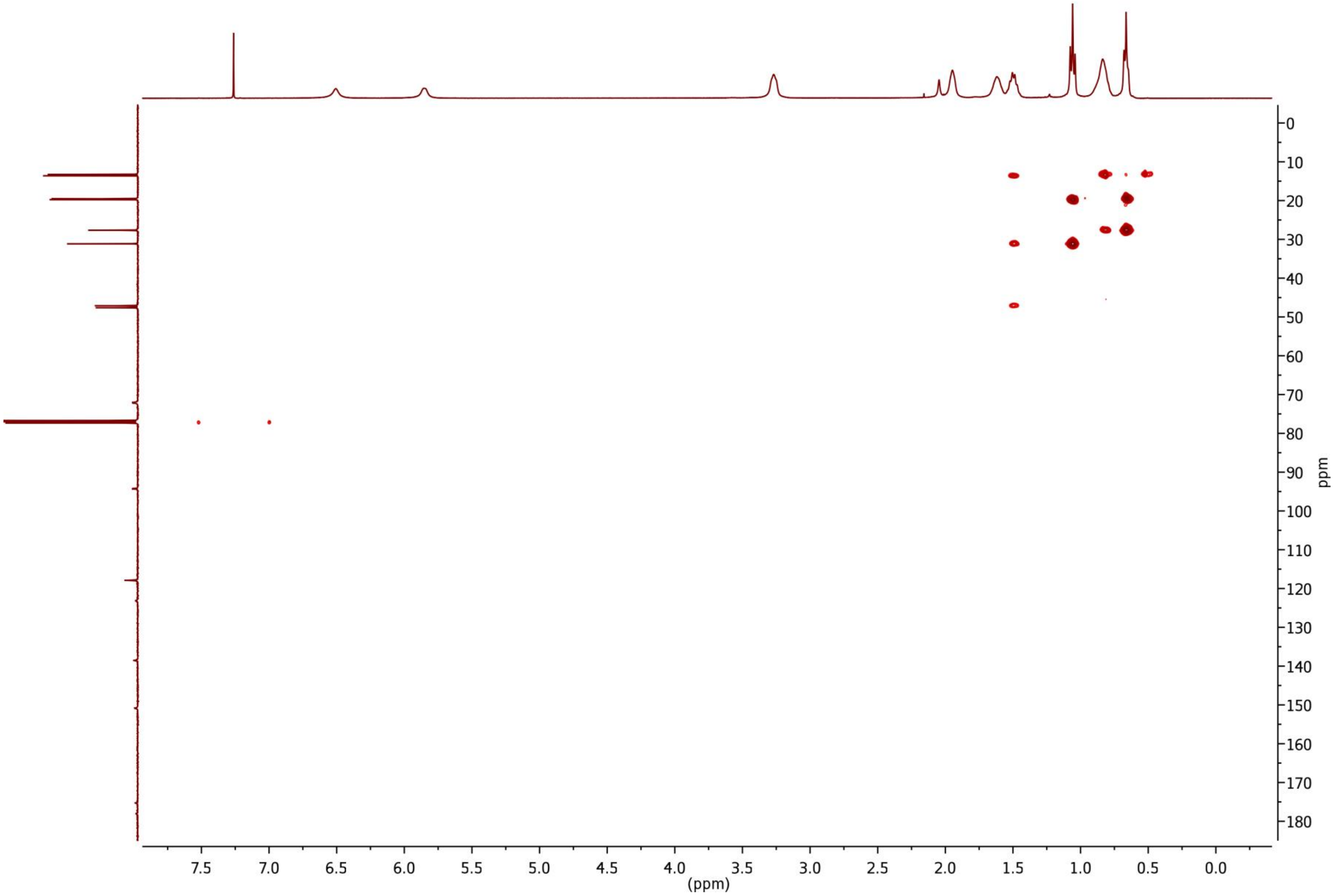

Figure S52. $^{1}H/^{13}C$ HMBC NMR spectrum in $CDCl_3$ at 25ºC

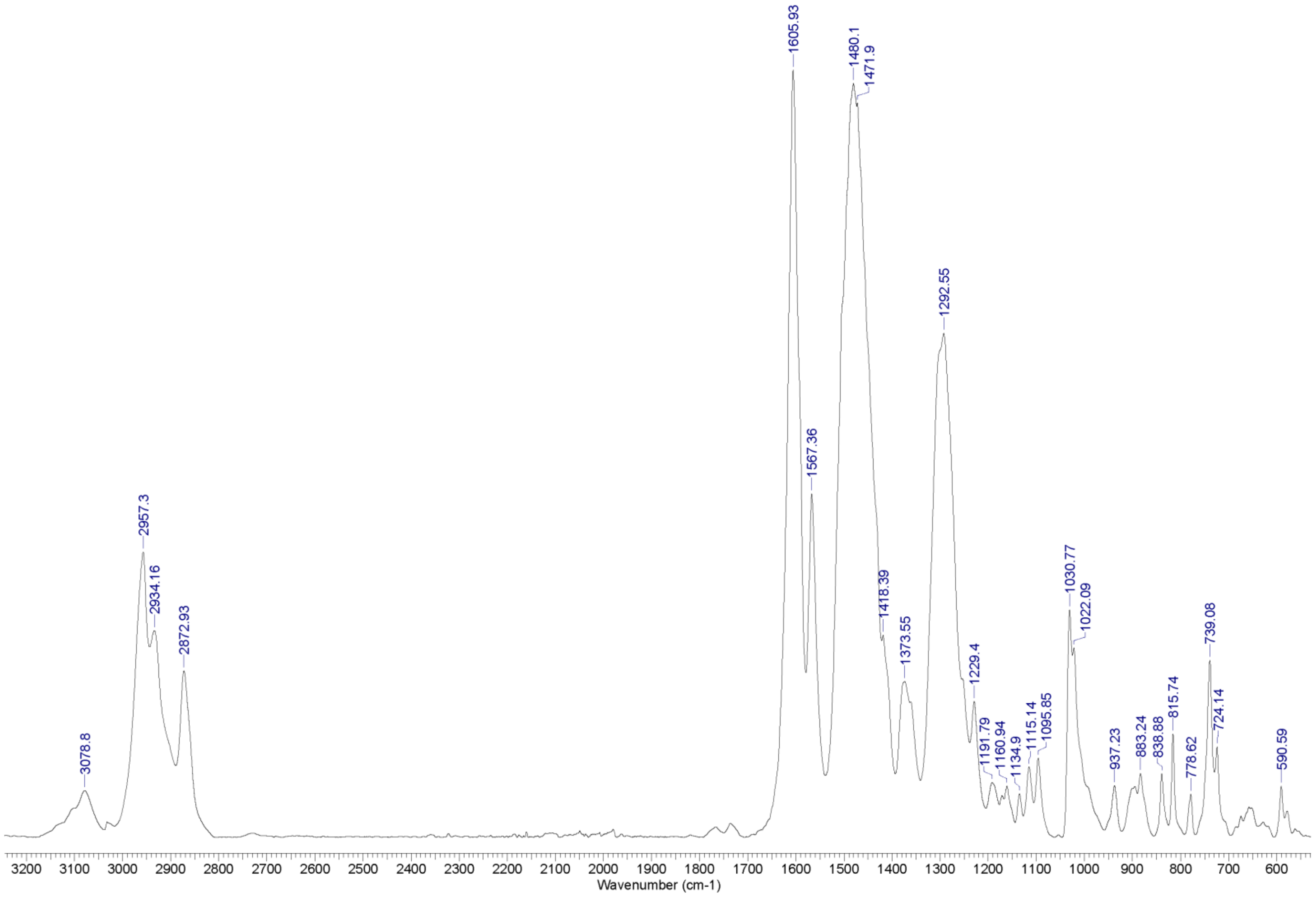

Figure S53. Solid-state IR spectrum at 25ºC

Complex 7a*Lu(NO3)3

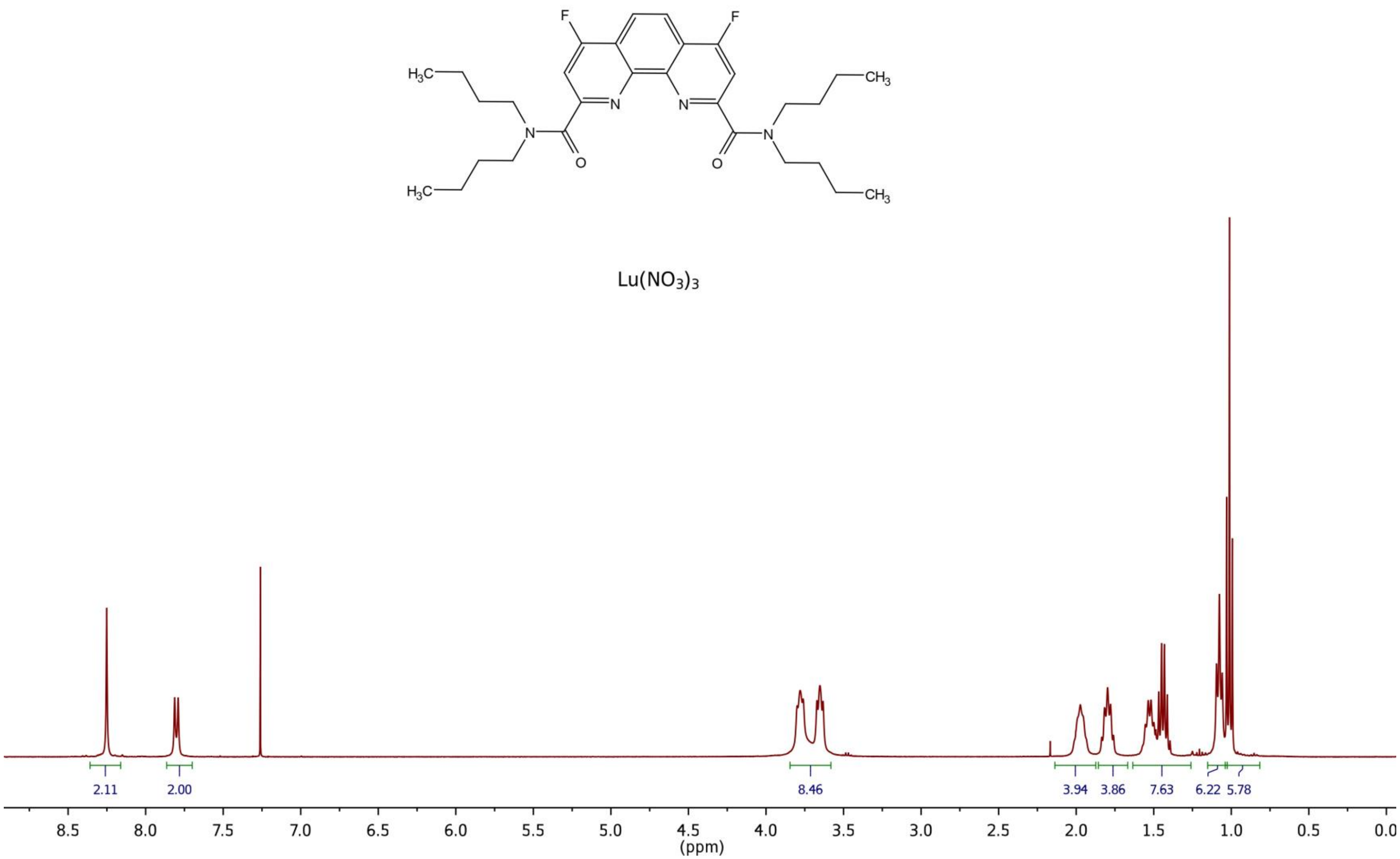

Figure S54. ^{1}H NMR spectrum in $CDCl_3$ at 25ºC

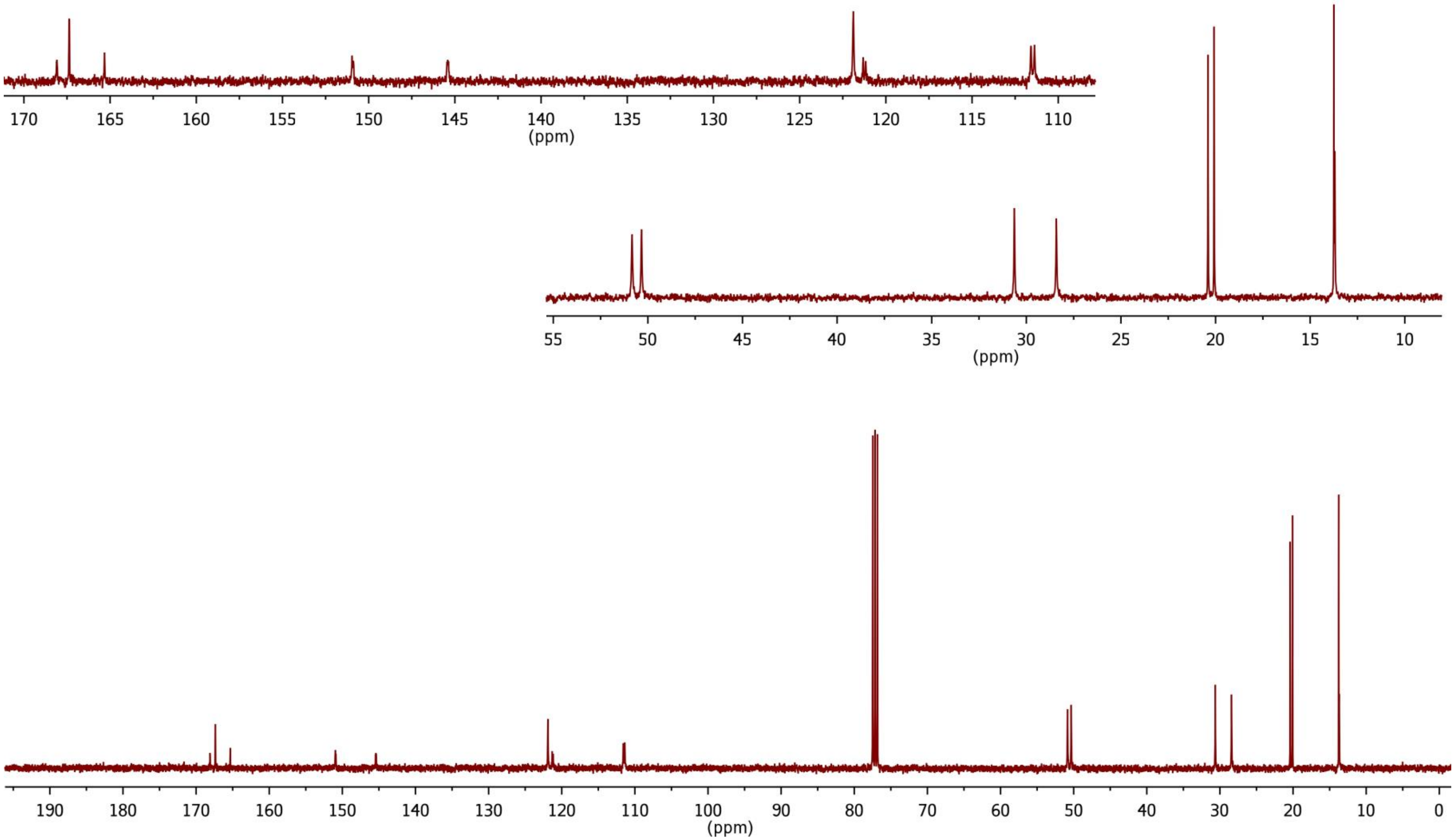

Figure S55. ^{13}C NMR spectrum in in $CDCl_3$ at 25ºC

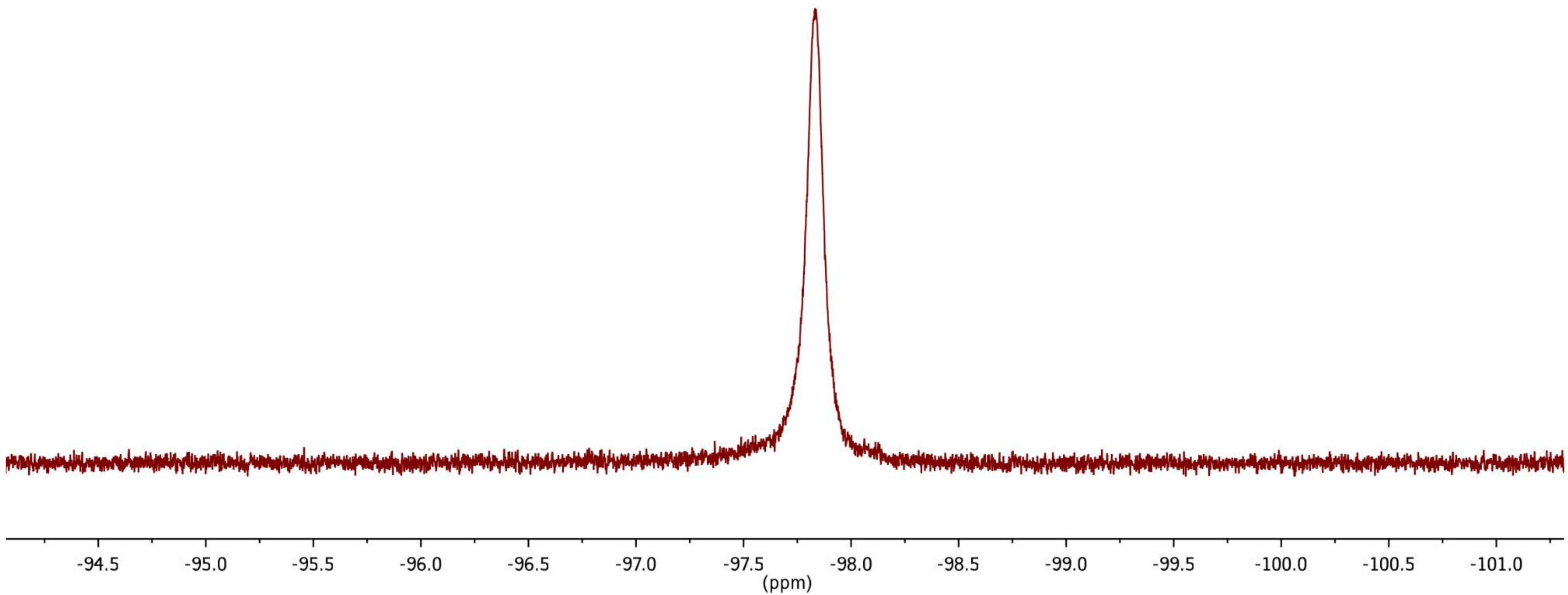

Figure S56. ^{19}F NMR spectrum in $CDCl_3$ at 25ºC

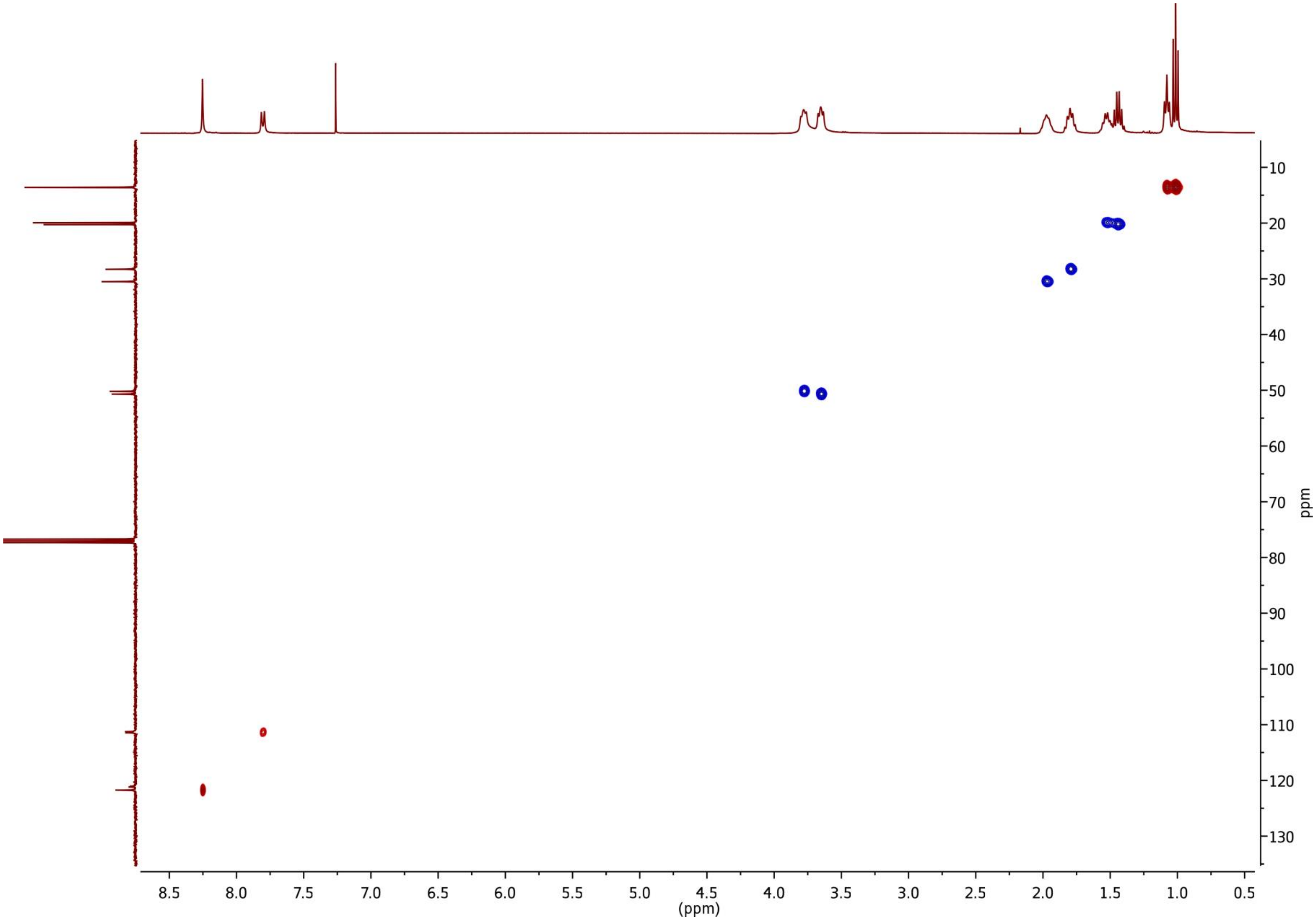

Figure S57. $^1H/^{13}C$ HSQC NMR spectrum in $CDCl_3$ at 25ºC

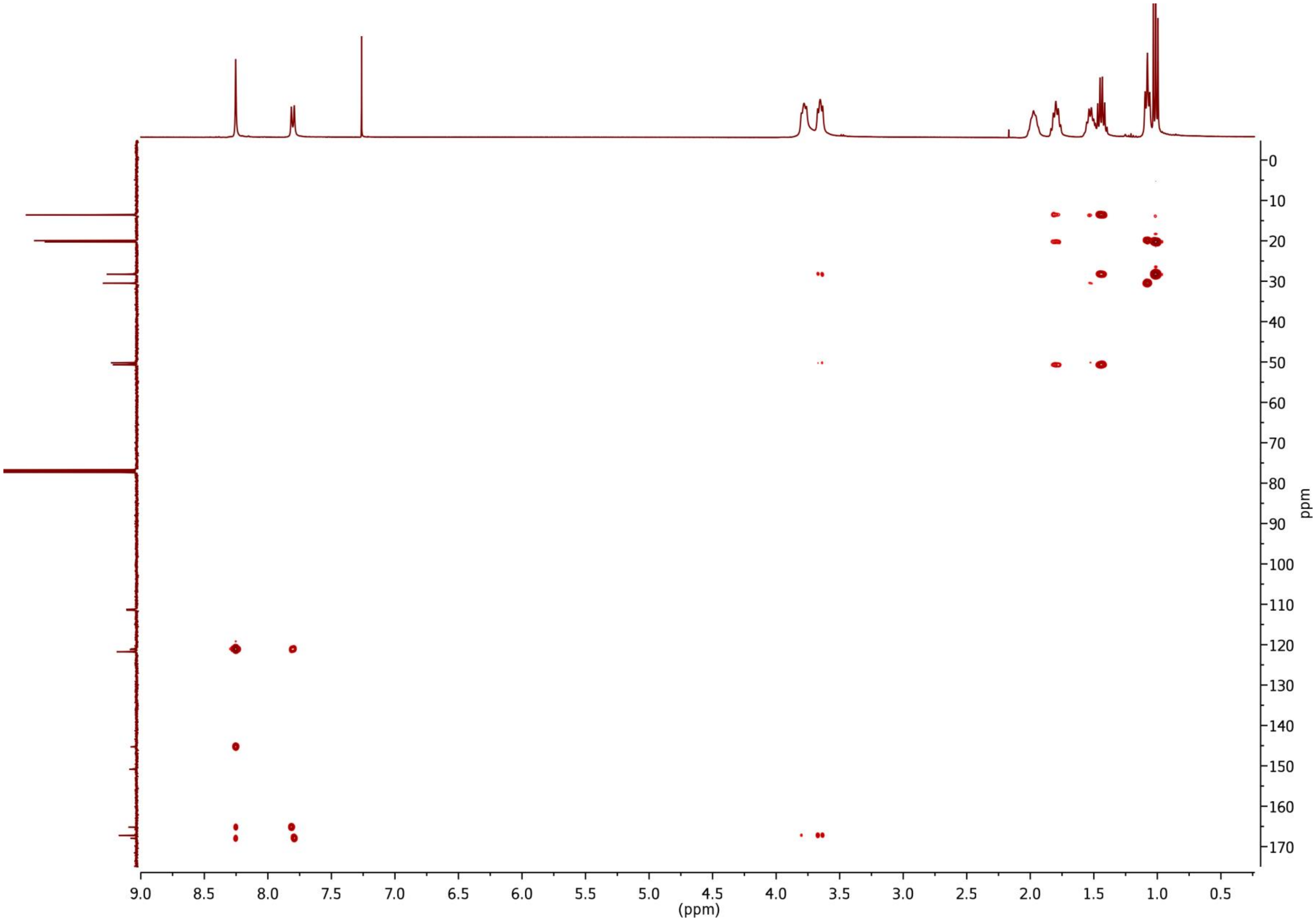

Figure S58. $^{1}H/^{13}C$ HMBC NMR spectrum in $CDCl_3$ at 25ºC

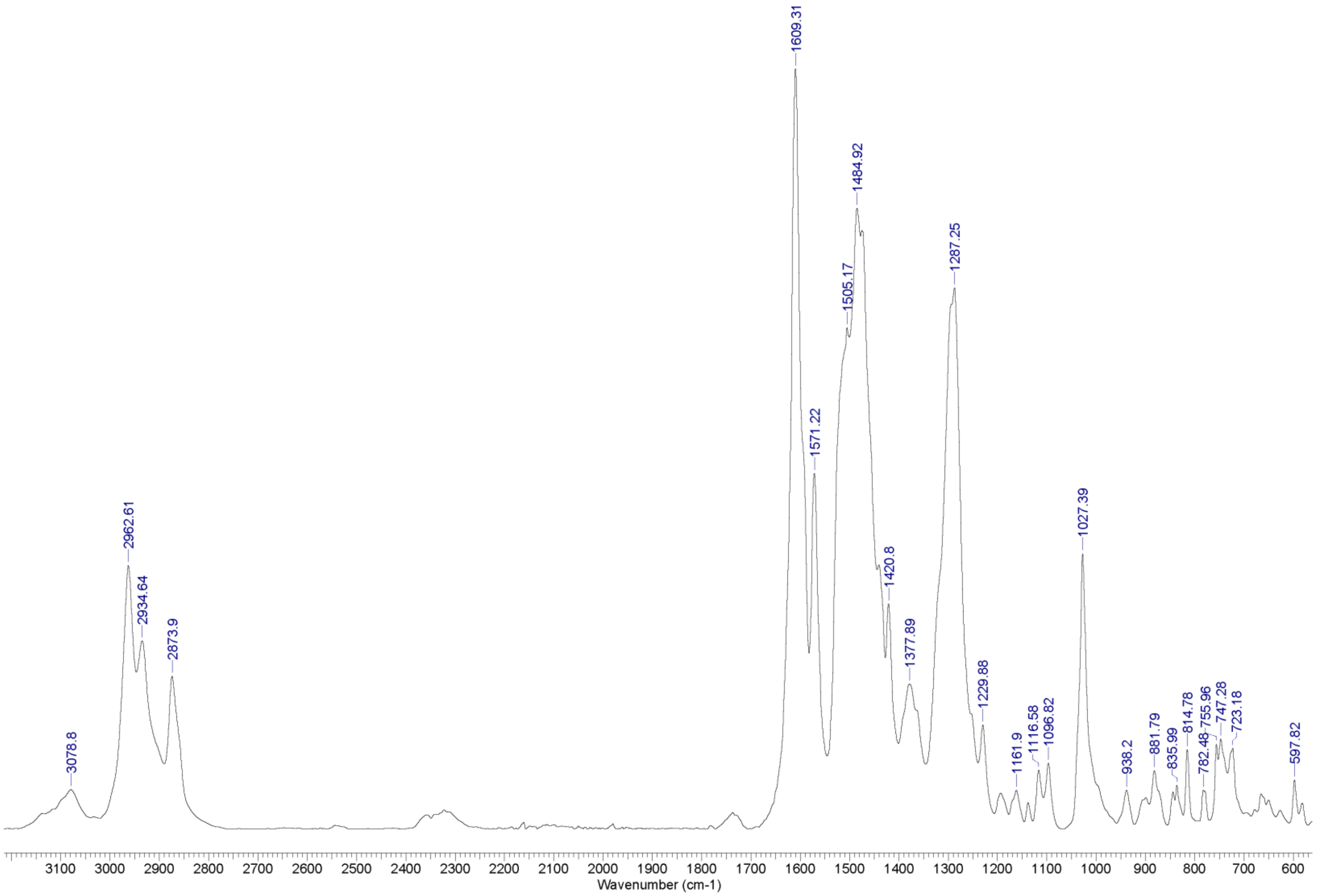

Figure S59. Solid-state IR spectrum at 25ºC

2. X-ray analysis data

Table S1. Crystallographic data for **7a** and **7c, 7a•**$La(NO_3)_3$, **7a•**$Nd(NO_3)_3$ and **7a•**$Eu(NO_3)_3$,

	**7a**	**7c**	**7a•La(NO₃)₃**	**7a•Nd(NO₃)₃**	**7a•Eu(NO₃)₃**
Empirical formula	$C_{30}H_{40}F_2N_4O$	$C_{38}H_{40}F_2N_4O_2$	$C_{34}H_{46}F_2LaN_9O_{11}$	$C_{34}H_{46}F_2NdN_9O_{11}$	$C_{34}H_{46}F_2EuN_9O_{11}$
Formula weight	526.66	622.74	933.71	939.04	946.76
Temperature (K)	110	100	100	110	110
Crystal system	Triclinic	Monoclinic	Monoclinic	Monoclinic	Monoclinic
Space group	P-1	$P2_1/c$	$P2_1/c$	P21/c	$P2_1/c$
a (Å)	8.6883(10)	11.261(6)	9.1881(8)	9.1881(8)	9.1770(9)
b (Å)	9.1327(11)	13.610(7)	25.461(2)	25.461(2)	25.434(3)
c (Å)	17.932(2)	21.358(10)	17.639(2)	17.639(2)	17.5925(16)
α (º)	88.166(4)	90	90	90	90
β (º)	77.855(4)	95.572(15)	92.330(3)	92.330(3)	92.357(3)
γ (º)	81.117(4)	90	90	90	90
Volume ($Å^3$)	1374.3(3)	3258(3)	4122.9(7)	4122.9(7)	4102.7(7)
Z(Z’)	2(1)	4(1)	4(1)	4(1)	4(1)
d_{calc}, $g \cdot cm^{-3}$	1.273	1.270	1.504	1.513	1.533
μ, cm^{-1}	0.9	0.87	11.12	13.35	16.04
F(000)	564	1320	1904	1916	1928
$2\theta_{max}$, °	58	52	58	58	58
Complentess to Θ_{max}	0.999	0.999	0.999	0.999	0.998
Refl. collected	20747	19620	37287	40657	39319
Refl. unique (Rint)	7303	6390	10960	10900	10887
Refl. with I > 2σ(I)	5114	2325	7843	8071	8747
Parameters	347	430	530	520	520
R_1 with I>2σ(I)	0.0501	0.0843	0.0630	0.0430	0.0351
wR_2 (all data)	0.1235	0.2259	0.1672	0.0964	0.0855
Goodness-of-fit on F^2	1.053	0.941	1.028	1.021	1.050
Largest difference in peak / hole (e/$Å^3$)	0.365/-0.288	0.364/-0.286	1.639/-0.993	0.666/-0.575	0.743/-0.693
CCDC	2183813	2183814	2183815	2183816	2183817
